# Synthesis of Novel Pyridine‐Carboxylates as Small‐Molecule Inhibitors of Human Aspartate/Asparagine‐β‐Hydroxylase

**DOI:** 10.1002/cmdc.202000147

**Published:** 2020-05-26

**Authors:** Lennart Brewitz, Anthony Tumber, Armin Thalhammer, Eidarus Salah, Kirsten E. Christensen, Christopher J. Schofield

**Affiliations:** ^1^ Chemistry Research Laboratory University of Oxford 12 Mansfield Road Oxford OX1 3TA UK; ^2^ Chemical Crystallography Chemistry Research Laboratory University of Oxford 12 Mansfield Road Oxford OX1 3TA UK

**Keywords:** demethylases, hydroxylase inhibitors, aspartate/asparagine-beta-hydroxylase, oxygenases, pyridine-2,4-dicarboxylic acid

## Abstract

The human 2‐oxoglutarate (2OG)‐dependent oxygenase aspartate/asparagine‐β‐hydroxylase (AspH) is a potential medicinal chemistry target for anticancer therapy. AspH is present on the cell surface of invasive cancer cells and accepts epidermal growth factor‐like domain (EGFD) substrates with a noncanonical (i. e., Cys 1–2, 3–4, 5–6) disulfide pattern. We report a concise synthesis of C‐3‐substituted derivatives of pyridine‐2,4‐dicarboxylic acid (2,4‐PDCA) as 2OG competitors for use in SAR studies on AspH inhibition. AspH inhibition was assayed by using a mass spectrometry‐based assay with a stable thioether analogue of a natural EGFD AspH substrate. Certain C‐3‐substituted 2,4‐PDCA derivatives were potent AspH inhibitors, manifesting selectivity over some, but not all, other tested human 2OG oxygenases. The results raise questions about the use of pyridine‐carboxylate‐related 2OG analogues as selective functional probes for specific 2OG oxygenases, and should aid in the development of AspH inhibitors suitable for *in vivo* use.

## Graphical abstract

**Sights on a new target**: A concise synthesis of C‐3‐substituted pyridine‐2,4‐dicarboxylic acid (2,4‐PDCA) derivatives is reported. The derivatives are potent inhibitors of the human 2‐oxoglutarate‐dependent oxygenase aspartate/asparagine‐β‐hydroxylase (AspH), which is a potential medicinal chemistry target for cancer therapy. This work constitutes a step towards the development of AspH inhibitors suitable for *in vivo* use.

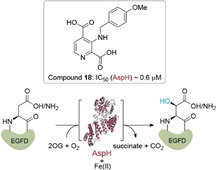

## Introduction

Following the pioneering identification of the procollagen prolyl‐residue hydroxylases (CPHs) as Fe^II^ and 2‐oxoglutarate (2OG)‐dependent oxygenases,^1^ related enzymes, which play important roles in human biology, have emerged; some of these are validated medicinal chemistry targets.^2^ Human 2OG oxygenases have roles in lipid metabolism,^3^ processing proteins destined for secretion,^1^, ^2^ histone/chromatin modifications,^4^ DNA/RNA damage repair,^5^ and hypoxia sensing.^6^

**Figure 1:**
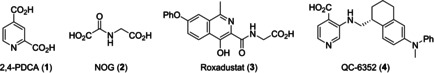
Structures of the broad‐spectrum 2OG oxygenase inhibitors 2,4‐PDCA (**1**) and NOG (**2**) and the PHD selective inhibitor roxadustat (**3**)11a and the KDM4‐selective inhibitor QC‐6352 (**4**).^14^

Inhibition of the CPHs was pursued for the treatment of fibrotic diseases, but was suspended due to toxicity issues.^7^ The CPH inhibition work pioneered the use of 2OG analogues/competitors such as pyridine‐2,4‐dicarboxylic acid (2,4‐PDCA or 2,4‐lutidinic acid, **1**; Figure 1)^8^ and *N*‐oxalylglycine (NOG, **2**, Figure 1).^9^ This approach has been successfully applied to the development of the 2OG‐dependent hypoxia‐inducible transcription factor‐α (HIF‐α) prolyl‐residue hydroxylases (PHDs) inhibitors,^10^ some of which are approved for use in the treatment of anemia in patients suffering from chronic kidney disease (e. g., roxadustat **3**,^11^ Figure 1). With the notable exception of mildronate (meldonium),^12^ an inhibitor of γ‐butyrobetaine dioxygenase (BBOX),^13^ most 2OG oxygenase inhibitors are active‐site Fe^II^ ligands/2OG competitors.^10^ Examples include pyridine‐carboxylate derivative QC‐6352 (**4**, Figure 1), which inhibits the Jmjc lysine‐specific demethylase 4 (KDM4, JMJD2) enzymes and which shows anti‐proliferative effects in cancer models.^14^

The human 2OG oxygenase aspartate/asparagine‐β‐hydroxylase (AspH, BAH, HAAH) catalyses the hydroxylation of conserved Asp and Asn residues in epidermal growth factor‐like domains (EGFDs),^15^ which include the extracellular domains of the notch receptor and its ligands.^16^ AspH levels are upregulated in multiple cancers (e. g., hepatocellular carcinoma^17^ and pancreatic cancer^18^). AspH is reported to be translocalised from the endoplasmic reticulum (ER) to the cell surface membrane resulting in enhanced tumour invasiveness and a diminished patient survival rate.17b, 19 AspH is also strongly hypoxically regulated^20^ and may have a role in hypoxia sensing.^21^ AspH is thus an interesting potential target from a cancer medicinal chemistry perspective.^22^

Only a few structure‐activity relationship (SAR) studies directed at identifying AspH inhibitors have been reported. Initial studies identified several nonselective small‐molecule AspH inhibitors in cell‐based experiments^25^ and l‐ascorbate based AspH inhibitors have been used in cellular and animal experiments.22a, 26 Studies focusing on the development of nitrogen‐containing heteroaromatic scaffolds for AspH inhibition have not been reported despite the prominence of these scaffolds in approved active pharmaceutical ingredients (APIs), including anticancer drugs and 2OG oxygenase inhibitors.^27^ The discrepancy between the potential of a AspH as a medicinal chemistry target and the limited effort of developing efficient small‐molecule AspH inhibitors for clinical use likely relates to the lack of efficient assays for isolated AspH.

**Figure 2:**
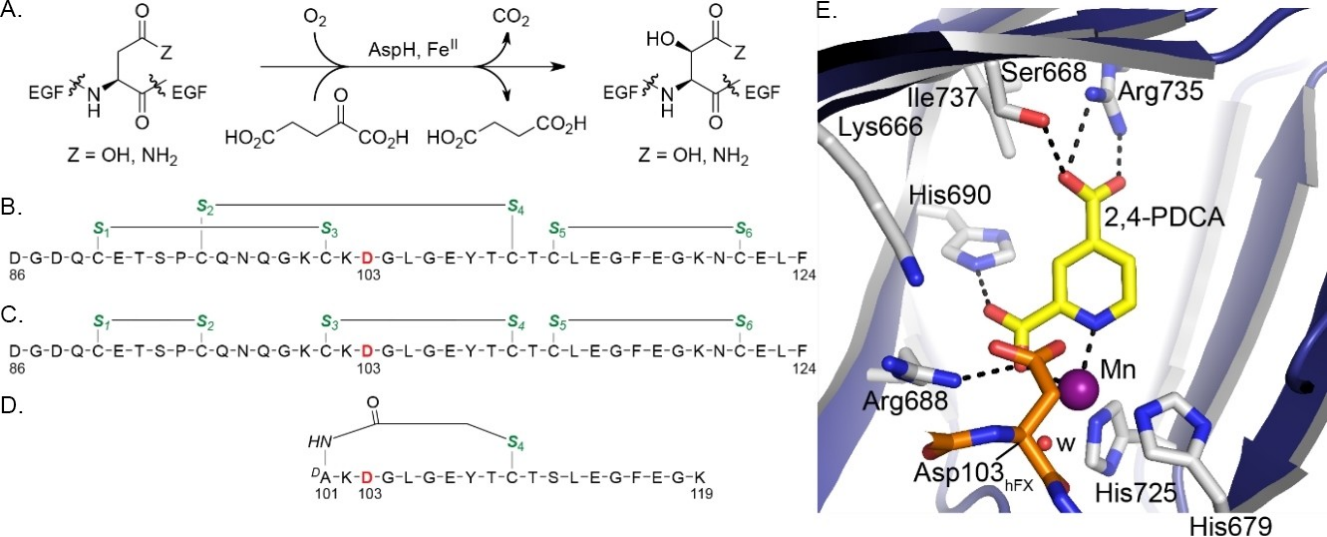
AspH catalyses the hydroxylation of the Asp/Asn‐residues of specific EGFD disulfide isomers. A) Scheme for the AspH‐catalysed hydroxylation of Asp/Asn‐residues in EGFDs. B) The canonical (Cys 1–3, 2–4, 5–6) EGFD1 disulfide pattern of human coagulation factor X (hFX amino acids 86–124); the AspH hydroxylation site (Asp103_hFX_) is in red, cystine thiols in green. C) The noncanonical (Cys 1–2, 3–4, 5–6) hFX EGFD1 disulfide isomer. D) A cyclic thioether peptide (hFX‐CP_101‐119_) mimicking the central noncanonical (Cys 3–4) hFX EGFD1 disulfide fold containing the AspH hydroxylation site Asp103_hFX_ (red).^23^ E) Close‐up of the AspH active site in the AspH:2,4‐PDCA crystal structure (PDB ID: 5JTC).^24^ The key residues engaged in 2,4‐PDCA (Ser668, Arg688, His690, and Arg735) and metal binding (His679, His725) are shown. A pocket formed by AspH residues Ser668, Ile737, His690, Arg688, and Lys666 adjacent to the C‐3 position of 2,4‐PDCA is sufficiently large to accommodate substituents at the C‐3 position of 2,4‐PDCA. Colours: violet/grey: His_6_‐AspH_315‐758_; yellow: carbon‐backbone of 2,4‐PDCA; orange: carbon‐backbone of a synthetic hFX EGFD1‐derived peptide; purple: Mn; red: oxygen; blue: nitrogen. w: water.

Recently, we reported crystal structures of recombinant human AspH using truncated constructs.^23^ Together with biochemical studies, these reveal that AspH accepts EGFDs with a noncanonical (Cys 1–2, 3–4, 5–6; Figure 2C) disulfide pattern, rather than the well‐characterised canonical (Cys 1–3, 2–4, 5–6; Figure 2B) disulfide pattern.^23^ These findings prompted us to develop a semi‐automated high‐throughput assay using solid phase extraction (SPE) coupled to mass spectrometry (MS) and stable disulfide analogues (e. g., hFX‐CP_101‐119_; Figure 2D) to monitor AspH activity.^24^

Here, we report SAR studies on nitrogen‐containing heteroaromatic small‐molecule AspH inhibitors. Novel C‐3‐substituted derivatives of 2,4‐PDCA (**1**), which is a potent, but non‐selective AspH inhibitor competing with 2OG for 2OG binding in the AspH active site (Figure 2E),^24^, ^25^ were synthesised and their inhibitory properties investigated using SPE‐MS inhibition assays against AspH and other human 2OG oxygenases. The results reveal the potential for potent and selective AspH inhibition, and raise questions regarding the selectivity of PDCA‐type and related 2OG oxygenase inhibitors that have been reported in the literature.

## Results and Discussion

### Inhibition of AspH by isomers and derivatives of 2,4‐PDCA

**Figure 3:**
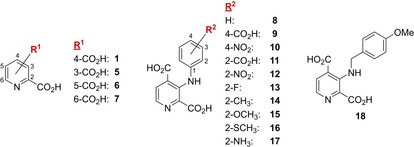
Structures of 2,*x*‐PDCAs (*x*=3‐6; **1** and **5–7**) and C‐3‐substituted 2,4‐PDCA derivatives (**8**–**18**) initially screened for AspH inhibition.

The mechanism by which 2,4‐PDCA (**1**) inhibits AspH involves competition with 2OG for binding at the AspH active site.^24^ 2,4‐PDCA (**1**) coordinates to the active site Fe^II^ through bidentate chelation with its nitrogen atom and an oxygen atom of the C‐2 carboxylate as evidenced by crystallography (PDB ID: 5JTC; Figure 2E).^24^ Isomers of 2,4‐PDCA lacking the C‐2 carboxylate do not inhibit AspH.25a For SAR studies, we therefore fixed the C‐2 2,4‐PDCA carboxylate group whilst varying the position of the other carboxylate. Half‐maximum inhibitory concentrations (IC_50_) of three 2,4‐PDCA regioisomers (**5**‐**7**; Figure 3) were determined using SPE‐MS high‐throughput *in vitro* AspH inhibition assays conducted at 2OG, substrate, and Fe^II^ concentrations close to their *K*_m_ values.^21^

Efficient inhibition of AspH by 2,3‐PDCA (quinolinic acid, **5**), a natural product of the kynurenine pathway of tryptophan metabolism,^28^ was observed (IC_50_ ∼0.5 μM, Table 1), whereas 2,5‐PDCA (**6**) did not inhibit AspH (Table 1). However, the potency of 2,3‐PDCA (**5**) was lower than that of 2,4‐PDCA (**1**) by an order of magnitude (Table 1). Apparently, inverse behaviour with inhibitor potency has been observed in inhibition studies with KDM4E, which is inhibited by 2,5‐PDCA (**6**), but not by 2,3‐PDCA (**5**).^29^ This observation indicates that different PDCA regioisomers might selectively inhibit different subclasses of the human 2OG oxygenases. This proposal is further supported by the findings that 2,5‐PDCA (**6**) inhibits the CPHs with greater potency than 2,4‐PDCA (**1**), whereas 2,5‐PDCA (**6**) is significantly less potent than 2,4‐PDCA (**1**) in inhibiting the PHDs.^8^, ^30^

**Table 1:** The Inhibition of human AspH by regioisomers and C‐3 derivatives of 2,4‐PDCA.

	Compound	IC_50_ [μM]^[a]^
1	2,4‐PDCA (**1**)	0.03 ±0.01
2	2,3‐PDCA (**5**)	0.51 ±0.05
3	2,5‐PDCA (**6**)	inactive
4	2,6‐PDCA (**7**)	5.48 ±0.75
5	**8**	38.8 ±7.0
6	**9**	inactive
7	**10**	inactive
8	**11**	10.4 ±1.8
9	**12**	16.1 ±4.0
10	**13**	22.1 ±1.5
11	**14**	12.9 ±1.5
12	**15**	13.8 ±0.2
13	**16**	16.1 ±2.5
14	**17**	4.67 ±0.29
15	**18**	0.58 ±0.06

[a] Mean average of two independent runs (*n*=2; mean ± standard deviation, SD). AspH inhibition assays were performed as described in the Experimental Section using 50 nM His_6_‐AspH_315‐758_ and 1.0 μM hFX‐CP_101‐119_ (Figure 2D) as a substrate.

Moderate inhibition of AspH activity by 2,6‐PDCA (**7**) was observed (IC_50_ ∼5.5 μM, Table 1). Previous studies have indicated that 2,6‐PDCA (**7**) can inhibit 2OG oxygenases by (potential) active‐site Fe^II^ binding as well as by chelating Fe^II^ in solution, thus lowering available iron concentrations.^8^, ^29^

Although 2,4‐PDCA (**1**) is more potent than its regioisomers **5–7**, it lacks selectivity for AspH over other human 2OG oxygenases, in particular the Jmjc KDMs.^10^ SAR studies were therefore initiated to explore the selectivity of 2,4‐PDCA derivatives. Based on the crystal structure of 2,4‐PDCA (**1**) in complex with AspH (PDB ID: 5JTC),^24^ we proposed that introduction of substituents at the C‐3 position of 2,4‐PDCA might increase inhibitor selectivity (Figure 2E). As we had previously synthesised C‐3‐substituted 2,4‐PDCA derivatives **8–18** (Figure 3) as KDM4E (JMJD2E) inhibitors,^31^ their potential inhibition of AspH was investigated (Table 1). The inhibition of AspH by 2,4‐PDCA bearing an aniline substituent at C‐3 (**8**) was >1000 fold less than the parent 2,4‐PDCA **1** (IC_50_∼38.8 μM, Table 1). Introducing electron‐deficient substituents at the aniline *para* position of compound **8** resulted in complete loss of activity (**9**, **10**), while electron‐deficient substituents at the aniline *ortho* position manifested moderate potency (**11**–**13**). Electron‐donating substituents at the aniline *ortho* position (**14**–**17**) did not improve potency relative to **8**; only in the case of pyridine **17** was a notable improvement observed (IC_50_∼4.7 μM, Table 1).

The most pronounced effect on inhibitor potency was observed when the aniline substituent at the C‐3 position of 2,4‐PDCA was changed to 4‐methoxybenzylamine: Compound **18** inhibited AspH efficiently, its IC_50_ value was only 20 fold above that of 2,4‐PDCA **1** (IC_50_∼0.6 μM, Table 1). The significantly higher potency of **18** compared to 2,4‐PDCA derivatives **8–17** may relate to the increased rotational freedom enabled by the additional methylene‐unit in **18**.

Notably, 2,4‐PDCA (**1**) is a potent KDM4E inhibitor (IC_50_=0.44 μM), however, the 2,4‐PDCA derivative **18** is only a weak reported KDM4E inhibitor (IC_50_=41 μM),^31^ indicating that substituents at the C‐3 position of 2,4‐PDCA might enable selective AspH inhibition.

### An improved synthesis of C‐3 aminoalkyl‐substituted 2,4‐PDCA derivatives

**Scheme 1:**
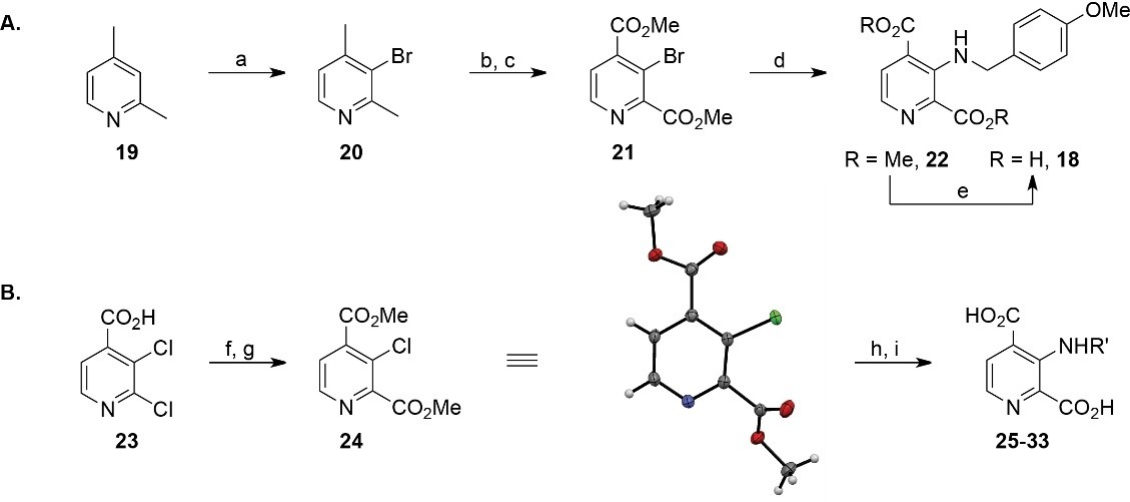
A) The reported synthesis of C‐3 aminoalkyl‐substituted 2,4‐PDCA derivative **18** 31 compared with B) the new route to C‐3 aminoalkyl‐substituted 2,4‐PDCA derivatives. a) Br_2_, 20 % oleum, 165 °C, 35 %; b) KMnO_4_, NaOH, H_2_O, reflux; c) H_2_SO_4_, MeOH, reflux, 45 % (2 steps); d) amine (1.2 equiv), Pd_2_(dba)_3_ (2 mol%), Xantphos (6 mol%), Cs_2_CO_3_, toluene, 110 °C, 69 %; e) NaOH, MeOH/H_2_O, then: HCl, 96 %; f) SOCl_2_, MeOH, reflux, 89 %; g) CO (1.5 atm), Cl_2_Pd‐*rac*‐BINAP (1 mol%), *i*Pr_2_NEt, MeOH, 100 °C (sand bath temperature, sealed flask), 90 %; h) alkylamine (H_2_NR′, 1.5 equiv), Pd(OAc)_2_ (4 mol%), Xantphos (6 mol%), pyridine, toluene, 190 °C (sand bath temperature, sealed flask), 32–71 %; i) LiOH, MeOH/H_2_O, 0 °C to RT, then: Dowex® 50XW8, 61–88 %. Crystal structure colour code: grey: carbons; white: hydrogens; blue: nitrogen; red: oxygens; green: chlorine.

Having identified pyridine **18** as a potent and selective (with respect to KDM4E) AspH inhibitor, we aimed to synthesise other C‐3 aminoalkyl‐substituted 2,4‐PDCA derivatives to generate further SAR data and to investigate the selectivity of the inhibitor series with respect to other human 2OG oxygenases. However, the reported synthesis of C‐3‐substituted 2,4‐PDCA derivatives employs harsh reaction conditions and non‐selective reactions as manifested by low overall yields (Scheme 1A).^31^ Moreover, the HCl adduct of pyridine **18** slowly degraded over time, even when stored at −20 °C.

An optimised route for synthesis of C‐3 aminoalkyl‐substituted 2,4‐PDCA derivatives was developed which shortens the reported synthesis by one step and overcomes the described shortcomings (Scheme 1B). Thus, commercial 2,3‐dichloroisonicotinic acid (**23**) was converted into its methyl ester, which was then submitted to regioselective Pd‐catalysed carbonylation^32^ yielding dimethyl ester **24** (90 %, over two steps).

1‐ and 2‐dimensional NMR analysis of the crude reaction product **24** indicated full substrate conversion with exclusive formation of the desired regioisomer in the carbonylation reaction as confirmed by single‐crystal X‐ray diffraction analysis of purified **24**.

Buchwald‐Hartwig amination^33^ of dimethyl ester **24** with alkylamines required optimisation due to the reduced reactivity of its C−Cl bond compared to the C−Br bond of dimethyl ester **21** (Scheme 1). Undesired reaction pathways (ester saponification and amide bond formation) occurring instead of Buchwald‐Hartwig amination under the reported conditions^31^ were circumvented by replacing the inorganic base Cs_2_CO_3_ with pyridine. Conversion was further improved by substituting the palladium precatalyst Pd_2_(dba)_3_ with Pd(OAc)_2_, retaining Xantphos^34^ as ligand. Both linear and α‐branched alkylamines were successfully applied in the amination reactions whereas α,α‐disubstituted alkylamines did not react, presumably due to steric hindrance. The use of alkylamines containing heteroaromatic moieties (e. g., thiophene, furan) was unsuccessful. Crystallographic analysis confirmed the anticipated regioselectivity of the Buchwald‐Hartwig reaction (Figure S1 and Table S1 in the Supporting Information).

**Figure 4:**
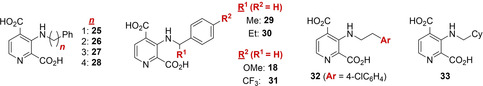
Structures of C‐3 aminoalkyl‐substituted 2,4‐PDCA derivatives **18** and **25–33** (pyridines **29** and **30** were prepared as racemic mixtures).

The intermediate dimethyl esters were subjected to lithium hydroxide‐mediated saponification to afford the desired C‐3 aminoalkyl‐substituted 2,4‐PDCA derivatives **18** and **25–33** (Figure 4) in good yields; excess base was removed by acidic ion exchange chromatography to yield the salt‐free inhibitors which were stable as solids and in aqueous solution (see the Experimental Section).

### SAR studies on the inhibition of AspH by C‐3 aminoalkyl‐substituted 2,4‐PDCA derivatives

**Figure 5:**
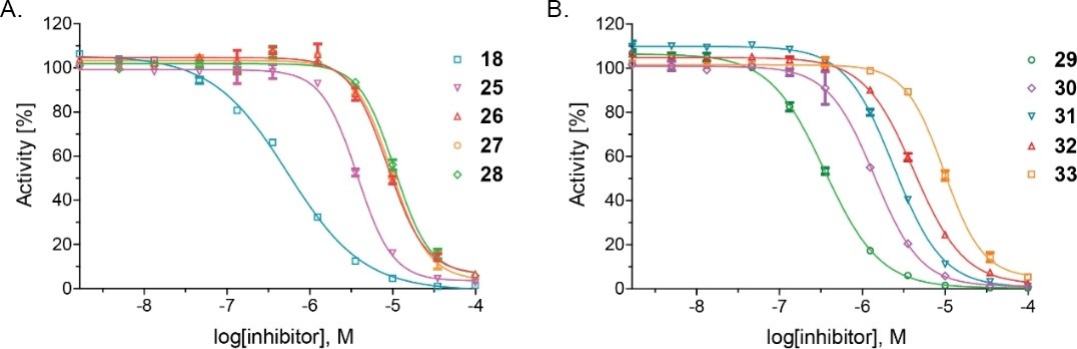
Representative dose‐response curves used to determine IC_50_ values for C‐3 aminoalkyl‐substituted 2,4‐PDCA derivatives **18** and **25–33**. Two dose‐response curves each composed of technical duplicates were independently determined by using SPE‐MS AspH inhibition assays and manifest high Z’‐factors^36^ and signal‐to‐noise ratios (Figure S2). Assays were performed as described in the Experimental Section by using 50 nM His_6_‐AspH_315‐758_, 1 μM hFX‐CP_101‐119_ (Figure 2D), 100 μM l‐ascorbic acid (LAA), 3 μM 2OG, and 2 μM Fe^II^ in 50 mM HEPES buffer (pH 7.5, 20 °C). Data are shown as the mean average of two technical duplicates (*n*=2; mean ± SD).

The SAR studies reveal that increasing the length of the C‐3 aminoalkyl chain from methylene to butylene has a detrimental effect on potency (**25**–**28**, Figure 5 and Table 2). Introducing a substituent α to the amino group of the alkyl chain increases potency; the effect appears to be more pronounced for shorter alkyl groups (methyl, **29**, IC_50_∼0.3 μM versus ethyl, **30**, IC_50_∼1.2 μM; Table 2). Note that for pyridines **29** and **30** racemic mixtures were tested. Potency was reduced with a saturated C‐3 aminoalkyl chain (**33**) compared to a C‐3 aminoalkyl side chain bearing an aromatic residue (**25**). This might relate to increased steric bulk or lack of π‐π stacking effects. Overall *para*‐substituted aromatic moieties in the C‐3 aminoalkyl side chain (**18**, **31**, **32**) increase potency compared to non‐substituted aromatics (**25**, **26**). This effect is more pronounced for electron donating substituents (**18**) compared to electron withdrawing substituents (**31**).

**Table 2:** The inhibition of human AspH by C‐3 aminoalkyl‐substituted 2,4‐PDCA derivatives.

	Compound	IC_50_ [μM]^[a]^
1	**18**	0.58 ±0.06
2	**25**	3.95 ±0.33
3	**26**	7.66 ±1.45
4	**27**	10.91 ±1.83
5	**28**	10.76 ±0.28
6	**29**	0.28 ±0.11
7	**30**	1.22 ±0.26
8	**31**	1.86 ±1.06
9	**32**	3.67 ±0.74
10	**33**	13.13 ±4.84

[a] Mean average of two independent runs (*n*=2; mean ± SD). AspH inhibition assays were performed as described in the Experimental Section by using 50 nM His_6_‐AspH_315‐758_ and 1.0 μM hFX‐CP_101‐119_ (Figure 2D) as a substrate.

### Selectivity studies

To investigate the selectivity of the C‐3 functionalised 2,4‐PDCA derivatives for AspH, their inhibitory activities against the isolated human 2OG oxygenases PHD2, which is a validated medicinal chemistry target,^35^ factor inhibiting HIF‐α (FIH), and the Jmjc histone demethylase KDM4E were determined. The inhibition assays all employed SPE‐MS to help ensure comparability with the AspH results and minimise possible discrepancies due to different assay methods (Table 3).

**Table 3:** The inhibition of human AspH, PHD2, FIH, and KDM4E by selected C‐3 functionalised 2,4‐PDCA derivatives.

Inhibitor	IC_50_ AspH [μM]^[a,b]^	IC_50_ PHD2 [μM]^[a,c]^	IC_50_ FIH [μM]^[a,d]^	IC_50_ KDM4E [μM]^[a,e]^
1	0.03 ±0.01	5.29±3.35	5.03±2.06	0.18±0.01^39^
17	4.67 ±0.29	2.60 ±1.20	6.59 ±0.05	0.43 ±0.05
18	0.58 ±0.06	>50	43.0 ±3.9	2.45 ±0.59
25	3.95 ±0.33	>50	>50	2.36±0.13
26	7.66 ±1.45	>50	>50	5.71±0.04
29	0.28 ±0.11	>50	14.9 ±1.5	0.99 ±0.31
30	1.22 ±0.26	>50	46.1 ±3.2	3.69 ±0.85
31	1.86 ±1.06	>50	>50	3.57 ±0.38
32	3.67 ±0.74	>50	26.1 ±3.9	4.14±0.28

[a] Mean of two independent runs (*n*=2; mean ± SD). [b] Using 50 nM His_6_‐AspH_315‐758_ and 1.0 μM hFX‐CP_101‐119_ (Figure 2D). [c] Using 150 nM PHD2 and 5.0 μM C‐terminal oxygen‐dependent degradation domain fragment (HIF‐1α CODD, amino acids 558–574). [d] Using 150 nM FIH and 5.0 μM C‐terminal transactivation domain fragment (HIF‐1α CAD, amino acids 788–822). [e] Using 150 nM KDM4E and 10.0 μM of a variant of histone 3 (H3_1‐15_K9me3, amino acids 1–15; Experimental Section)^40^. Enzyme inhibition assays were performed as described in the Experimental Section.

Most of the C‐3 aminoalkyl‐substituted 2,4‐PDCA derivatives investigated did not inhibit PHD2, with the exception of **17** which bears an aniline substituent and is a rather weak AspH inhibitor (Table 3). These results are consistent with studies showing that the parent 2,4‐PDCA (**1**) is a relatively weak inhibitor of PHD2.^30^

FIH was substantially inhibited by C‐3 aminoalkyl‐substituted 2,4‐PDCA derivatives **17** and **29** (IC_50_∼6.6 and 14.9 μM, respectively; Table 3), whereas all the other investigated 2,4‐PDCA derivatives displayed only moderate potency (Table 3). Thus, most inhibitors displayed >40 fold selectivity for AspH with the exceptions of **17** (non‐selective) and **32** (ca. seven‐fold selective for AspH; Table 3). Analysis of a crystal structure of FIH complexed with Fe^II^ and 2,4‐PDCA^37^ suggests that 2,4‐PDCA derivatives **17** and **29** likely inhibit FIH by replacing 2OG in the active site with the C‐3 substituent facing into a hydrophobic region as observed for the (partially) selective FIH inhibitor *N*‐oxalyl‐D‐phenylalanine (NOFD).^38^

The inhibition of KDM4E activity by the C‐3 aminoalkyl‐substituted 2,4‐PDCA derivatives might have been considered likely to be relatively weak, as the 2,4‐PDCA C‐3 derivative **18** has been previously described to be a significantly less potent KDM4E inhibitor (IC_50_∼41 μM) compared to the C‐3 aniline‐substituted 2,4‐PDCAs **8–17**.^31^ However, C‐3 aminoalkyl‐substituted 2,4‐PDCA derivatives **18**, **25**, **26**, and **29–32** inhibited KDM4E efficiently (Table 3) with IC_50_ values as low as ∼1.0 μM for **29**. The results may in part reflect improved SPE‐MS based KDM4E inhibition assays^39^, ^40^, ^41^ compared to the previously used FDH‐coupled KDM4E inhibition assays requiring higher enzyme and cofactor/(co‐)substrate concentrations.^31^ Selectivities for AspH versus KDM4E did not exceed 4 : 1 and, considering that KDM4E assay concentrations were approximately three times higher than those of AspH, the selectivity for AspH may even be lower. The results also indicate that structurally similar KDM4 inhibitors lacking the C‐2 carboxylate moiety, which display high potency against KDM4E,^14^, 41a could inhibit AspH activity substantially. This observation might, at least in part, account for the observed antiproliferative effects of some of these compounds observed in cell‐based experiments.^14^ Considering the prominence of the isonicotinic acid scaffold for the development of small‐molecule KDM4 inhibitors,^42^ concerns on the selectivity of these compounds arise.

## Conclusions

The results described herein demonstrate that it is possible to efficiently modulate the potent AspH inhibitor 2,4‐PDCA (**1**, Figure 1) using a novel four‐step synthetic sequence (Scheme 1), which includes two palladium‐catalysed reactions, to identify compounds of approximately equal potency. Out of the eight most potent identified AspH inhibitors, **29** is the most efficient AspH inhibitor (IC_50_∼0.3 μM); C‐3 aminoalkyl‐substituted 2,4‐PDCA derivative **26** (IC_50_∼7.7 μM) is the least efficient (Table 3).

Although further work is required, the results reveal that it should be possible to develop small‐molecule AspH inhibitors of suitable potency and selectivity for *in vivo* studies aimed at validating AspH as a medicinal chemistry target to develop novel cancer therapeutics and at exploring the function of EGFD hydroxylation in greater detail. Considering that AspH is translocated to the cell membrane of invasive cancer cells,^19^ the dicarboxylic acid motif of the 2,4‐PDCA derivatives synthesised might be beneficial to minimise the cell‐wall permeability of the inhibitors and thus reducing the possibility of undesired off‐target effects through inhibiting other 2OG oxygenases, such as the Jmjc KDMs, in cells.

In terms of the selectivity of the C‐3‐substituted 2,4‐PDCA derivatives, the results presented here demonstrate a substantial overlap between AspH inhibition with that of KDM4E (Table 3). By implication, this will likely extend to at least the other Jmjc KDM4 enzymes (i. e., human KDM4A‐D). Crystallographic analysis of the active site structures of the two types of 2OG oxygenases suggest that it should be possible to develop selective inhibitors for AspH or the KDM4 class of 2OG oxygenases.^23^, ^43^ Now that a reliable assay for isolated AspH has been established^24^ and Jmjc KDMs including KDM4E are actively being pursued as medicinal chemistry targets with several pyridine‐based and related small‐molecule inhibitors for cancer treatment,^44^ AspH should be included in Jmjc KDM selectivity counter‐screens in order to develop improved inhibitors and safe medicines.

It should also be noted that 2,4‐PDCA (**1**)^24^ and 2,3‐PDCA (quinolinic acid, **5**) are potent AspH inhibitors. The latter observation raises the possibility of natural inhibition of AspH by small‐molecules such as quinolinic acid. A prodrug form of 2,4‐PDCA might be useful in probing AspH function *in vivo* bearing in mind possible “off‐target” effects through the inhibition of other 2OG oxygenases including the Jmjc KDMs (Table 3). In this regard, it is notable that dimethyl *N*‐oxalylglycine (DMOG), a prodrug form of NOG (**2**, Figure 1), has been used to mimic the cellular hypoxic response by inhibiting the PHDs, though it inhibits other 2OG oxygenases and other enzymes.^20^ Like 2,3‐PDCA, NOG is a natural product, at least in plants.^45^ NOG is a less potent inhibitor of AspH and the Jmjc KDMs than 2,4‐PDCA;^24^ conversely, 2,4‐PDCA inhibits the PHDs significantly less efficient than AspH (Table 3). Thus, providing care is taken to consider “off‐target” effects, incompletely selective small‐molecule inhibitors can be of use in biological functional assignment work.

## Experimental Section

**General information**: All reagents were from commercial sources (Sigma‐Aldrich, Inc.; Fluorochem Ltd; Tokyo Chemical Industries; Alfa Aesar) and used as received unless otherwise stated. 2,4‐PDCA (**1**) and its regioisomers (**5**–**7**) were from Sigma‐Aldrich. The synthesis and characterisation of 2,4‐PDCA derivatives **8**–**17** has been described elsewhere.^31^ Anhydrous solvents were from Sigma‐Aldrich, Inc. and kept under an atmosphere of nitrogen. Solvents, liquids, and solutions were transferred using nitrogen‐flushed stainless steel needles and syringes. All reactions were carried out under an atmosphere of nitrogen unless stated otherwise. Milli‐Q ultrapure (MQ‐grade) water was used for buffers; LCMS grade solvents (Merck) were used for solid phase extraction (SPE)‐MS.

Purifications were performed using an automated Biotage Isolera One purification machine (wavelength monitored: 254 and 280 nm) equipped with pre‐packed Biotage® SNAP KP‐Sil or Biotage® SNAP Ultra flash chromatography cartridges. The cartridge size and solvent gradients (in column volumes, CV) used, are specified in the individual experimental procedures. HPLC grade solvents (ethyl acetate and cyclohexane; Sigma‐Aldrich Inc.) were used for purifications, reaction work‐ups, and extractions.

Thin‐layer chromatography (TLC) was carried out using Merck silica gel 60 F_254_ TLC plates and visualised under UV light. Melting points (MP) were determined using a Stuart SMP‐40 automated melting point apparatus. Infrared (IR) spectroscopy was performed using a Bruker Tensor‐27 Fourier transform infrared (FTIR) spectrometer. High‐resolution mass spectrometry (HRMS) was performed using electro‐spray ionisation (ESI) mass spectrometry (MS) in the positive or negative ionisation modes employing a Thermo Scientific Exactive mass spectrometer (ThermoFisher Scientific); data are presented as a mass‐to‐charge ratio (*m/z*) in Daltons.

Single crystal X‐ray diffraction data were collected using an Oxford Diffraction SuperNova diffractometer (Rigaku). Structures were solved using SUPERFLIP software^46^ before refinement with the CRYSTALS software suite.^47^ Crystallographic data can be obtained from the Cambridge Crystallographic Data Centre (CCDC 1988141–42).

Nuclear magnetic resonance (NMR) spectroscopy was performed using a Bruker AVANCE AVIIIHD 600 machine equipped with a 5 mm BB‐F/1H Prodigy N2 cryoprobe. Chemical shifts for protons are reported in parts per million (ppm) downfield from tetramethylsilane and are referenced to residual protium in the NMR solvent (CDCl_3_: *δ*=7.28 ppm; D_2_O: *δ*=4.79 ppm). For ^13^C NMR, chemical shifts are reported in the scale relative to the NMR solvent (i. e., CDCl_3_: *δ*=77.00 ppm). For ^19^F NMR, chemical shifts are reported in the scale relative to CFCl_3_; coupling constants are accurate to 0.1 Hz. The number of C atoms in brackets indicates overlapping signals in ^13^C NMR; chemical shift numbers in brackets indicate close signals that can be differentiated taking into account second respectively third decimal numbers.

### General synthetic procedures

**General Procedure A**: To dimethyl 3‐chloropyridine‐2,4‐dicarboxylate **24** (1.0 equiv), palladium acetate (0.04 equiv), and xantphos^34^ (0.06 equiv) in a capped 5 or 20 mL microwave reaction vial were added anhydrous toluene (0.25 M), pyridine (2.5 equiv), then *N*‐alkylamine (1.5 equiv), at ambient temperature. N_2_ gas was bubbled through the reaction mixture for 15 min, which was then placed into a preheated sand bath (190 °C) and stirred for 22–24 h. The reaction mixture was then cooled to ambient temperature, evaporated under reduced pressure, and purified by column chromatography to afford the desired purified dimethyl C‐3‐aminoalkyl 2,4‐pyridinedicarboxylic acid ester.

**General Procedure B**: To a solution of the dimethyl 3‐aminoalkylpyridine‐2,4‐dicarboxylate (1.0 equiv) in methanol (0.2 M, HPLC grade) was added an aqueous solution of lithium hydroxide (0.4 M, 2.8 equiv) under an ambient atmosphere at 0 °C. The reaction mixture was allowed to slowly warm to ambient temperature overnight (14–18 h); the methanol was then removed under reduced pressure. The solution was extracted three times with CH_2_Cl_2_ (the organic extracts were discarded); the aqueous phase was acidified (pH∼7.7) by adding Dowex® 50XW8 (H^+^‐form, mesh 200–400), filtered, and lyophilised to afford the solid C‐3‐aminoalkyl‐substituted pyridine‐2,4‐dicarboxylic acid. The crude product was sufficiently purified as judged by ^1^H and ^13^C NMR and used without further purification in the biological assays.

### Synthetic procedures and compound characterisations

**Methyl 2,3‐dichloroisonicotinate (23)**: To a solution of 2,3‐dichloroisonicotinic acid (12.3 g, 63.8 mmol, 1.0 equiv) in anhydrous methanol (125 mL) was added dropwise thionyl chloride (7.0 mL, 95.6 mmol, 1.5 equiv), at ambient temperature under a nitrogen atmosphere. The reaction mixture was stirred under reflux for 2 h, then cooled to ambient temperature and concentrated. The residue was dissolved in ethyl acetate, washed twice with saturated aqueous NaHCO_3_ solution, then once with brine; The organic solution was dried over anhydrous Na_2_SO_4_, filtered, evaporated, and purified by column chromatography (100 g KP‐Sil; 80 mL/min; 100 % cyclohexane (2 column volumes, CV) followed by a linear gradient (7 CV): 0→40 % ethyl acetate in cyclohexane) to afford 11.6 g (89 %) of purified methyl ester **23**. White solid, m.p.: 33–35 °C; ^1^H NMR (600 MHz, 300 K, CDCl_3_): *δ*=8.36 (d, *J*=4.9 Hz, 1H), 7.51 (d, *J*=4.8 Hz, 1H), 3.97 ppm (s, 3H); ^13^C NMR (150 MHz, 300 K, CDCl_3_): *δ*=164.0, 151.2, 146.9, 140.4, 128.5, 122.7, 53.2 ppm; IR (film): $\tilde{\nu }$ =3002, 2956, 1738, 1576, 1532, 1447, 1432, 1352, 1294, 1278, 1202, 1158, 1110, 1047, 966 cm^−1^; HRMS (ESI): *m/z* calcd for C_7_H_6_O_2_NCl_2_ [*M*+H]^+^: 205.9770, found: 205.9772.

**Dimethyl 3‐chloropyridine‐2,4‐dicarboxylate (24)**: To a solution of methyl 2,3‐dichloroisonicotinate (**23**) (2.06 g, 10.0 mmol, 1.0 equiv) and dichloro[2,2’‐bis(diphenylphosphino)‐1,1’‐binaphthyl]palladium(II) [(*rac*‐BINAP)PdCl_2_] (80 mg, 0.1 mmol, 0.01 equiv) in anhydrous methanol (50 mL) in a 250 mL J‐Young Schlenk tube was added *N*,*N*‐diisopropylethylamine (2.61 mL, 15.0 mmol, 1.5 equiv) under an ambient temperature. Carbon monoxide gas (synthesis grade) was bubbled through the solution for 10 minutes. ***CAUTION***: Carbon monoxide is a highly toxic and flammable gas; it should be handled in a well‐vented fume cupboard taking appropriate safety measures. The Schlenk tube was then sealed under CO‐pressure (∼1.5–2.0 atm) and placed in a sand bath, which was heated under stirring behind a safety shield at 100 °C for 12 h. The reaction mixture was cooled to ambient temperature, then concentrated and purified by column chromatography (50 g KP‐Sil; 60 mL/min; 100 % cyclohexane (3 CV) followed by a linear gradient (10 CV): 0→30 % ethyl acetate in cyclohexane) to afford 2.16 g (94 %) the pure dimethyl ester **24**. Single‐crystals suitable for X‐ray diffraction analysis were obtained from a concentrated solution of analytically pure **24** in cyclohexane/CH_2_Cl_2_ by slow solvent evaporation at ambient temperature and atmosphere. CCDC 1988141 contains the complete supplementary crystallographic data file; selected crystallographic data are shown in Table S1. White solid, m.p.: 60–62 °C; ^1^H NMR (600 MHz, 300 K, CDCl_3_): *δ*=8.64 (d, *J*=4.8 Hz, 1H), 7.70 (d, *J*=4.8 Hz, 1H), 4.04 (s, 3H), 4.00 ppm (s, 3H); ^13^C NMR (150 MHz, 300 K, CDCl_3_): *δ*=164.7, 164.2, 150.4, 147.3, 139.7, 127.9, 125.5, 53.2, 53.1(8) ppm; IR (film): $\tilde{\nu }$ =3005, 2957, 1737, 1579, 1543, 1453, 1433, 1389, 1316, 1269, 1199, 1172, 1154, 1115, 1047, 987, 953 cm^−1^; HRMS (ESI): *m/z* calcd for C_9_H_9_O_4_NCl [*M*+H]^+^: 230.0215, found: 230.0215.

**Dimethyl 3‐((4‐methoxybenzyl)amino)pyridine‐2,4‐dicarboxylate (22)**: According to General Procedure A, pyridine **22** (471 mg, 71 %) was obtained from dimethyl 3‐chloropyridine‐2,4‐dicarboxylate **24** (459 mg, 2.0 mmol) after column chromatography (25 g Ultra; 45 mL/min; 100 % cyclohexane (3 CV) followed by a linear gradient (10 CV): 0→30 % ethyl acetate in cyclohexane). The analytical data of pyridine **22** correspond to those reported.^31^ Yellow solid, m.p.: 72–74 °C; ^1^H NMR (600 MHz, 300 K, CDCl_3_): *δ*=8.33 (t, *J*=4.8 Hz, 1H), 8.04 (d, *J*=4.7 Hz, 1H), 7.63 (d, *J*=4.7 Hz, 1H), 7.22−7.20 (m, 2H), 6.88−6.86 (m, 2H), 4.19 (d, *J*=4.7 Hz, 2H), 3.96 (s, 3H), 3.89 (s, 3H), 3.80 ppm (s, 3H); ^13^C NMR (150 MHz, 300 K, CDCl_3_): *δ*=167.6, 167.3, 159.2, 145.4, 135.9, 133.4, 129.9, 129.1, 127.5, 122.7, 114.1, 55.2, 52.8, 52.6, 50.4 ppm; IR (film): $\tilde{\nu }$ =3328, 2998, 2952, 2838, 1722, 1696, 1612, 1584, 1567, 1512, 1438, 1294, 1247, 1192, 1161, 1127, 1106, 1078, 1033, 1000 cm^−1^; HRMS (ESI): *m/z* calcd for C_17_H_19_O_5_N_2_ [*M*+H]^+^: 331.1289, found: 331.1285.

**3‐((4‐Methoxybenzyl)amino)pyridine‐2,4‐dicarboxylic acid (18)**: Pyridine **18** (133 mg, 88 %) was obtained from dimethyl pyridine‐2,4‐dicarboxylate **22** (165 mg, 0.5 mmol) according to General Procedure B. Yellow solid, m.p.: >250 °C (decomposition); ^1^H NMR (600 MHz, 300 K, D_2_O): *δ*=7.93 (d, *J*=5.0 Hz, 1H), 7.43 (d, *J*=5.0 Hz, 1H), 7.30 (d, *J*=8.5 Hz, 2H), 6.96 (d, *J*=8.6 Hz, 2H), 4.35 (s, 2H), 3.82 ppm (s, 3H); ^13^C NMR (150 MHz, 300 K, D_2_O): *δ*=174.6, 172.7, 158.0, 142.0, 141.2, 136.9, 136.4, 131.9, 129.4, 124.4, 114.1, 55.3, 49.4 ppm; IR (film): $\tilde{\nu }$ =3262, 2960, 2926, 1609, 1512, 1465, 1388, 1301, 1246, 1177, 1107, 1030 cm^−1^; HRMS (ESI): *m/z* calcd for C_15_H_13_O_5_N_2_ [*M*−H]^−^: 301.0830, found: 301.0826.

**Dimethyl 3‐(benzylamino)pyridine‐2,4‐dicarboxylate (34)**: According to General Procedure A, pyridine **34** (191 mg, 32 %) was obtained from dimethyl 3‐chloropyridine‐2,4‐dicarboxylate **24** (459 mg, 2.0 mmol) after column chromatography (25 g Ultra; 50 mL/min; 100 % cyclohexane (3 CV) followed by a linear gradient (10 CV): 0→20 % ethyl acetate in cyclohexane). Single‐crystals suitable for X‐ray diffraction analysis were obtained from a concentrated solution of analytically pure **34** in cyclohexane/CH_2_Cl_2_ by slow solvent evaporation at ambient temperature and atmosphere. CCDC 1988142 contains the complete supplementary crystallographic data file; the molecular structure is shown in Figure S1 and selected crystallographic data in Table S1. Yellow solid, m.p.: 71–72 °C; ^1^H NMR (600 MHz, 300 K, CDCl_3_): *δ*=8.45 (brt, *J*=4.2 Hz, 1H), 8.06 (d, *J*=4.6 Hz, 1H), 7.64 (d, *J*=4.7 Hz, 1H), 7.37–7.35 (m, 2H), 7.32−7.29 (m, 3H), 4.29 (d, *J*=4.9 Hz, 2H), 3.97 (s, 3H), 3.89 ppm (s, 3H); ^13^C NMR (150 MHz, 300 K, CDCl_3_): *δ*=167.7, 167.3, 145.5, 137.9, 136.1, 133.5, 128.8, 127.8, 127.7, 127.6, 122.9, 52.8, 52.6, 50.9 ppm; IR (film): $\tilde{\nu }$ =3327, 3030, 2952, 1721, 1697, 1585, 1568, 1498, 1437, 1344, 1293, 1241, 1192, 1162, 1127, 1108, 1000 cm^−1^; HRMS (ESI): *m/z* calcd for C_16_H_17_O_4_N_2_ [*M*+H]^+^: 301.1183, found: 301.1184.

**3‐(Benzylamino)pyridine‐2,4‐dicarboxylic acid (25)**: Pyridine **25** (86 mg, 63 %) was obtained from dimethyl pyridine‐2,4‐dicarboxylate **34** (150 mg, 0.5 mmol) according to General Procedure B. Yellow solid, m.p.: >240 °C (decomposition); ^1^H NMR (600 MHz, 300 K, D_2_O): *δ*=7.95 (d, *J*=5.5 Hz, 1H), 7.67 (d, *J*=5.5 Hz, 1H), 7.43–7.39 (m, 4H), 7.37–7.35 (m, 1H), 4.58 ppm (s, 2H); ^13^C NMR (150 MHz, 300 K, D_2_O): *δ*=172.9, 166.3, 144.0, 141.1, 138.3, 130.9 (br), 129.0 (br), 128.8, 127.7, 127.6(5), 126.5, 48.5 ppm; IR (film): $\tilde{\nu }$ =3182, 3031, 2980, 1618, 1526, 1498, 1453, 1386, 1291, 1244, 1207, 1104 cm^−1^; HRMS (ESI): *m/z* calcd for C_14_H_13_O_4_N_2_ [*M*+H]^+^: 273.0870, found: 273.0869.

**Dimethyl 3‐(phenethylamino)pyridine‐2,4‐dicarboxylate (35)**: According to General Procedure A, pyridine **35** (414 mg, 66 %) was obtained from dimethyl 3‐chloropyridine‐2,4‐dicarboxylate **24** (459 mg, 2.0 mmol) after column chromatography (25 g Ultra; 50 mL/min; 100 % cyclohexane (3 CV) followed by a linear gradient (10 CV): 0→20 % ethyl acetate in cyclohexane). Clear yellow oil; ^1^H NMR (600 MHz, 300 K, CDCl_3_): *δ*=8.07 (brs, 1H), 8.02 (d, *J*=4.7 Hz, 1H), 7.60 (d, *J*=4.7 Hz, 1H), 7.32–7.30 (m, 2H), 7.25–7.21 (m, 3H), 3.98 (s, 3H), 3.91 (s, 3H), 3.34–3.31 (m, 2H), 2.95 ppm (t, *J*=7.2 Hz, 2H); ^13^C NMR (150 MHz, 300 K, CDCl_3_): *δ*=167.6, 167.4, 145.7, 138.2, 136.0, 133.7, 128.7, 128.6, 127.4, 126.6, 122.9, 52.8, 52.6, 48.2, 36.6 ppm; IR (film): $\tilde{\nu }$ =3326, 3028, 2951, 1725, 1698, 1570, 1499, 1438, 1349, 1293, 1243, 1193, 1160, 1126, 1108, 1089, 996 cm^−1^; HRMS (ESI): *m/z* calcd for C_17_H_19_O_4_N_2_ [*M*+H]^+^: 315.1339, found: 315.1336.

**3‐(Phenethylamino)pyridine‐2,4‐dicarboxylic acid (26)**: Pyridine **26** (101 mg, 71 %) was obtained from dimethyl pyridine‐2,4‐dicarboxylate **35** (157 mg, 0.5 mmol) according to General Procedure B. Yellow solid, m.p.: >220 °C (decomposition); ^1^H NMR (600 MHz, 300 K, D_2_O): *δ*=7.85 (d, *J*=5.5 Hz, 1H), 7.63 (d, *J*=5.5 Hz, 1H), 7.37–7.34 (m, 2H), 7.31–7.30 (m, 2H), 7.28–7.26 (m, 1H), 3.66 (t, *J*=6.5 Hz, 2H), 2.97 ppm (t, *J*=6.5 Hz, 2H); ^13^C NMR (150 MHz, 300 K, D_2_O): *δ*=173.1, 165.6, 144.8, 141.0, 139.0, 129.0, 128.9(8), 128.7, 127.4 (br), 126.6, 126.4 (br), 45.9 (br), 35.6 (br) ppm; IR (film): $\tilde{\nu }$ =3194, 3028, 2924, 1617, 1531, 1497, 1455, 1387, 1293, 1244, 1210, 1107 cm^−1^; HRMS (ESI): *m/z* calcd for C_15_H_13_O_4_N_2_ [*M*−H]^−^: 285.0881, found: 285.0879.

**Dimethyl 3‐((3‐phenylpropyl)amino)pyridine‐2,4‐dicarboxylate (36)**: According to General Procedure A, pyridine **36** (379 mg, 58 %) was obtained from dimethyl 3‐chloropyridine‐2,4‐dicarboxylate **24** (459 mg, 2.0 mmol) after column chromatography (25 g Ultra; 50 mL/min; 100 % cyclohexane (3 CV) followed by a linear gradient (10 CV): 0→20 % ethyl acetate in cyclohexane). Clear yellow oil; ^1^H NMR (600 MHz, 300 K, CDCl_3_): *δ*=8.11 (brt, *J*=4.7 Hz, 1H), 8.02 (d, *J*=4.7 Hz, 1H), 7.60 (d, *J*=4.6 Hz, 1H), 7.30–7.27 (m, 2H), 7.22–7.19 (m, 1H), 7.17–7.15 (m, 2H), 3.97 (s, 3H), 3.89 (s, 3H), 3.06–3.03 (m, 2H), 2.72 (t, *J*=7.6 Hz, 2H), 2.02–1.96 ppm (m, 2H); ^13^C NMR (150 MHz, 300 K, CDCl_3_): *δ*=167.7, 167.5, 145.9, 141.0, 135.7, 133.3, 128.4, 128.3(7), 127.4, 126.1, 122.6, 52.8, 52.5, 46.2, 33.1, 32.0 ppm; IR (film): $\tilde{\nu }$ =3329, 3027, 2950, 2865, 1727, 1696, 1586, 1571, 1499, 1438, 1294, 1243, 1195, 1157, 1126, 1108, 997 cm^−1^; HRMS (ESI): *m/z* calcd for C_18_H_21_O_4_N_2_ [*M*+H]^+^: 329.1496, found: 329.1493.

**3‐((3‐Phenylpropyl)amino)pyridine‐2,4‐dicarboxylic acid (27)**: Pyridine **27** (91 mg, 61 %) was obtained from dimethyl pyridine‐2,4‐dicarboxylate **36** (164 mg, 0.5 mmol) according to General Procedure B. Yellow solid, m.p.: >220 °C (decomposition); ^1^H NMR (600 MHz, 300 K, D_2_O): *δ*=7.84 (d, *J*=5.5 Hz, 1H), 7.61 (d, *J*=5.5 Hz, 1H), 7.28–7.25 (m, 2H), 7.22–7.21 (m, 2H), 7.19–7.17 (m, 1H), 3.32 (t, *J*=6.3 Hz, 2H), 2.73 (t, *J*=7.3 Hz, 2H), 1.99 ppm (app. quint., *J*=6.8 Hz, 2H); ^13^C NMR (150 MHz, 300 K, D_2_O): *δ*=173.1, 166.0, 144.6, 141.7, 140.3, 128.7 (br), 128.5 (2 C), 127.2 (br), 126.4, 125.8, 44.2, 32.6, 30.8 ppm; IR (film): $\tilde{\nu }$ =3194, 2980, 2935, 2864, 1618, 1603, 1531, 1497, 1467, 1454, 1385, 1291, 1244, 1207, 1175, 1104, 1030 cm^−1^; HRMS (ESI): *m/z* calcd for C_16_H_15_O_4_N_2_ [*M*−H]^−^: 299.1037, found: 299.1034.

**Dimethyl 3‐((3‐phenylbutyl)amino)pyridine‐2,4‐dicarboxylate (37)**: According to General Procedure A, pyridine **37** (398 mg, 58 %) was obtained from dimethyl 3‐chloropyridine‐2,4‐dicarboxylate **24** (459 mg, 2.0 mmol) after column chromatography (25 g Ultra; 50 mL/min; 100 % cyclohexane (3 CV) followed by a linear gradient (10 CV): 0→15 % ethyl acetate in cyclohexane). Clear yellow oil; ^1^H NMR (600 MHz, 300 K, CDCl_3_): *δ*=8.06 (brs, 1H), 8.01 (d, *J*=4.7 Hz, 1H), 7.60 (d, *J*=4.6 Hz, 1H), 7.30–7.28 (m, 2H), 7.21–7.17 (m, 3H), 3.99 (s, 3H), 3.93 (s, 3H), 3.06–3.04 (m, 2H), 2.66 (t, *J*=7.2 Hz, 2H), 1.77–1.67 ppm (m, 4H); ^13^C NMR (150 MHz, 300 K, CDCl_3_): *δ*=167.8, 167.5, 145.9, 141.8, 135.6, 133.2, 128.3 (2 C), 127.4, 125.9, 122.5, 52.8, 52.5, 46.7, 35.4, 29.9, 28.5 ppm; IR (film): $\tilde{\nu }$ =3330, 3027, 2948, 2859, 1727, 1696, 1585, 1571, 1504, 1438, 1294, 1243, 1193, 1153, 1126, 1109, 999 cm^−1^; HRMS (ESI): *m/z* calcd for C_19_H_23_O_4_N_2_ [*M*+H]^+^: 343.1652, found: 343.1656.

**3‐((4‐Phenylbutyl)amino)pyridine‐2,4‐dicarboxylic acid (28)**: Pyridine **28** (99 mg, 63 %) was obtained from dimethyl pyridine‐2,4‐dicarboxylate **37** (171 mg, 0.5 mmol) according to General Procedure B. Yellow solid, m.p.: >210 °C (decomposition); ^1^H NMR (600 MHz, 300 K, D_2_O): *δ*=7.88 (d, *J*=5.5 Hz, 1H), 7.61 (d, *J*=5.5 Hz, 1H), 7.32–7.30 (m, 2H), 7.26–7.25 (m, 2H), 7.21–7.19 (m, 1H), 3.33 (t, *J*=6.5 Hz, 2H), 2.65 (t, *J*=7.2 Hz, 2H), 1.77–1.72 (m, 2H), 1.68–1.64 ppm (m, 2H); ^13^C NMR (150 MHz, 300 K, D_2_O): *δ*=173.1, 166.1, 144.3 (br), 142.9, 140.8, 129.5 (br), 128.5, 128.4(9), 128.1 (br), 126.3, 125.7, 44.2, 34.3, 28.3, 27.5 ppm; IR (film): $\tilde{\nu }$ =3182, 2980, 2931, 2858, 1619, 1532, 1453, 1384, 1290, 1244, 1205, 1105 cm^−1^; HRMS (ESI): *m/z* calcd for C_17_H_17_O_4_N_2_ [*M*−H]^−^: 313.1194, found: 313.1191.

***rac*** **‐Dimethyl 3‐((1‐phenylethyl)amino)pyridine‐2,4‐dicarboxylate (38)**: According to General Procedure A, pyridine **38** (304 mg, 48 %) was obtained from dimethyl 3‐chloropyridine‐2,4‐dicarboxylate **24** (459 mg, 2.0 mmol) after column chromatography (25 g Ultra; 50 mL/min; 100 % cyclohexane (3 CV) followed by a linear gradient (10 CV): 0→30 % ethyl acetate in cyclohexane). Yellow solid, m.p.: 95–96 °C; ^1^H NMR (600 MHz, 300 K, CDCl_3_): *δ*=8.67 (brd, *J*=6.6 Hz, 1H), 7.99 (d, *J*=4.7 Hz, 1H), 7.50 (d, *J*=4.6 Hz, 1H), 7.27–7.24 (m, 2H), 7.21–7.19 (m, 1H), 7.13 (d, *J*=7.4 Hz, 2H), 4.74 (app. quint., *J*=6.7 Hz, 1H), 3.94 (s, 3H), 3.78 (s, 3H), 1.60 ppm (d, *J*=6.7 Hz, 3H); ^13^C NMR (150 MHz, 300 K, CDCl_3_): *δ*=167.8, 167.2, 144.7, 143.0, 136.3, 134.1, 128.6, 127.5, 127.3, 126.2, 124.3, 55.1, 52.8, 52.5, 25.6 ppm; IR (film): $\tilde{\nu }$ =3322, 3029, 2952, 1721, 1697, 1589, 1568, 1509, 1437, 1375, 1292, 1243, 1193, 1166, 1126, 999 cm^−1^; HRMS (ESI): *m/z* calcd for C_17_H_19_O_4_N_2_ [*M*+H]^+^: 315.1339, found: 315.1341.

***rac*** **‐3‐((1‐Phenylethyl)amino)pyridine‐2,4‐dicarboxylic acid (29)**: Pyridine **29** (103 mg, 72 %) was obtained from dimethyl pyridine‐2,4‐dicarboxylate **38** (157 mg, 0.5 mmol) according to General Procedure B. Yellow solid, m.p.: >270 °C (decomposition); ^1^H NMR (600 MHz, 300 K, D_2_O): *δ*=7.92 (d, *J*=5.4 Hz, 1H), 7.55 (d, *J*=5.4 Hz, 1H), 7.36–7.31 (m, 4H), 7.29–7.26 (m, 1H), 4.97 (q, *J*=6.7 Hz, 1H), 1.56 ppm (d, *J*=6.7 Hz, 3H); ^13^C NMR (150 MHz, 300 K, D_2_O): *δ*=172.7, 167.2 (br), 144.1, 143.2, 141.7, 133.5 (br), 130.9 (br), 128.7, 127.3, 126.3, 126.2, 54.6, 23.8 ppm; IR (film): $\tilde{\nu }$ =3182, 3030, 2927, 1612, 1519, 1452, 1374, 1288, 1243, 1211, 1125, 1102, 1019 cm^−1^; HRMS (ESI): *m/z* calcd for C_15_H_13_O_4_N_2_ [*M*−H]^−^: 285.0881, found: 285.0877.

***rac*** **‐Dimethyl 3‐((1‐phenylpropyl)amino)pyridine‐2,4‐dicarboxylate (39)**: According to General Procedure A, pyridine **39** (268 mg, 41 %) was obtained from dimethyl 3‐chloropyridine‐2,4‐dicarboxylate **24** (459 mg, 2.0 mmol) after column chromatography (25 g Ultra; 50 mL/min; 100 % cyclohexane (3 CV) followed by a linear gradient (12 CV): 0→20 % ethyl acetate in cyclohexane). Yellow solid, m.p.: 83–85 °C; ^1^H NMR (600 MHz, 300 K, CDCl_3_): *δ*=8.77 (brd, *J*=6.7 Hz, 1H), 7.97 (d, *J*=4.6 Hz, 1H), 7.49 (d, *J*=4.6 Hz, 1H), 7.26–7.24 (m, 2H), 7.21–7.19 (m, 1H), 7.09 (d, *J*=7.7 Hz, 2H), 4.52 (q, *J*=6.7 Hz, 1H), 3.94 (s, 3H), 3.77 (s, 3H), 2.00–1.93 (m, 1H), 1.90–1.83 (m, 1H), 1.00 ppm (t, *J*=7.4 Hz, 3H); ^13^C NMR (150 MHz, 300 K, CDCl_3_): *δ*=167.8, 167.2, 145.1, 141.4, 136.1, 133.9, 128.4, 127.5, 127.2, 126.9, 124.2, 61.1, 52.7, 52.4, 32.6, 10.6 ppm; IR (film): $\tilde{\nu }$ =3307, 3002, 2948, 2874, 1713, 1678, 1585, 1503, 1455, 1431, 1370, 1285, 1239, 1190, 1165, 1121, 1103, 1043, 1000, 962 cm^−1^; HRMS (ESI): *m/z* calcd for C_18_H_21_O_4_N_2_ [*M*+H]^+^: 329.1496, found: 329.1495.

***rac*** **‐3‐((1‐Phenylpropyl)amino)pyridine‐2,4‐dicarboxylic acid (30)**: Pyridine **30** (66 mg, 88 %) was obtained from dimethyl pyridine‐2,4‐dicarboxylate **39** (82 mg, 0.25 mmol) according to General Procedure B. Yellow solid, m.p.: >240 °C (decomposition); ^1^H NMR (600 MHz, 300 K, D_2_O): *δ*=7.90 (d, *J*=5.5 Hz, 1H), 7.59 (d, *J*=5.6 Hz, 1H), 7.37–7.35 (m, 2H), 7.31–7.27 (m, 3H), 4.83 (t, *J*=6.7 Hz, 1H), 1.96–1.86 (m, 2H), 1.56 ppm (t, *J*=7.4 Hz, 3H); ^13^C NMR (150 MHz, 300 K, D_2_O): *δ*=172.3, 165.8, 144.4, 142.5, 142.4, 130.3, 128.6, 128.5(7), 127.3, 127.0, 126.7, 60.1, 31.3, 9.9 ppm; IR (film): $\tilde{\nu }$ =3196, 3065, 2965, 2933, 1621, 1596, 1523, 1494, 1455, 1382, 1286, 1242, 1208, 1103 cm^−1^; HRMS (ESI): *m/z* calcd for C_16_H_15_O_4_N_2_ [*M*−H]^−^: 299.1037, found: 299.1033.

**Dimethyl 3‐((4‐(trifluoromethyl)benzyl)amino)pyridine‐2,4‐dicarboxylate (40)**: According to General Procedure A, pyridine **40** (307 mg, 42 %) was obtained from dimethyl 3‐chloropyridine‐2,4‐dicarboxylate **24** (459 mg, 2.0 mmol) after column chromatography (25 g Ultra; 50 mL/min; 100 % cyclohexane (3 CV) followed by a linear gradient (10 CV): 0→25 % ethyl acetate in cyclohexane). Clear yellow oil; ^1^H NMR (600 MHz, 300 K, CDCl_3_): *δ*=8.51 (brt, *J*=4.7 Hz, 1H), 8.10 (d, *J*=4.7 Hz, 1H), 7.67 (d, *J*=4.6 Hz, 1H), 7.62 (d, *J*=8.2 Hz, 2H), 7.43 (d, *J*=8.0 Hz, 2H), 4.37 (d, *J*=5.3 Hz, 2H), 3.97 (s, 3H), 3.88 ppm (s, 3H); ^19^F NMR (565 MHz, 300 K, CDCl_3_): *δ*=−62.6 ppm (s, 3F); ^13^C NMR (150 MHz, 300 K, CDCl_3_): *δ*=167.7, 167.3, 145.4, 142.0, 136.7, 133.8, 130.1 (q, *J*=32.4 Hz), 127.9, 127.7, 125.8 (q, *J*=3.6 Hz), 124.0 (q, *J*=272.1 Hz), 123.1, 52.9, 52.7, 50.4 ppm; IR (film): $\tilde{\nu }$ =3325, 2955, 1723, 1698, 1620, 1587, 1570, 1505, 1439, 1418, 1325, 1293, 1242, 1194, 1162, 1122, 1067, 1018, 957 cm^−1^; HRMS (ESI): *m/z* calcd for C_17_H_16_O_4_N_2_F_3_ [*M*+H]^+^: 369.1057, found: 369.1055.

**3‐((4‐(Trifluoromethyl)benzyl)amino)pyridine‐2,4‐dicarboxylic acid (31)**: Pyridine **31** (121 mg, 71 %) was obtained from dimethyl pyridine‐2,4‐dicarboxylate **40** (184 mg, 0.5 mmol) according to General Procedure B. Yellow solid, m.p.: >220 °C (decomposition); ^1^H NMR (600 MHz, 300 K, D_2_O): *δ*=7.94 (dd, *J*=5.5, 0.5 Hz, 1H), 7.69 (d, *J*=8.2 Hz, 2H), 7.62 (d, *J*=5.5 Hz, 1H), 7.52 (d, *J*=8.1 Hz, 2H), 4.66 ppm (s, 2H); ^19^F NMR (565 MHz, 300 K, D_2_O): *δ*=−62.2 ppm (s, 3F); ^13^C NMR (150 MHz, 300 K, D_2_O): *δ*=173.0, 166.9 (br), 143.7, 142.9, 140.7, 132.1 (br), 129.7 (br), 128.8 (q, *J*=32.0 Hz), 127.9, 126.2, 125.5 (q, *J*=3.7 Hz), 124.2 (q, *J*=271.4 Hz), 48.0 ppm; IR (film): $\tilde{\nu }$ =3264, 2926, 2852, 1601, 1532, 1450, 1386, 1325, 1294, 1164, 1114, 1067, 1018 cm^−1^; HRMS (ESI): *m/z* calcd for C_15_H_10_O_4_N_2_F_3_ [*M*−H]^−^: 339.0598, found: 339.0595.

**Dimethyl 3‐((4‐chlorophenethyl)amino)pyridine‐2,4‐dicarboxylate (41)**: According to General Procedure A, pyridine **41** (400 mg, 57 %) was obtained from dimethyl 3‐chloropyridine‐2,4‐dicarboxylate **24** (459 mg, 2.0 mmol) after column chromatography (25 g Ultra; 50 mL/min; 100 % cyclohexane (3 CV) followed by a linear gradient (10 CV): 0→20 % ethyl acetate in cyclohexane). Clear yellow oil; ^1^H NMR (600 MHz, 300 K, CDCl_3_): *δ*=8.06 (brt, *J*=4.7 Hz, 1H), 8.04 (d, *J*=4.7 Hz, 1H), 7.61 (d, *J*=4.7 Hz, 1H), 7.29–7.27 (m, 2H), 7.16–7.15 (m, 2H), 3.98 (s, 3H), 3.92 (s, 3H), 3.32–3.29 (m, 2H), 2.92 ppm (t, *J*=7.1 Hz, 2H); ^13^C NMR (150 MHz, 300 K, CDCl_3_): *δ*=167.6, 167.3, 145.6, 136.7, 136.1, 133.7, 132.5, 130.0, 128.7, 127.4, 122.9, 52.8, 52.6, 48.0, 35.9 ppm; IR (film): $\tilde{\nu }$ =3325, 2995, 2951, 2875, 1725, 1698, 1585, 1570, 1493, 1438, 1349, 1293, 1243, 1195, 1159, 1126, 1109, 1091, 1015, 996 cm^−1^; HRMS (ESI): *m/z* calcd for C_17_H_18_O_4_N_2_Cl [*M*+H]^+^: 349.0950, found: 349.0949.

**3‐((4‐Chlorophenethyl)amino)pyridine‐2,4‐dicarboxylic acid (32)**: Pyridine **32** (111 mg, 69 %) was obtained from dimethyl pyridine‐2,4‐dicarboxylate **41** (174 mg, 0.5 mmol) according to General Procedure B. Yellow solid, m.p.: >230 °C (decomposition); ^1^H NMR (600 MHz, 300 K, D_2_O): *δ*=7.86 (d, *J*=5.5 Hz, 1H), 7.62 (d, *J*=5.5 Hz, 1H), 7.33–7.31 (m, 2H), 7.25–7.24 (m, 2H), 3.64 (t, *J*=6.5 Hz, 2H), 2.94 ppm (t, *J*=6.5 Hz, 2H); ^13^C NMR (150 MHz, 300 K, D_2_O): *δ*=173.1, 165.7, 144.7, 141.1, 137.6, 131.6, 130.5, 129.4 (br), 128.4, 127.7 (br), 126.3 (br), 45.8 (br), 35.1 (br) ppm; IR (film): $\tilde{\nu }$ =3251, 2934, 2854, 1602, 1533, 1493, 1449, 1387, 1325, 1298, 1245, 1164, 1111, 1067, 1017 cm^−1^; HRMS (ESI): *m/z* calcd for C_15_H_12_O_4_N_2_Cl [*M*−H]^−^: 319.0491, found: 319.0488.

**Dimethyl 3‐((cyclohexylmethyl)amino)pyridine‐2,4‐dicarboxylate (42)**: According to General Procedure A, pyridine **42** (262 mg, 43 %) was obtained from dimethyl 3‐chloropyridine‐2,4‐dicarboxylate **24** (459 mg, 2.0 mmol) after column chromatography (25 g Ultra; 50 mL/min; 100 % cyclohexane (3 CV) followed by a linear gradient (15 CV): 0→16 % ethyl acetate in cyclohexane). Yellow solid, m.p.: 45–47 °C; ^1^H NMR (600 MHz, 300 K, CDCl_3_): *δ*=8.00 (d, *J*=4.6 Hz, 1H), 7.60 (d, *J*=4.6 Hz, 1H), 4.00 (s, 3H), 3.94 (s, 3H), 2.88 (d, *J*=6.5 Hz, 2H), 1.80–1.74 (m, 4H), 1.71–1.69 (m, 1H), 1.62–1.56 (m, 1H), 1.31–1.25 (m, 2H), 1.22–1.14 (m, 1H), 1.03–0.97 ppm (m, 2H), the NH proton was not detected; ^13^C NMR (150 MHz, 300 K, CDCl_3_): *δ*=167.8, 167.6, 146.2, 135.4, 133.0, 127.5, 122.5, 53.6, 52.8, 52.5, 38.6, 30.8, 26.3, 25.8 ppm; IR (film): $\tilde{\nu }$ =3323, 2998, 2924, 2852, 1721, 1697, 1587, 1505, 1437, 1385, 1340, 1291, 1237, 1190, 1161, 1143, 1102, 1072, 997 cm^−1^; HRMS (ESI): *m/z* calcd for C_16_H_23_O_4_N_2_ [*M*+H]^+^: 307.1652, found: 307.1653.

**3‐((Cyclohexylmethyl)amino)pyridine‐2,4‐dicarboxylic acid (33)**: Pyridine **33** (53 mg, 76 %) was obtained from dimethyl pyridine‐2,4‐dicarboxylate **42** (76 mg, 0.25 mmol) according to General Procedure B. Yellow solid, m.p.: >230 °C (decomposition); ^1^H NMR (600 MHz, 300 K, D_2_O): *δ*=7.90 (d, *J*=5.5 Hz, 1H), 7.66 (d, *J*=5.4 Hz, 1H), 3.19 (d, *J*=6.5 Hz, 2H), 1.76–1.71 (m, 4H), 1.66–1.59 (m, 2H), 1.30–1.16 (m, 3H), 1.06–1.00 ppm (m, 2H); ^13^C NMR (150 MHz, 300 K, D_2_O): *δ*=173.2, 166.2, 144.7 (br), 140.7, 129.1 (br), 127.8 (br), 126.4, 51.2, 37.8, 30.1, 26.0, 25.4 ppm; IR (film): $\tilde{\nu }$ =3253, 2924, 2851, 1617, 1533, 1450, 1388, 1292, 1245, 1207, 1109 cm^−1^; HRMS (ESI): *m/z* calcd for C_14_H_19_O_4_N_2_ [*M*+H]^+^: 279.1339, found: 279.1340.

### Biochemical procedures

**Recombinant AspH production and purification**: N‐Terminally His_6_‐tagged human AspH_315‐758_ (His_6_‐AspH_315‐758_) was produced in *Escherichia coli* BL21 (DE3) cells using a pET‐28a(+) vector and purified by Ni^II^‐affinity chromatography (HisTrap HP column, GE Healthcare; 1 mL/min flow rate) and size‐exclusion chromatography (HiLoad 26/60 Superdex 75 pg 300 mL column; 1 mL/min) using an ÄKTA Pure machine (GE Healthcare), as previously reported.^21^, ^23^ AspH was >95 % pure as analysed by SDS‐PAGE and ESI‐MS analysis and had the anticipated mass as reported (*m/z* calcd for His_6_‐AspH_315–758_: 54519 Da, found: 54519 Da).^23^ Purified AspH was stored in 50 mM HEPES buffer (pH 7.5, 150 mM NaCl) at a concentration of 125 μM at −78 °C; fresh aliquots were used for all AspH inhibition assays.

**AspH substrate synthesis**: A synthetic thioether linked cyclic peptide, hFX‐CP_101‐119_ (Figure 2D),^23^ was used as AspH substrate. hFX‐CP_101‐119_ was designed based on 19 EGFD1 amino acid residues of the sequence of human coagulation factor X (hFX amino acids 101–119), which is a reported cellular AspH substrate.^48^ hFX‐CP_101−119_ was prepared with a C‐terminal amide; it was synthesised by an intramolecular thioetherification cyclisation reaction from the corresponding linear peptide (d‐Ala replacing Cys101_hFX_ and Ser replacing Cys112_hFX_) which was obtained by microwave‐assisted solid phase peptide synthesis using the Fmoc‐protection strategy.^21^

**AspH inhibition assays**: AspH inhibition assays were performed at 2OG and Fe^II^ concentrations close to the relevant *K*_m_ values as previously described.^24^ Co‐substrate/cofactor stock solutions (l‐ascorbic acid, LAA: 50 mM in MQ‐grade water; 2‐oxoglutarate, 2OG: 10 mM in MQ‐grade water; ammonium iron(II) sulfate hexahydrate, FAS, (NH_4_)_2_Fe(SO_4_)_2_ ⋅ 6H_2_O: 400 mM in 20 mM HCl diluted to 1 mM in MQ‐grade water) were freshly prepared from commercial solids (Sigma Aldrich) on the day the assays were performed.

Solutions of the 2,4‐PDCA derivatives (100 % DMSO) were dry dispensed across 384‐well polypropylene assay plates (Greiner) in a 3‐fold and 11‐point dilution series (100 μM top concentration) using an ECHO 550 acoustic dispenser (Labcyte). DMSO and 2,4‐PDCA (**1**) were used as negative and positive controls. The DMSO concentration was kept constant at 0.5 % (*v*/*v*) throughout all experiments (using the DMSO backfill option of the acoustic dispenser). Each reaction was performed in technical duplicates in adjacent wells of the assay plates; additionally, assays were performed in two independent duplicates on different days using different DMSO inhibitor solutions.

The Enzyme Mixture (25 μL/well), containing 0.1 μM His_6_‐AspH_315‐758_ in 50 mM HEPES buffer (pH 7.5), was dispensed across the inhibitor‐containing 384‐well assay plates with a multidrop dispenser (ThermoFischer Scientific) at 20 °C under ambient atmosphere. The plates were subsequently centrifuged (1000 rpm using a Heraeus Megafuge 16 centrifuge equipped with a M‐20 rotor, 15 s) and incubated for 15 min. The Substrate Mixture (25 μL/well), containing 2.0 μM hFX‐CP_101‐119_, 200 μM LAA, 6.0 μM 2OG, and 4.0 μM FAS in 50 mM HEPES buffer (pH 7.5), was added using the multidrop dispenser. Note: The multidrop dispenser ensured proper mixing of the enzyme and the substrate mixtures which was essential for assay reproducibility. The plates were centrifuged (1000 rpm using a Heraeus Megafuge 16 centrifuge equipped with a M‐20 rotor, 15 s) and after incubating for 7 min, the enzyme reaction was stopped by addition of 10 % (*v*/*v*) aqueous formic acid (5 μL/well). The plates were centrifuged (1000 rpm using a Heraeus Megafuge 16 centrifuge equipped with a M‐20 rotor, 60 s) and analysed by MS.

MS‐analyses were performed using a RapidFire RF 365 high‐throughput sampling robot (Agilent) attached to an iFunnel Agilent 6550 accurate mass quadrupole time‐of‐flight (Q‐TOF) mass spectrometer operated in the positive ionisation mode. Assay samples were aspirated under vacuum for 0.4 s and loaded onto a C4 solid phase extraction (SPE) cartridge. After loading, the C4 SPE cartridge was washed with 0.1 % (*v*/*v*) aqueous formic acid to remove non‐volatile buffer salts (5 s, 1.5 mL/min). The peptide was eluted from the SPE cartridge with 0.1 % (*v*/*v*) aqueous formic acid in 85/15 (*v*/*v*) acetonitrile/water into the mass spectrometer (5 s, 1.25 mL/min) and the SPE cartridge re‐equilibrated with 0.1 % (*v*/*v*) aqueous formic acid (1 s, 1.25 mL/min). The mass spectrometer parameters were: capillary voltage (4000 V), nozzle voltage (1000 V), fragmentor voltage (365 V), gas temperature (280 °C), gas flow (13 L/min), sheath gas temperature (350 °C), sheath gas flow (12 L/min). The *m/z* +2 charge states of the cyclic peptide (substrate) and the hydroxylated cyclic peptide (product) were used to extract ion chromatogram data, peak areas were integrated using RapidFire Integrator software (Agilent). The data were exported into Microsoft Excel and used to calculate the % conversion of the hydroxylation reaction using the equation: % conversion=100 x (integral product cyclic peptide) / (integral substrate cyclic peptide+integral product cyclic peptide). Normalised dose‐response curves (2,4‐PDCA and DMSO controls) were obtained from the raw data by non‐linear regression (GraphPad Prism 5) and used to determine IC_50_ values. The standard deviation (SD) of two independent IC_50_ determinations (*n*=2) was calculated using GraphPad Prism 5. Z’‐factors and signal‐to‐noise (S/N) ratios were calculated according to the cited literature using Microsoft Excel (Figure S2).^36^

**PHD2, FIH, and KDM4E inhibition assays**: The *in vitro* PHD2,^49^ FIH,^49^ and KDM4E^39^, ^40^ inhibition assays were performed as described in the cited literature using recombinant human enzymes (PHD2_181‐426_,^49^ FIH,^49^ and KDM4E^39^, ^40^ were prepared according to established procedures). Synthetic peptide substrates were used: HIF‐α CODD, amino acids 558–574 for PHD2;^49^ HIF‐α CAD, amino acids 788–822 for FIH;^49^ H3_1‐15_K9me3, histone 3 (H3) amino acids 1–15 with Lys9 of H3 bearing three methyl groups at the *N* ^ϵ^ position, Lys4 of H3 was substituted by an Ala and Lys14 of H3 by an Ile residue,^40^ for KDM4E. All peptides were prepared as C‐terminal amides by GL Biochem (Shanghai) Ltd.), monitoring peptide hydroxylation in the case of PHD2 and FIH (+16 Da mass shift) or peptide demethylation in the case of KDM4E (‐14 Da mass shift) by SPE‐MS.

## Conflict of interest

The authors declare no conflict of interest.

## Supporting information

As a service to our authors and readers, this journal provides supporting information supplied by the authors. Such materials are peer reviewed and may be re‐organized for online delivery, but are not copy‐edited or typeset. Technical support issues arising from supporting information (other than missing files) should be addressed to the authors.

Supplementary
