# Supplementary material for: Synthesis of Novel Pyridine‐Carboxylates as Small‐Molecule Inhibitors of Human Aspartate/Asparagine‐β‐Hydroxylase
Source: ChemMedChem. 2020 May 26;15(13):1139–49. doi: 10.1002/cmdc.202000147 (PMC7383925; doi:10.1002/cmdc.202000147)

ChemMedChem

Supporting Information

Synthesis of Novel Pyridine-Carboxylates as Small-Molecule Inhibitors of Human Aspartate/Asparagine-β-Hydroxylase

Lennart Brewitz, Anthony Tumber, Armin Thalhammer, Eidarus Salah, Kirsten E. Christensen, and Christopher J. Schofield*© 2020 The Authors. Published by Wiley-VCH Verlag GmbH & Co. KGaA. This is an open access article under the terms of the Creative Commons Attribution License, which permits use, distribution and reproduction in any medium, provided the original work is properly cited.

Supporting Figure S1. Crystallographic analysis confirms the structural assignment of the Buchwald-Hartwig reaction product (34). A single crystal of dimethyl 3-benzyl pyridine-2,4-dicarboxylic acid ester (**34**) was obtained; diffraction analysis confirms its structure as assigned by NMR, IR, and MS. Color code: white: hydrogens; gray: carbons; blue: nitrogens; red: oxygens. Selected crystallographic data are shown in Supporting Table S1.

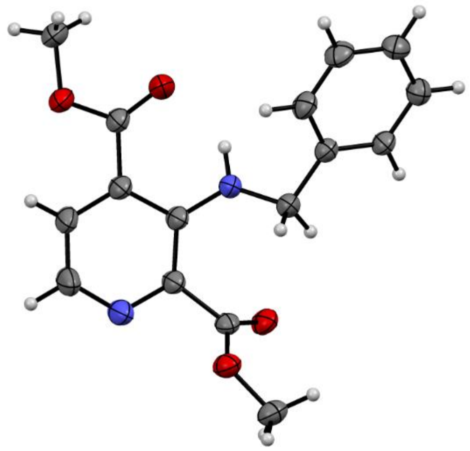

Supporting Table S1. Selected crystallographic data for 2,4-PDCA derivatives **24** and **34**.

Compound	**24**	**34**
CCDC	1988141	1988142
Empirical formula	$C_9H_8ClNO_4$	$C_{16}H_{16}N_2O_4$
Formula weight	229.62	300.31
Crystal system	Monoclinic	Triclinic
Space group	P 2_1/c	P-1
Unit cell dimensions	a = 7.7166(2) Å; α = 90°	a = 7.5225(4) Å; α = 90.005(4)°
	b = 18.3225(4) Å; β = 113.608(4)°	b = 9.2907(5) Å; β = 96.324(4)°
	c = 7.4402(3) Å; γ = 90°	c = 11.3280(6) Å; γ = 111.909(5)°
Volume	963.91(6) $Å^3$	722.95(7) $Å^3$
Z	4	2
Density (calculated)	1.582 mg/m^3	1.379 mg/m^3
F(000)	472	316
Reflections collected	9212	9398
Independent reflections	2003 [R(int) = 0.025]	3012 [R(int) = 0.046]
Data / restraints / parameters	2003 / 0 / 136	3010 / 4 / 204
Goodness-of-fit on F^2	1.0104	1.0013
Final R indices [I > 2σ(I)]	R_1 = 0.0284; wR_2 = 0.0764	R_1 = 0.0497; wR_2 = 0.1315
R indices (all data)	R_1 = 0.0295; wR_2 = 0.0777	R_1 = 0.0610; wR_2 = 0.1445

Supporting Figure S2. Robustness of the AspH inhibition assays. Z'-factors (circles in S2a) and signal-to-noise ratios (S/N, squares in S2b) for all the AspH inhibition assay plates analysed in the described work to determine IC_{50}-values (16 compounds per plate including DMSO and 2,4-PDCA controls; technical duplicates were in adjacent wells). The Z'-factors >0.5 (grey line) indicate a stable and robust assay.[1] Assays were performed as described in the Experimental Section.

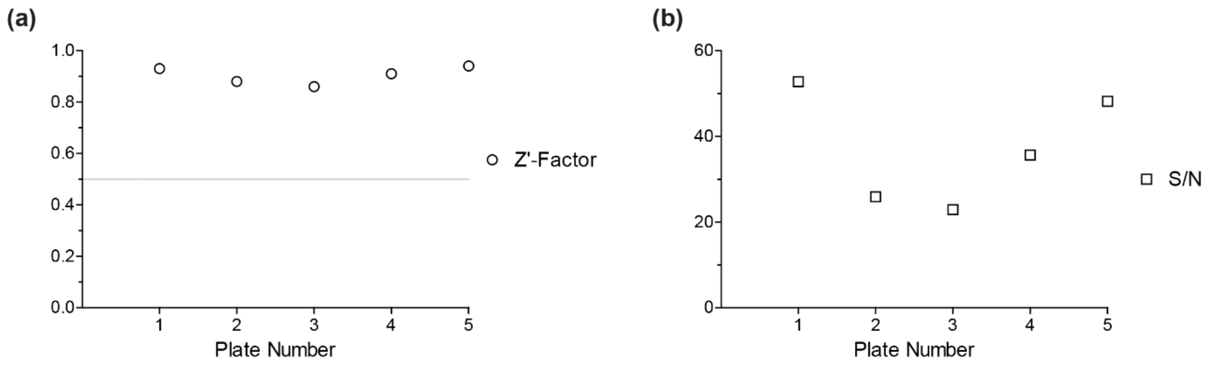

Supporting References

[1] J.-H. Zhang, T. D. Y. Chung, K. R. Oldenburg, *J. Biomol. Screen.* **1999**, *4*, 67-73.

^{1}H and ^{13}C NMR spectra of novel compounds prepared in this study.

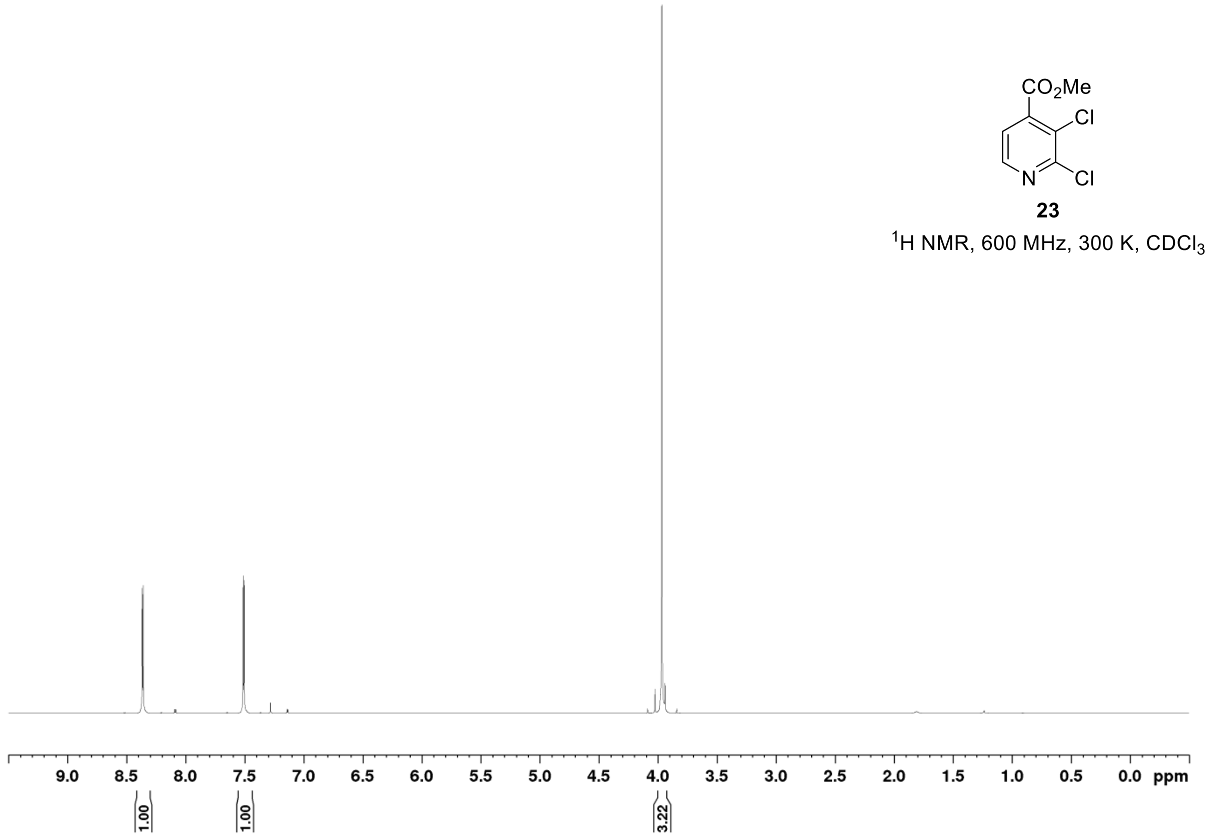

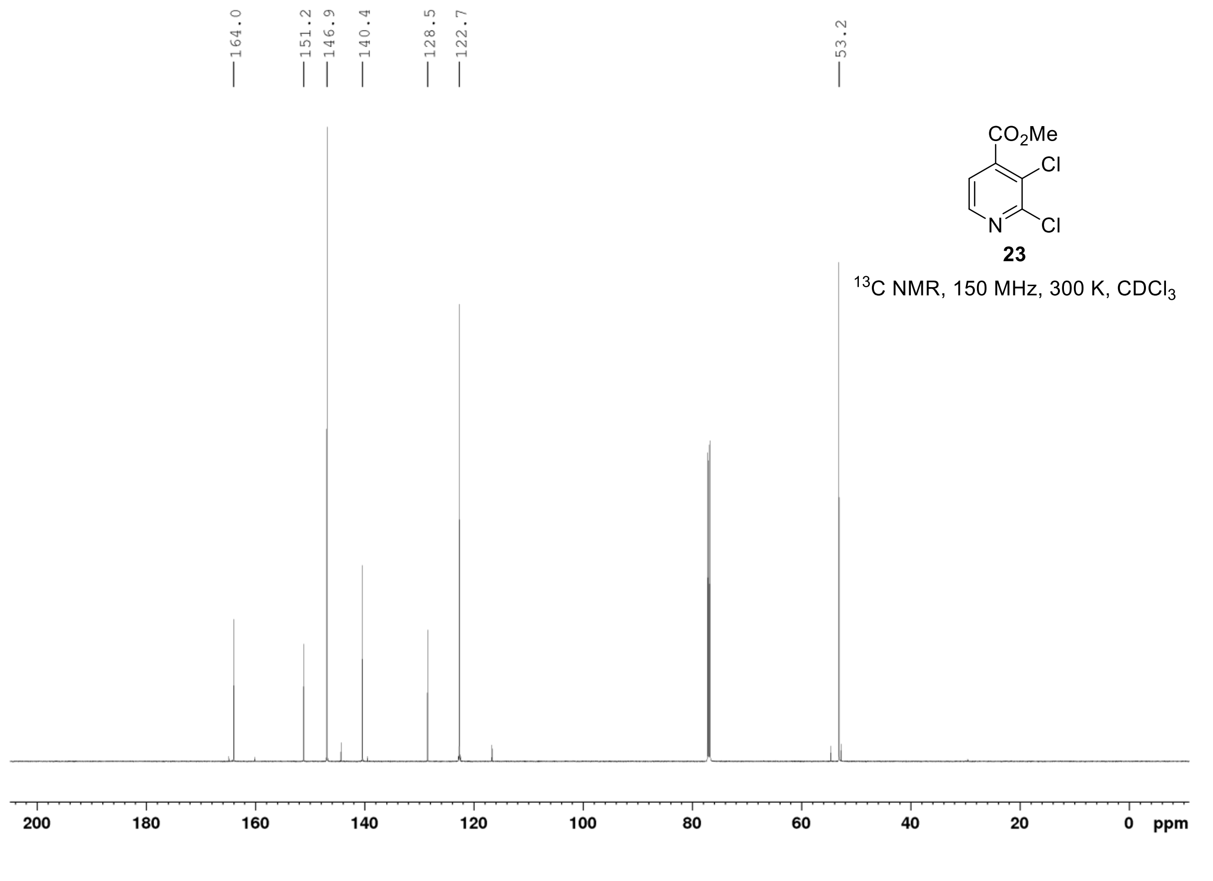

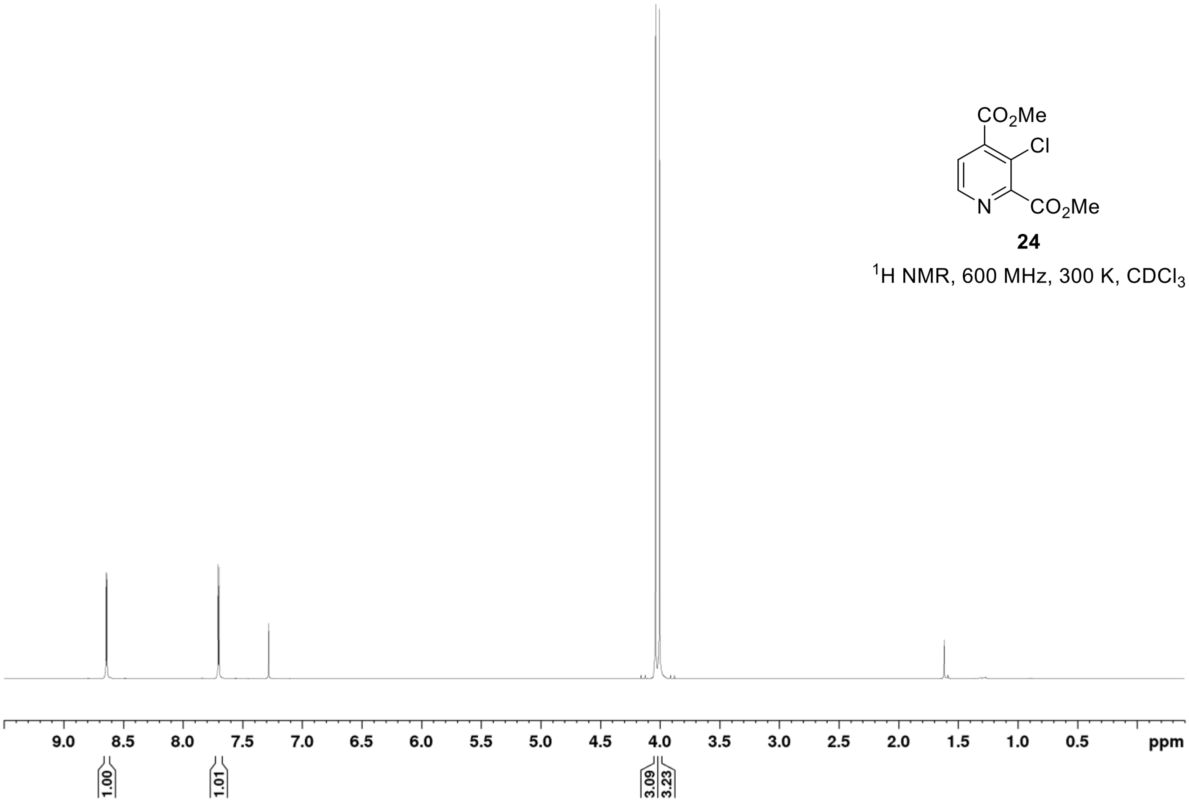

CO2Me
Cl
N
CO2Me
24
1H NMR, 600 MHz, 300 K, CDCl3
9.0 8.5 8.0 7.5 7.0 6.5 6.0 5.5 5.0 4.5 4.0 3.5 3.0 2.5 2.0 1.5 1.0 0.5 ppm
1.00
1.01
3.09
3.23

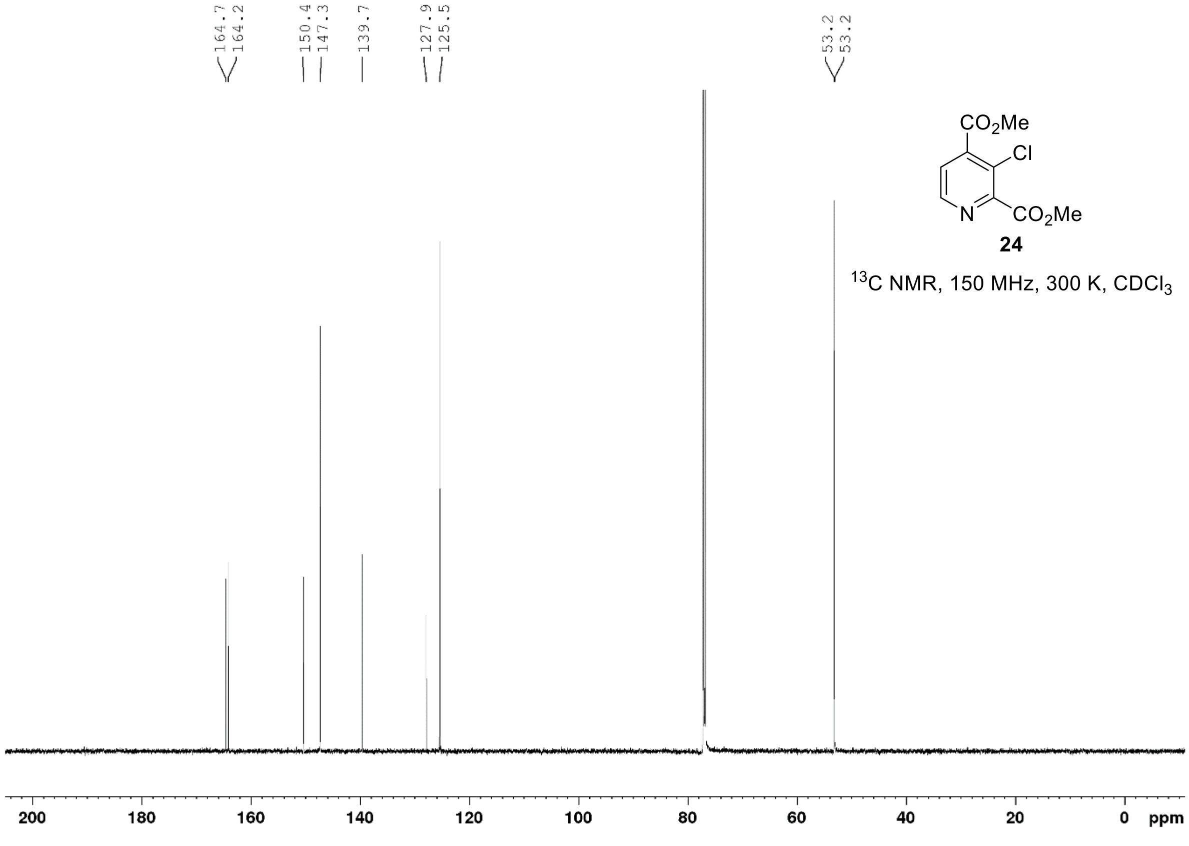

164.7
164.2
150.4
147.3
139.7
127.9
125.5
53.2
53.2
CO2Me
Cl
N
CO2Me
24
13C NMR, 150 MHz, 300 K, CDCl3
200 180 160 140 120 100 80 60 40 20 0 ppm

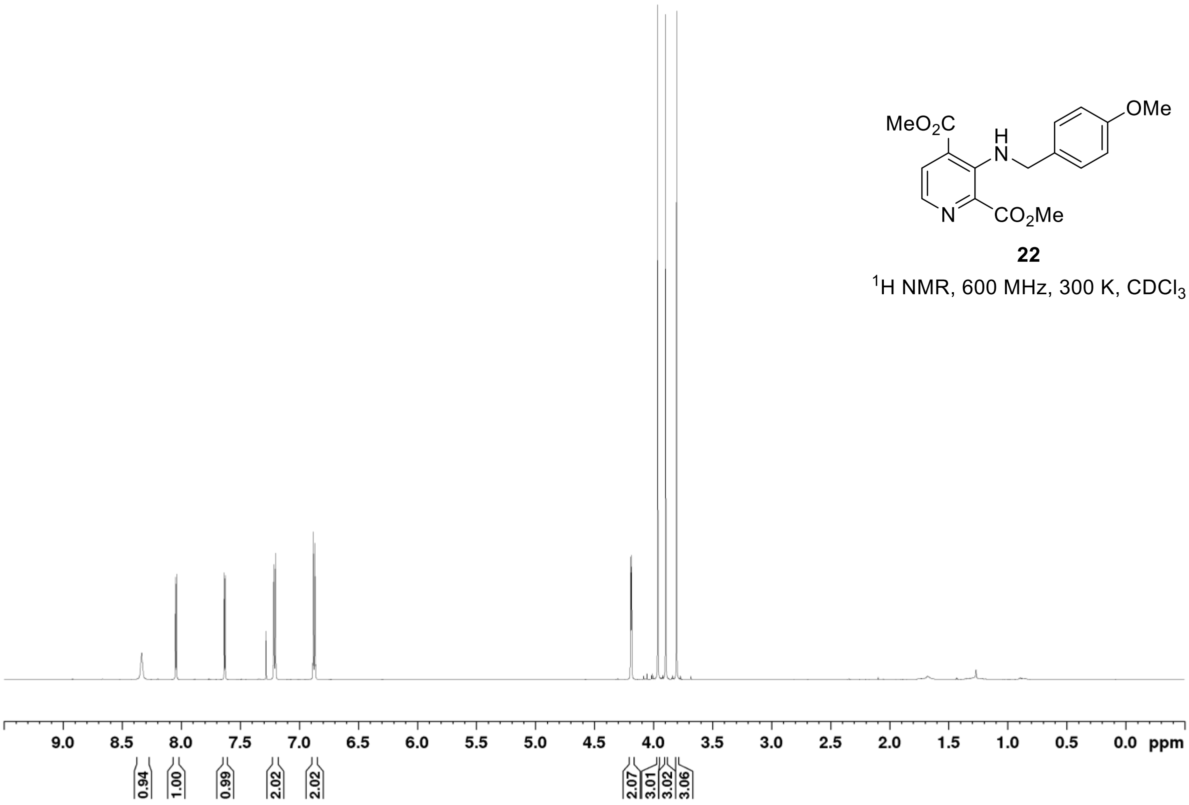
MeO$_2$C
H
N
OMe
N
CO$_2$Me
22
^{1}H NMR, 600 MHz, 300 K, CDCl$_3$
9.0 8.5 8.0 7.5 7.0 6.5 6.0 5.5 5.0 4.5 4.0 3.5 3.0 2.5 2.0 1.5 1.0 0.5 0.0 ppm
0.94 1.00 0.99 2.02 2.02 2.07 3.01 3.02 3.06

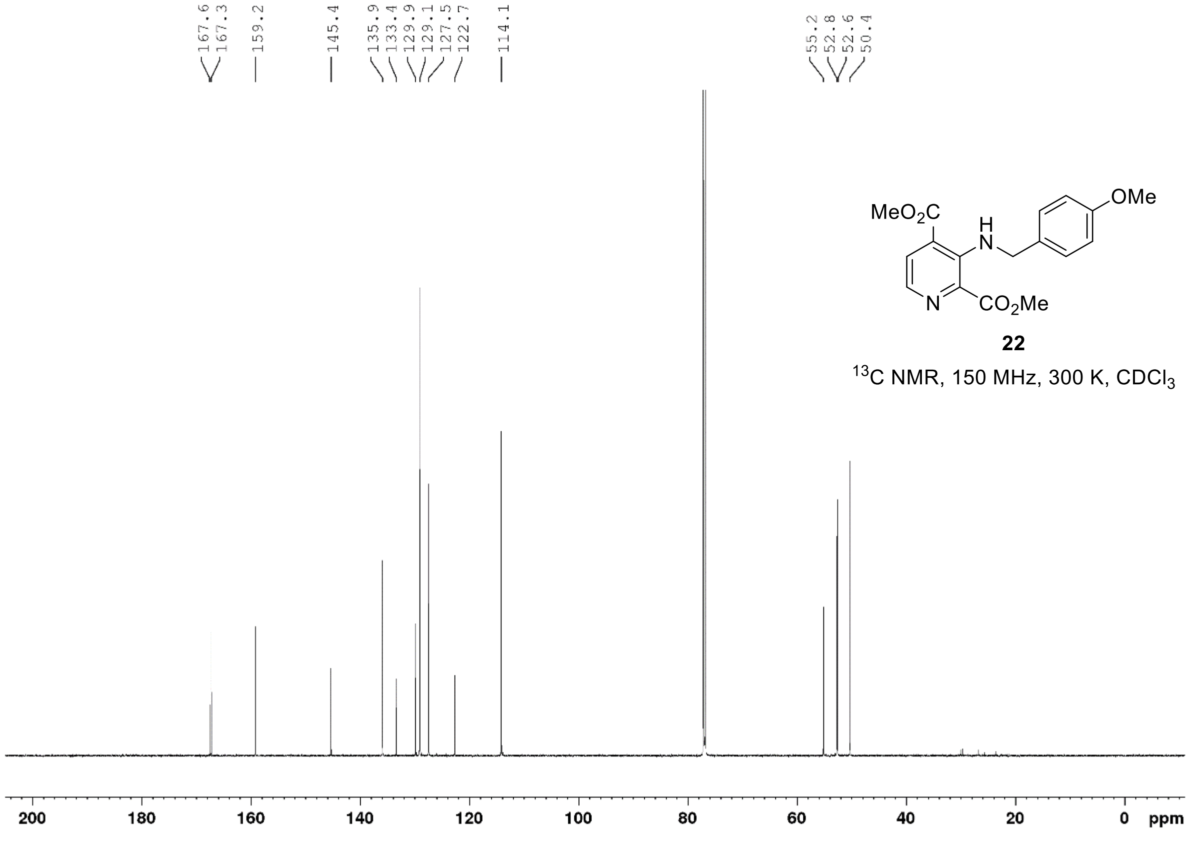
167.6
167.3
159.2
145.4
135.9
133.4
129.9
129.1
127.5
122.7
114.1
55.2
52.8
52.6
50.4
MeO$_2$C
H
N
OMe
N
CO$_2$Me
22
^{13}C NMR, 150 MHz, 300 K, CDCl$_3$
200 180 160 140 120 100 80 60 40 20 0 ppm

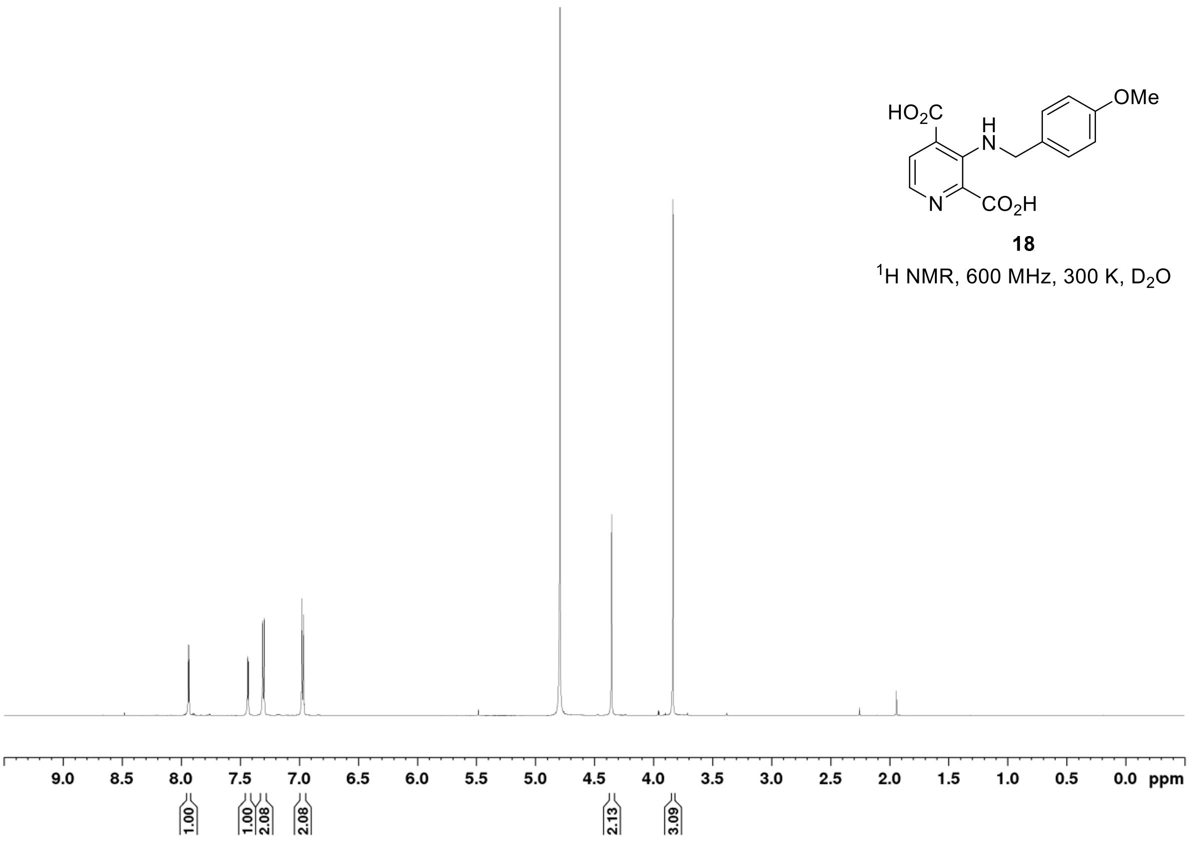

18

^{1}H NMR, 600 MHz, 300 K, D_2O

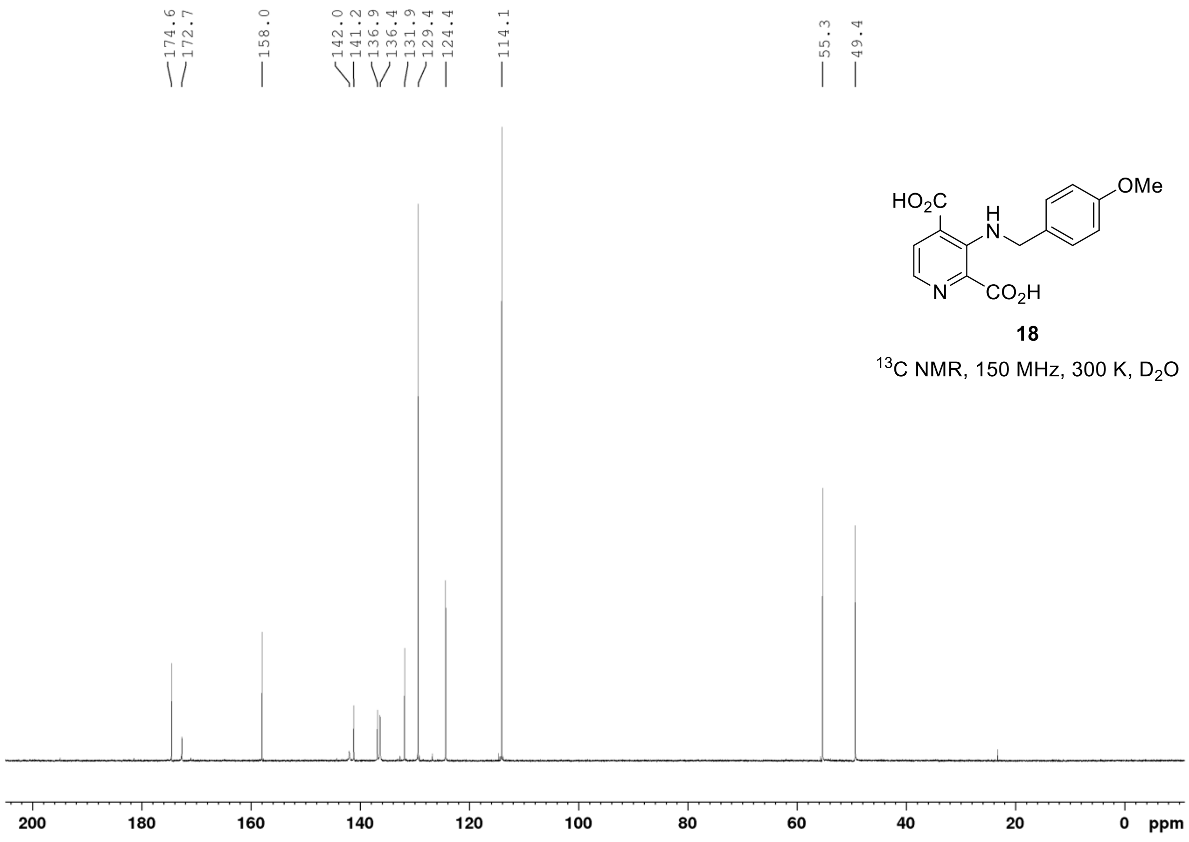

18

^{13}C NMR, 150 MHz, 300 K, D_2O

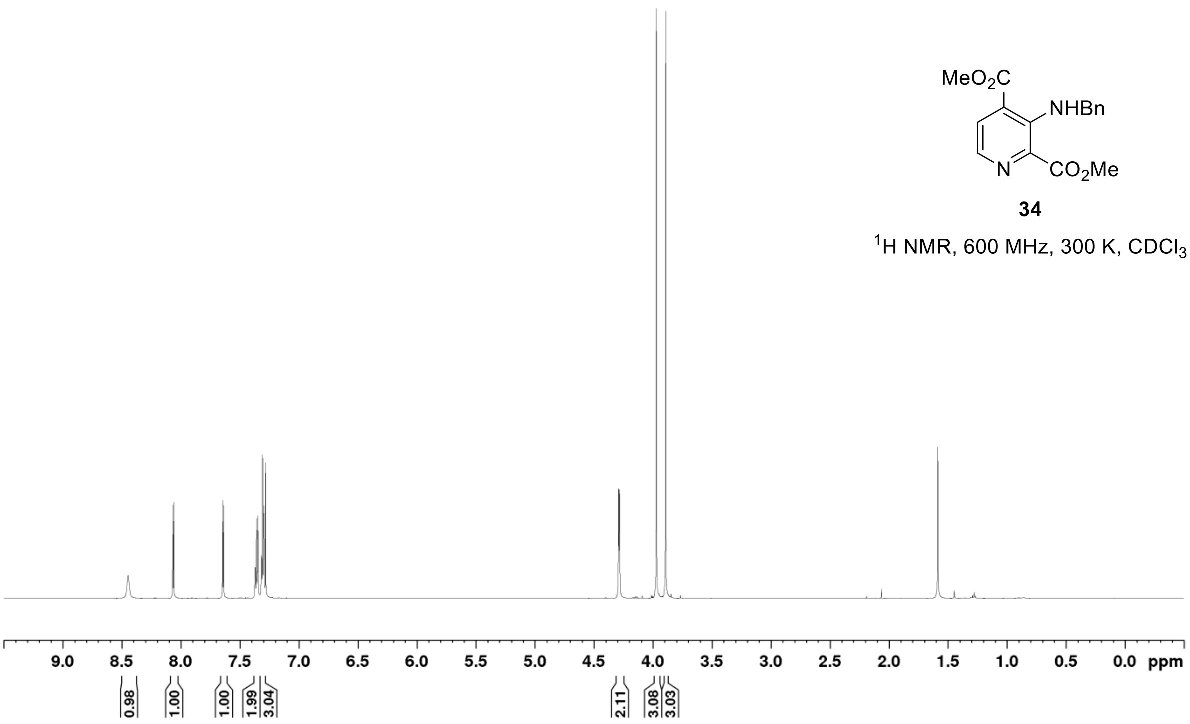

34

^{1}H NMR, 600 MHz, 300 K, $CDCl_3$

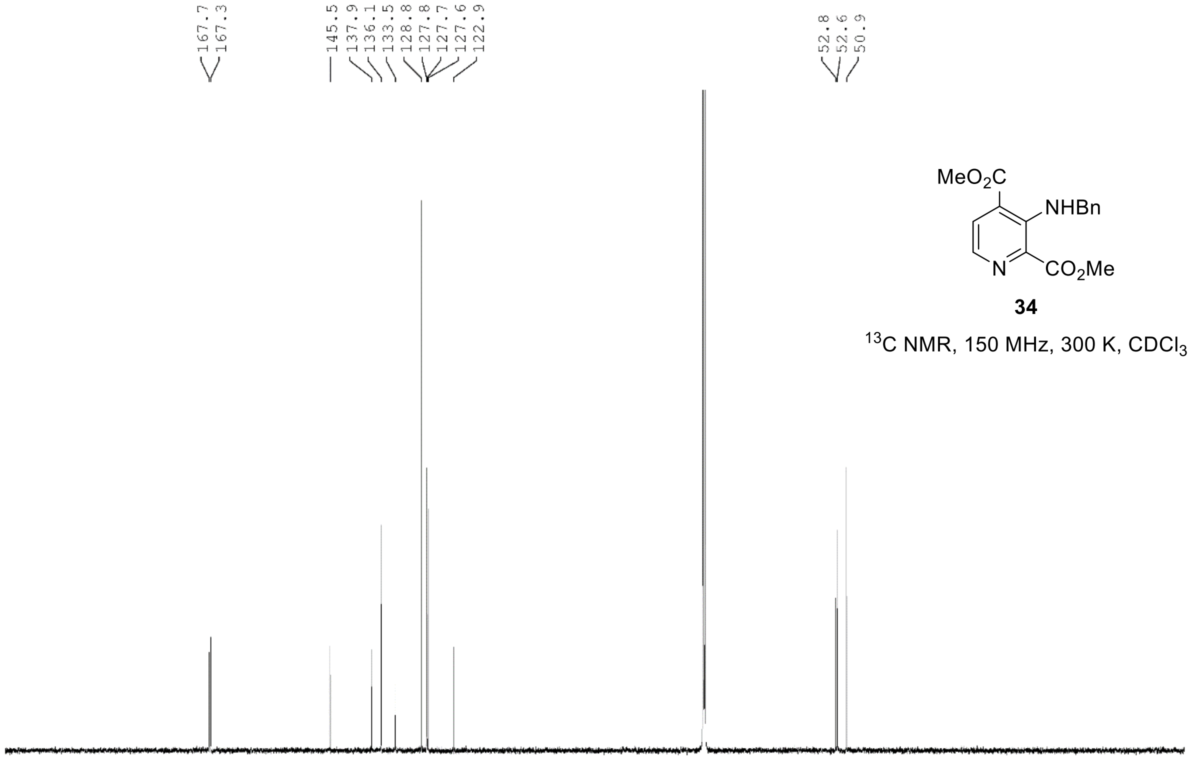

34

^{13}C NMR, 150 MHz, 300 K, $CDCl_3$

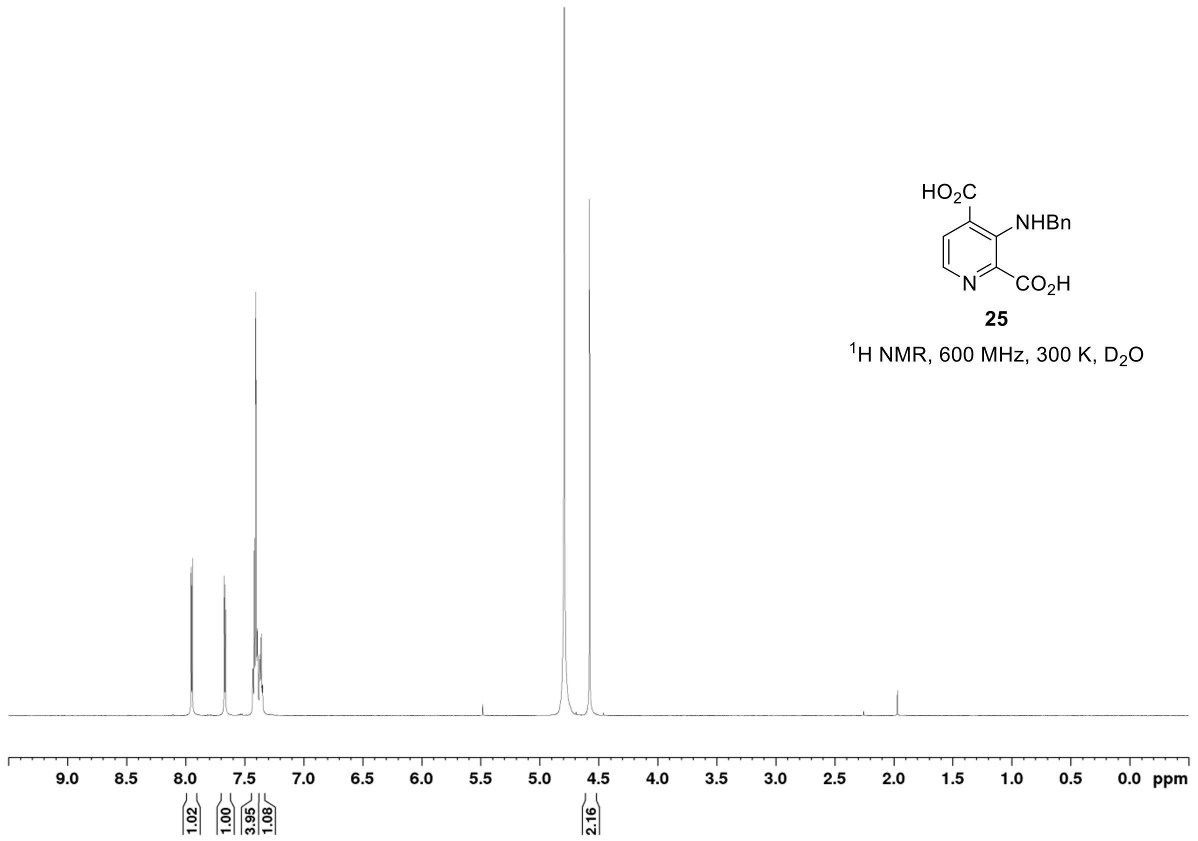

25

^{1}H NMR, 600 MHz, 300 K, D_2O

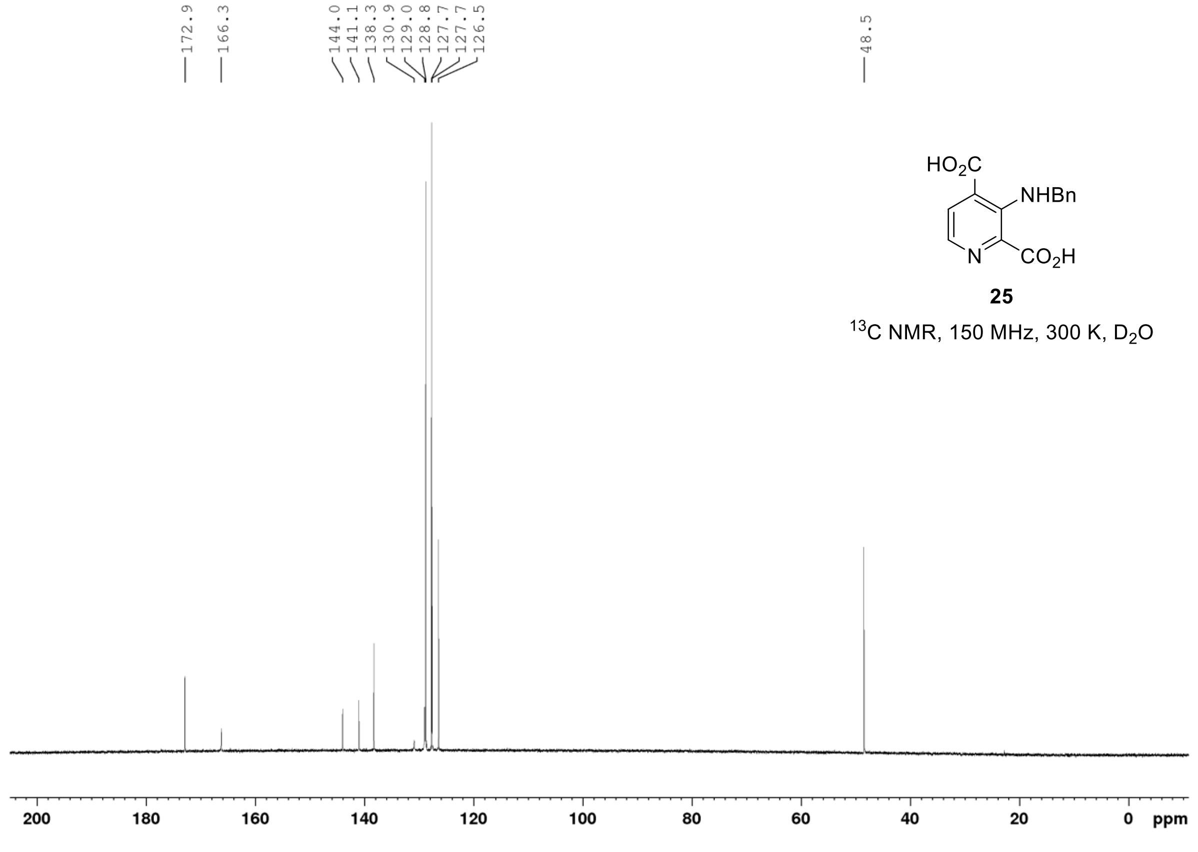

25

^{13}C NMR, 150 MHz, 300 K, D_2O

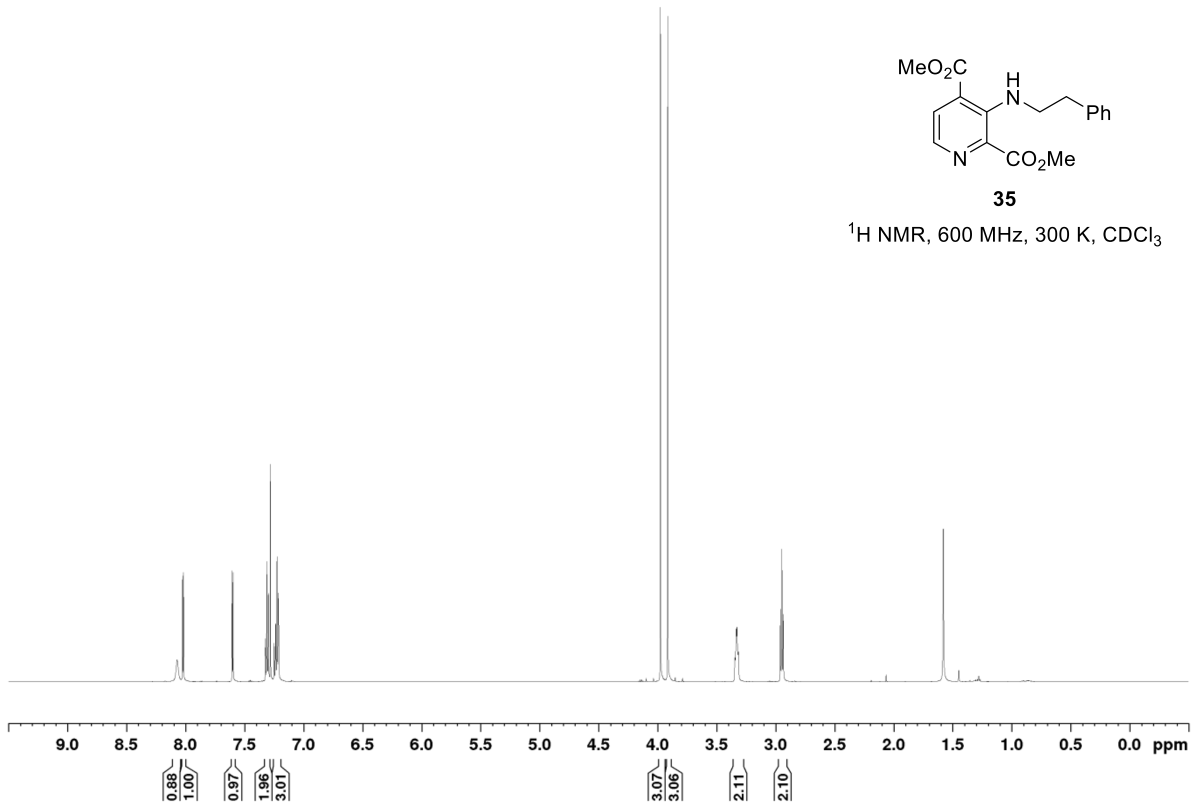

35

^{1}H NMR, 600 MHz, 300 K, $CDCl_3$

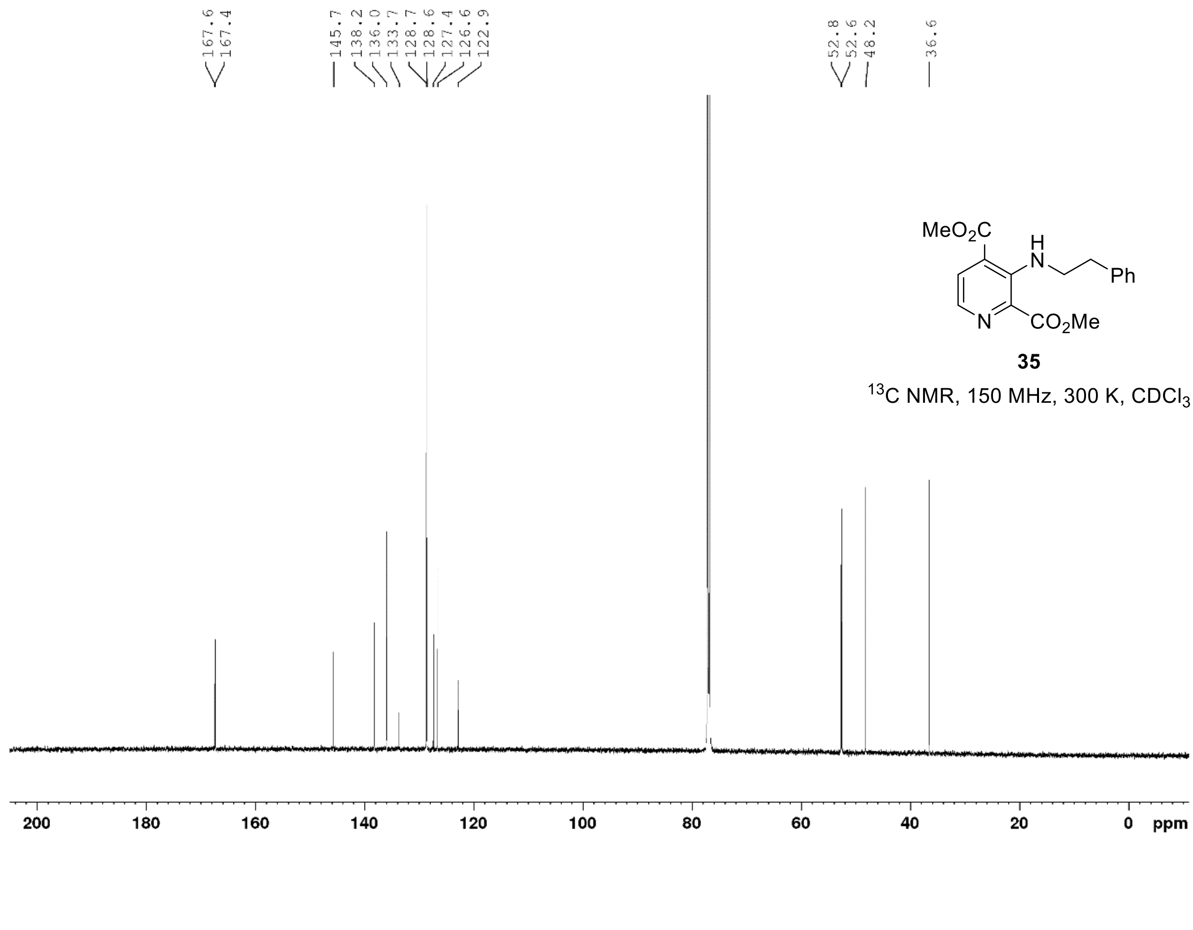

35

^{13}C NMR, 150 MHz, 300 K, $CDCl_3$

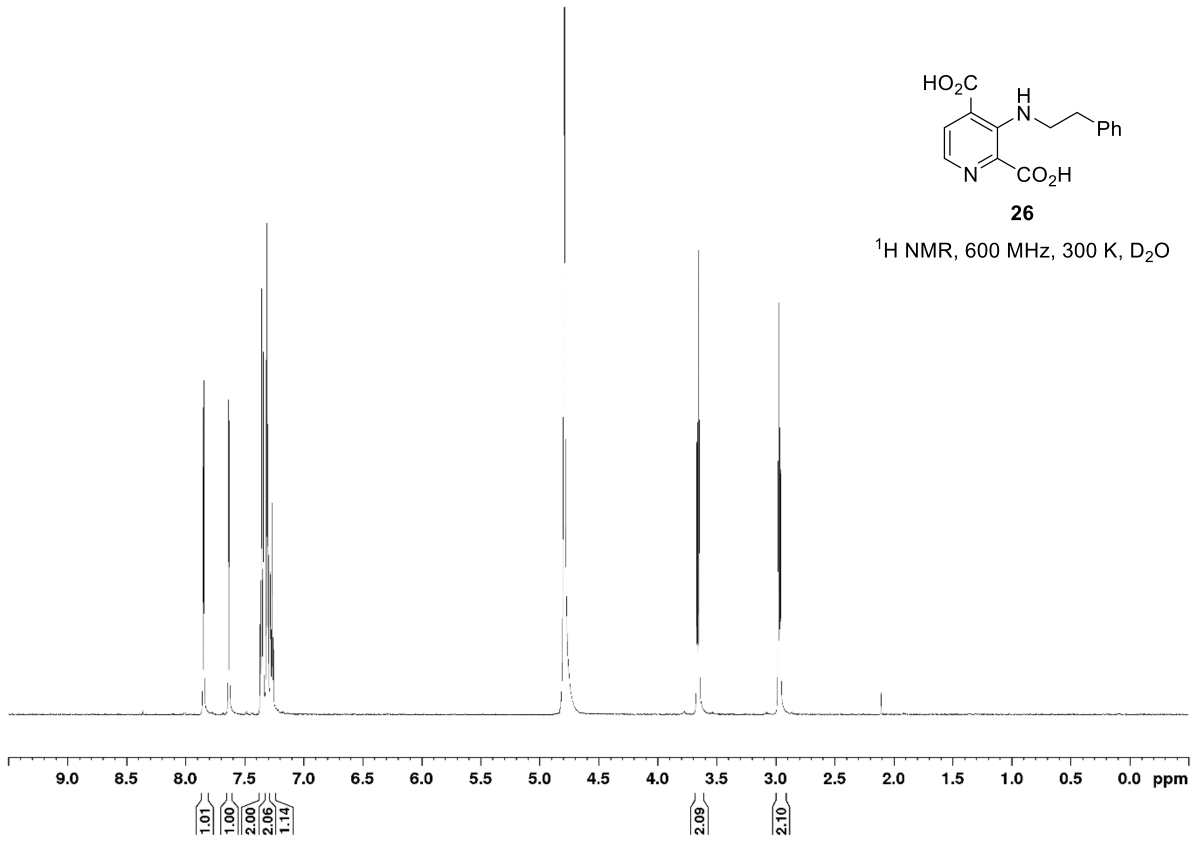
HO2C
H
N
Ph
N
CO2H
26
1H NMR, 600 MHz, 300 K, D2O
9.0 8.5 8.0 7.5 7.0 6.5 6.0 5.5 5.0 4.5 4.0 3.5 3.0 2.5 2.0 1.5 1.0 0.5 0.0 ppm
1.01
1.00
2.00
2.06
1.14
2.09
2.10

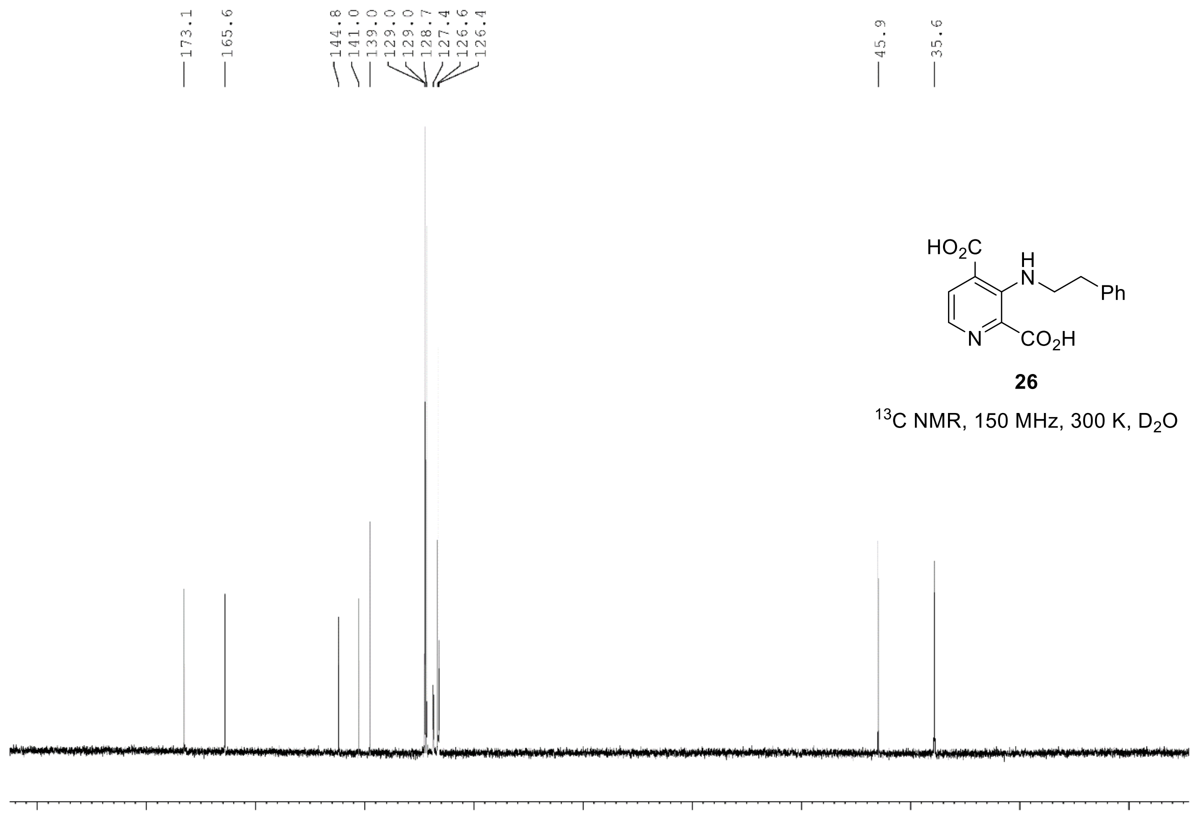
173.1
165.6
144.8
141.0
139.0
129.0
129.0
128.7
127.4
126.6
126.4
45.9
35.6
HO2C
H
N
Ph
N
CO2H
26
13C NMR, 150 MHz, 300 K, D2O
200 180 160 140 120 100 80 60 40 20 0 ppm

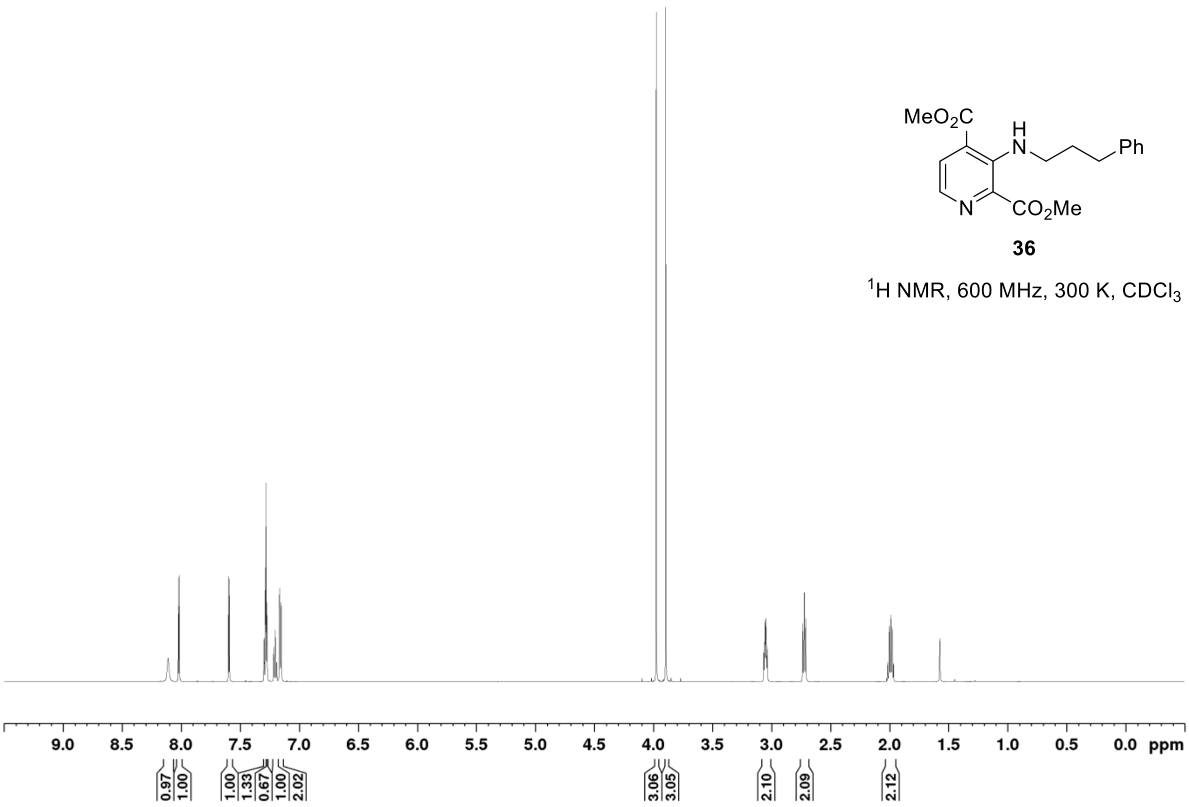

36

^{1}H NMR, 600 MHz, 300 K, $CDCl_3$

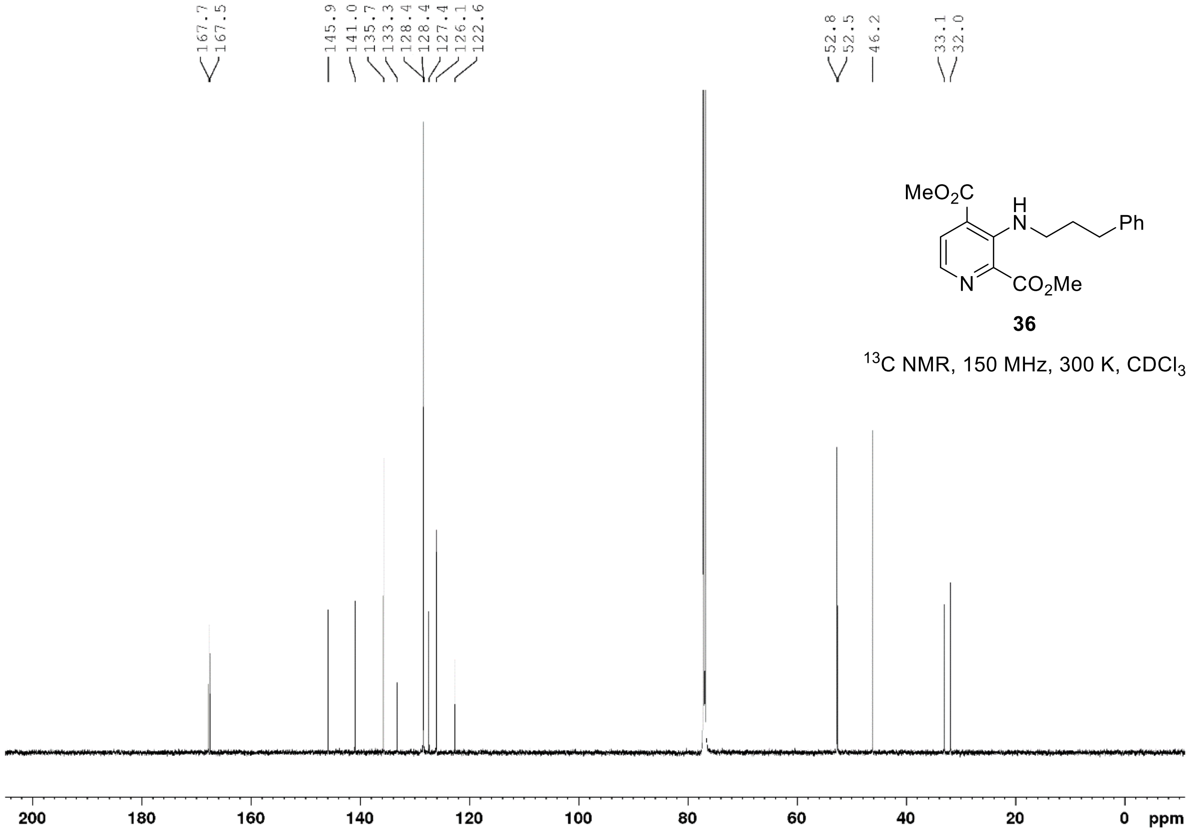

36

^{13}C NMR, 150 MHz, 300 K, $CDCl_3$

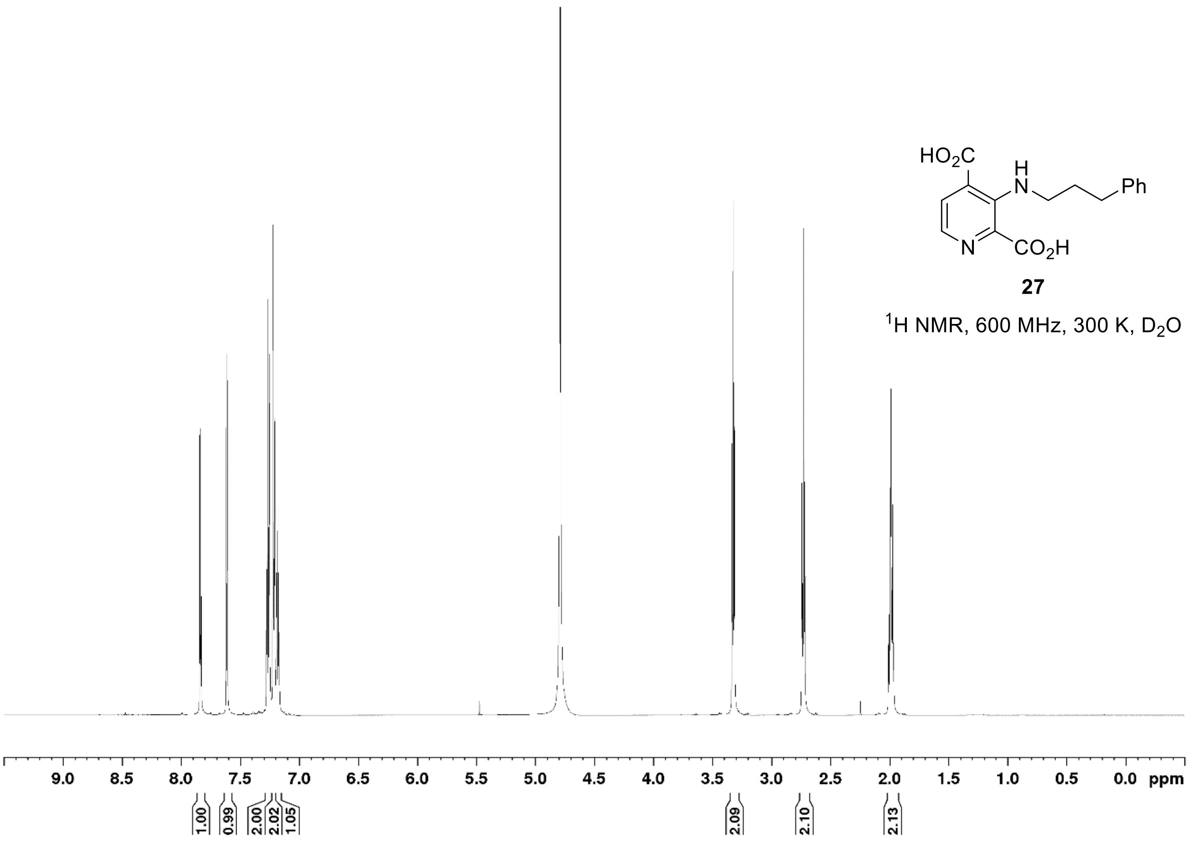

27

^{1}H NMR, 600 MHz, 300 K, D_2O

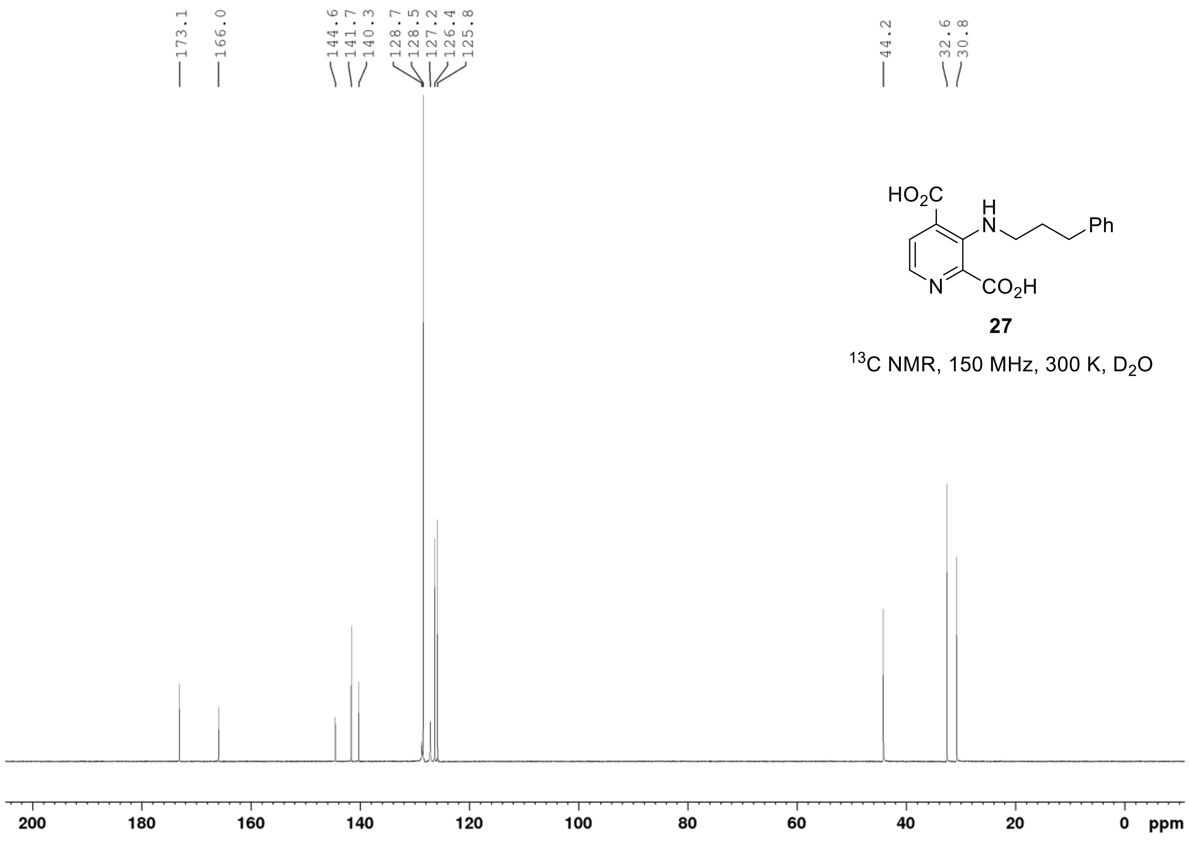

27

^{13}C NMR, 150 MHz, 300 K, D_2O

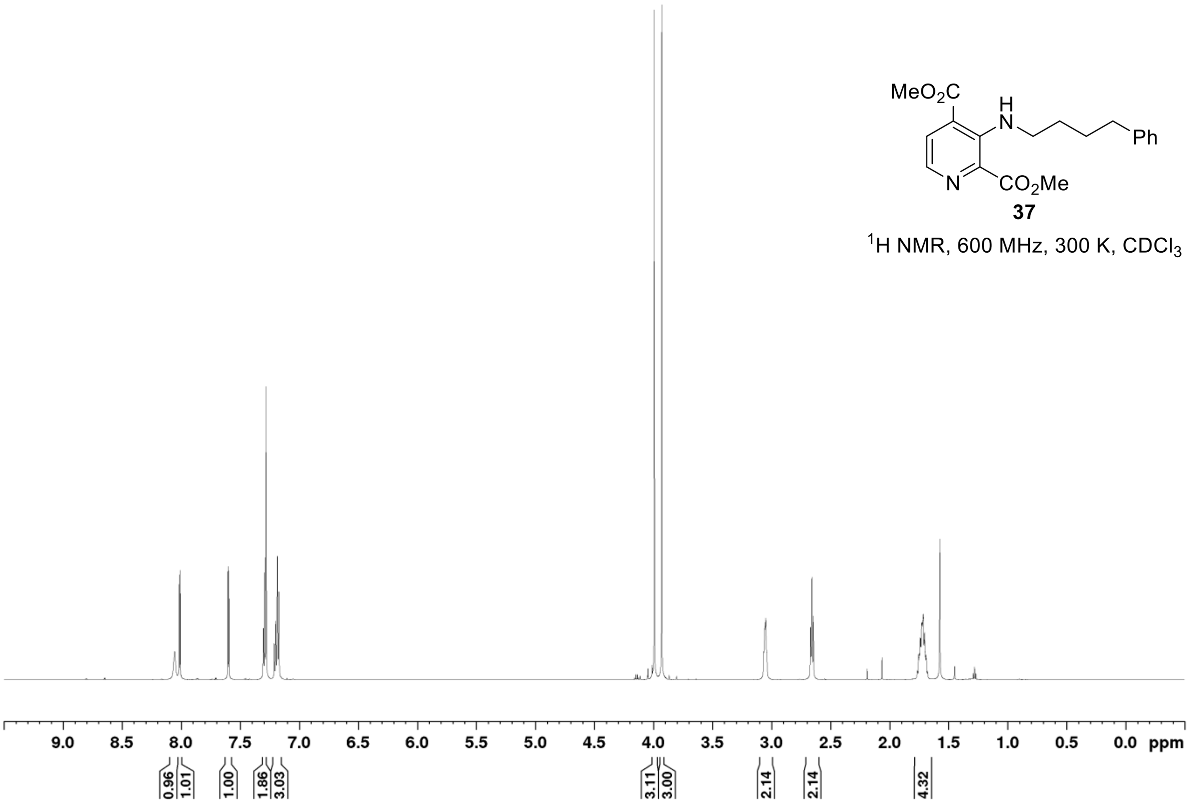

37

^{1}H NMR, 600 MHz, 300 K, $CDCl_3$

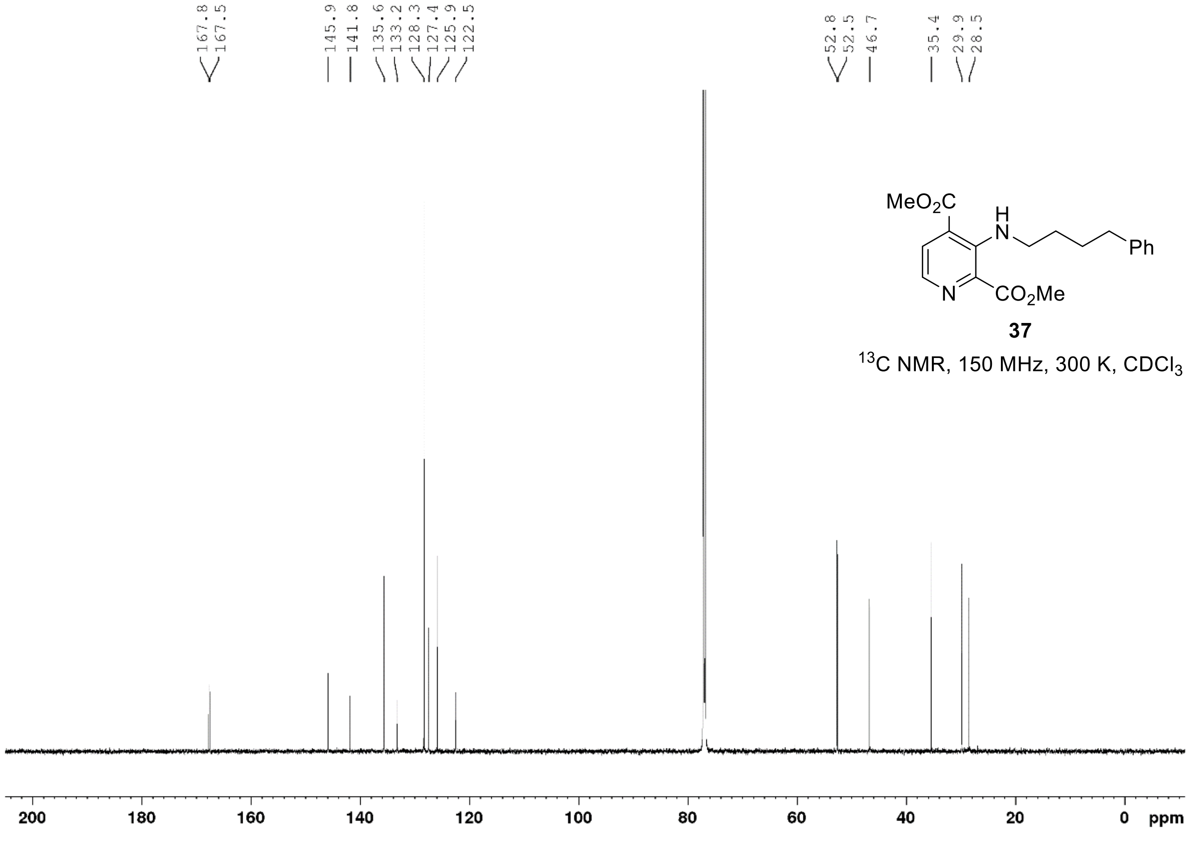

37

^{13}C NMR, 150 MHz, 300 K, $CDCl_3$

28

^{1}H NMR, 600 MHz, 300 K, D_2O

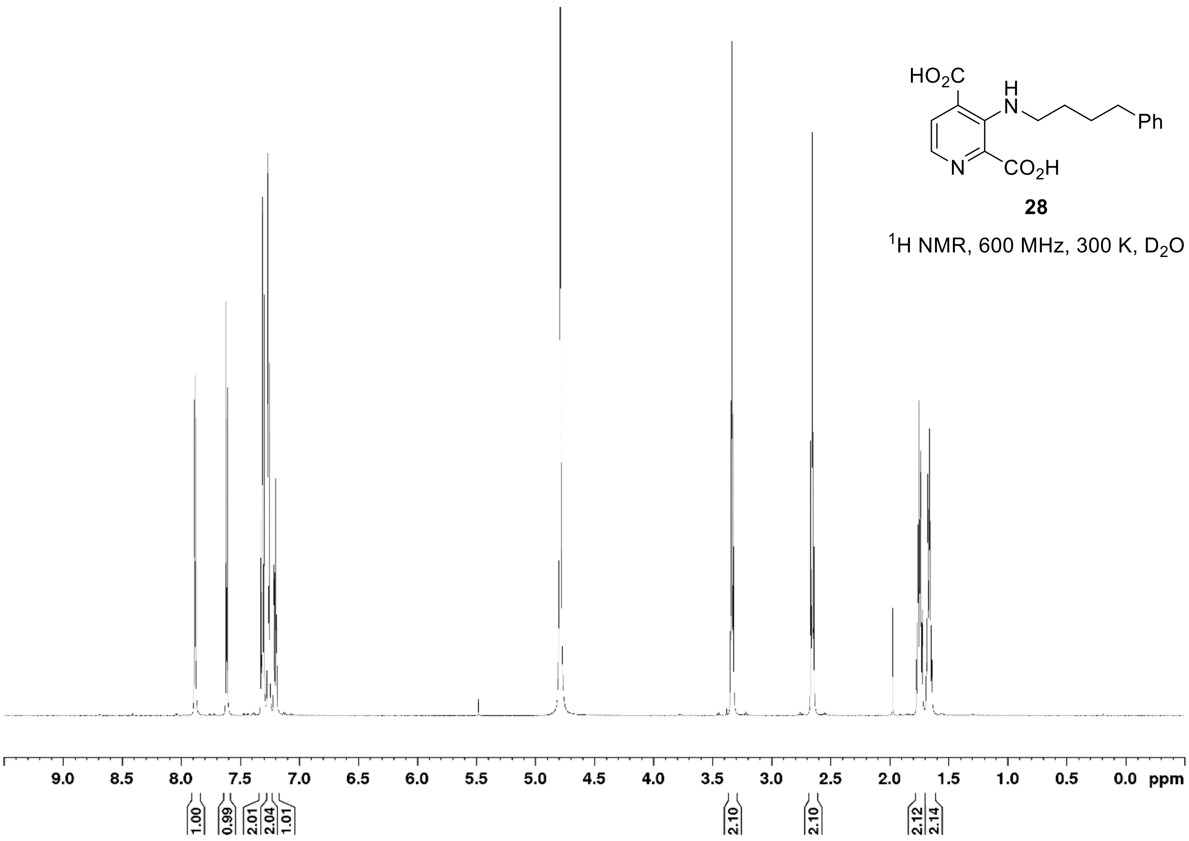

28

^{13}C NMR, 150 MHz, 300 K, D_2O

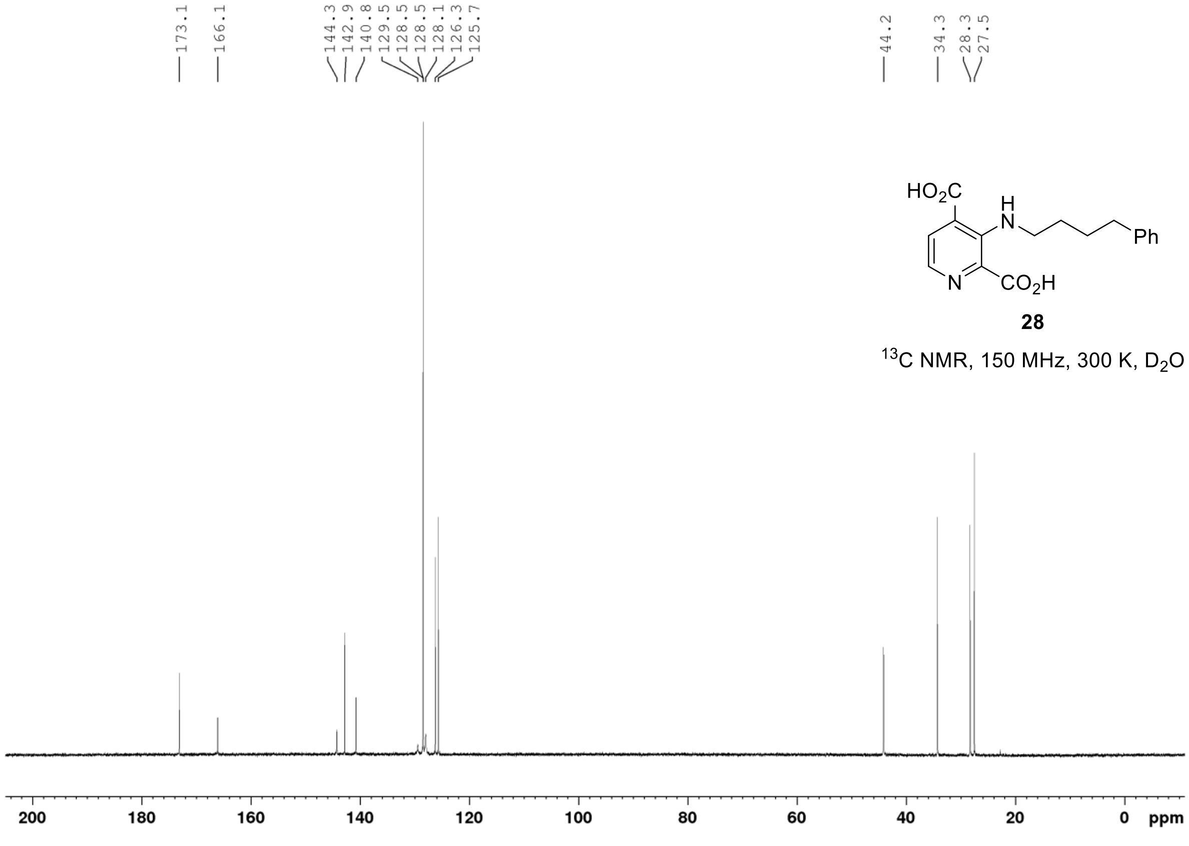

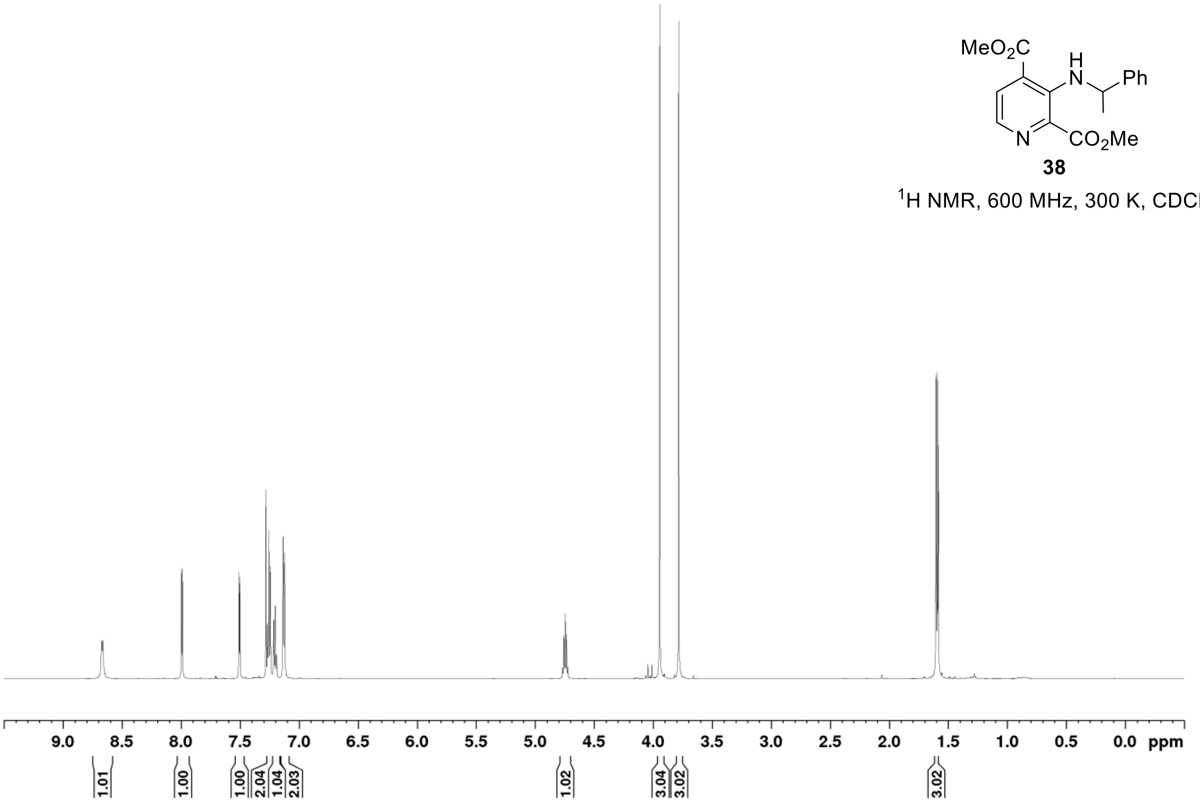

38

^{1}H NMR, 600 MHz, 300 K, $CDCl_3$

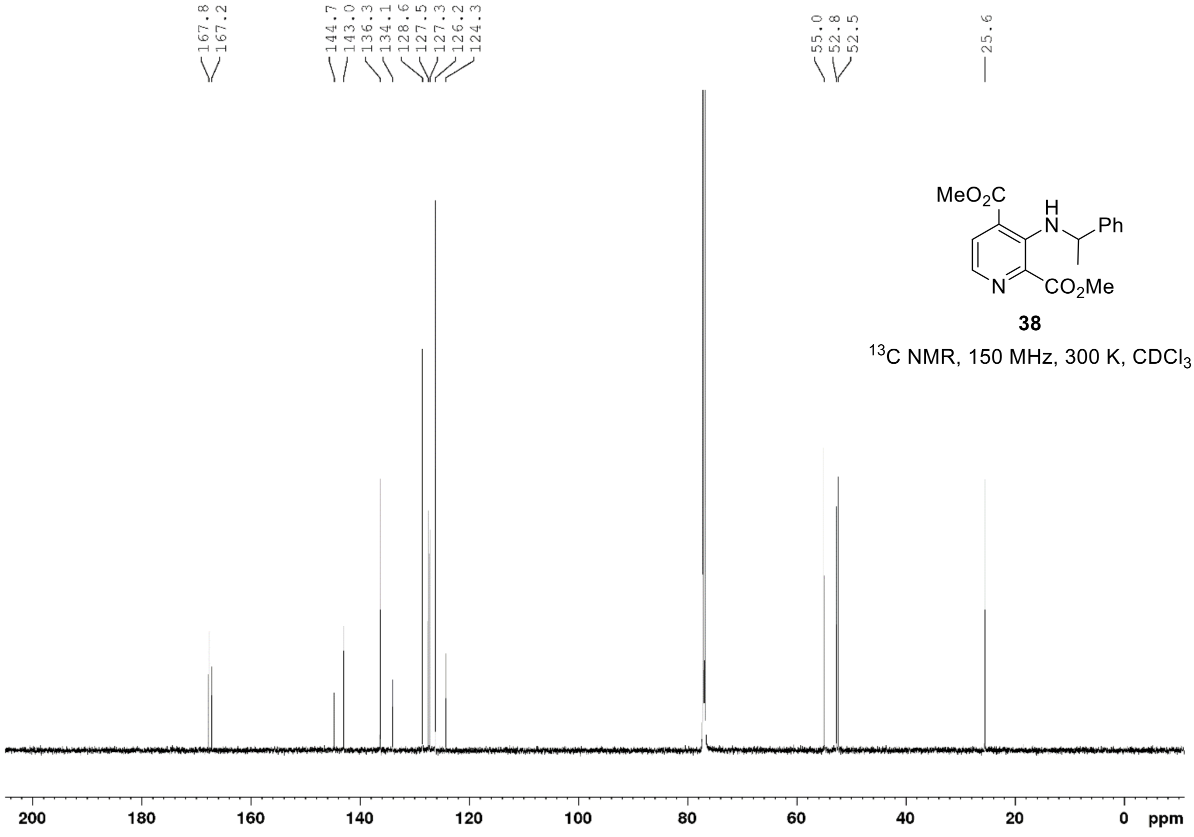

38

^{13}C NMR, 150 MHz, 300 K, $CDCl_3$

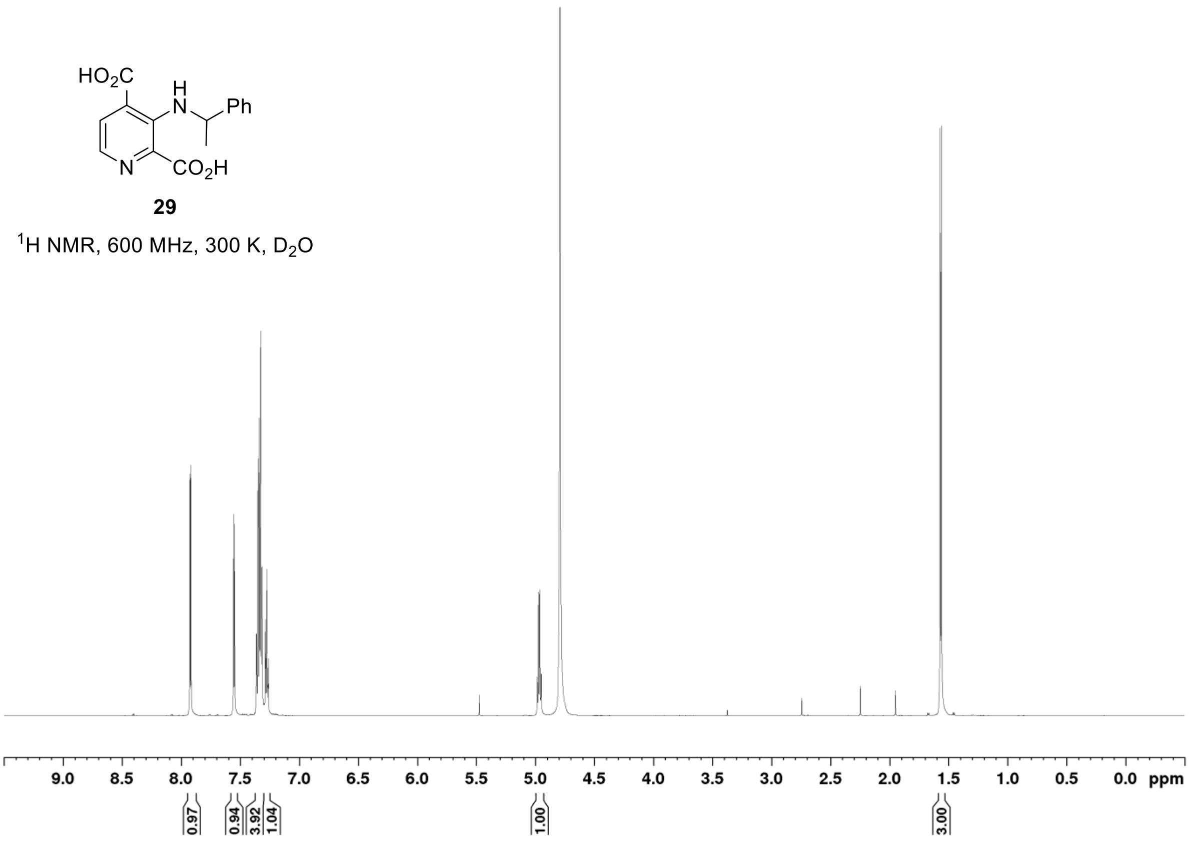

29

^{1}H NMR, 600 MHz, 300 K, D_2O

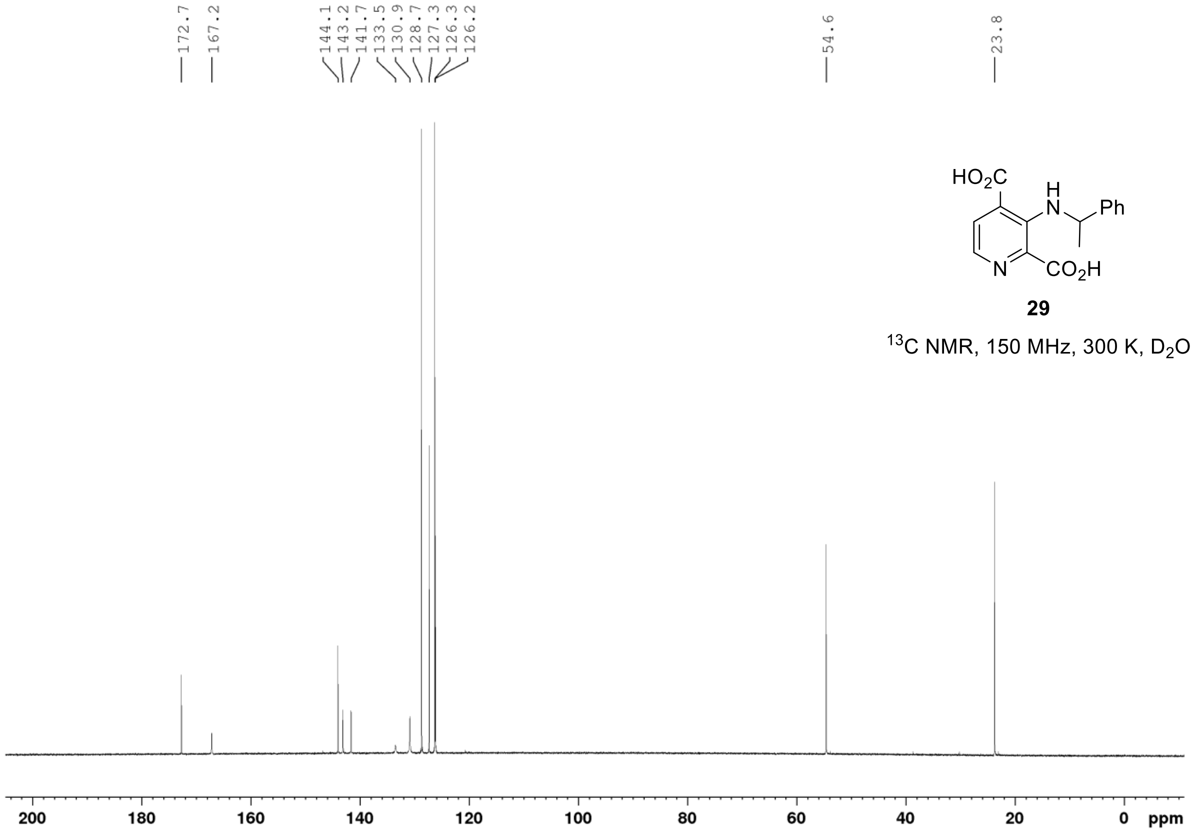

29

^{13}C NMR, 150 MHz, 300 K, D_2O

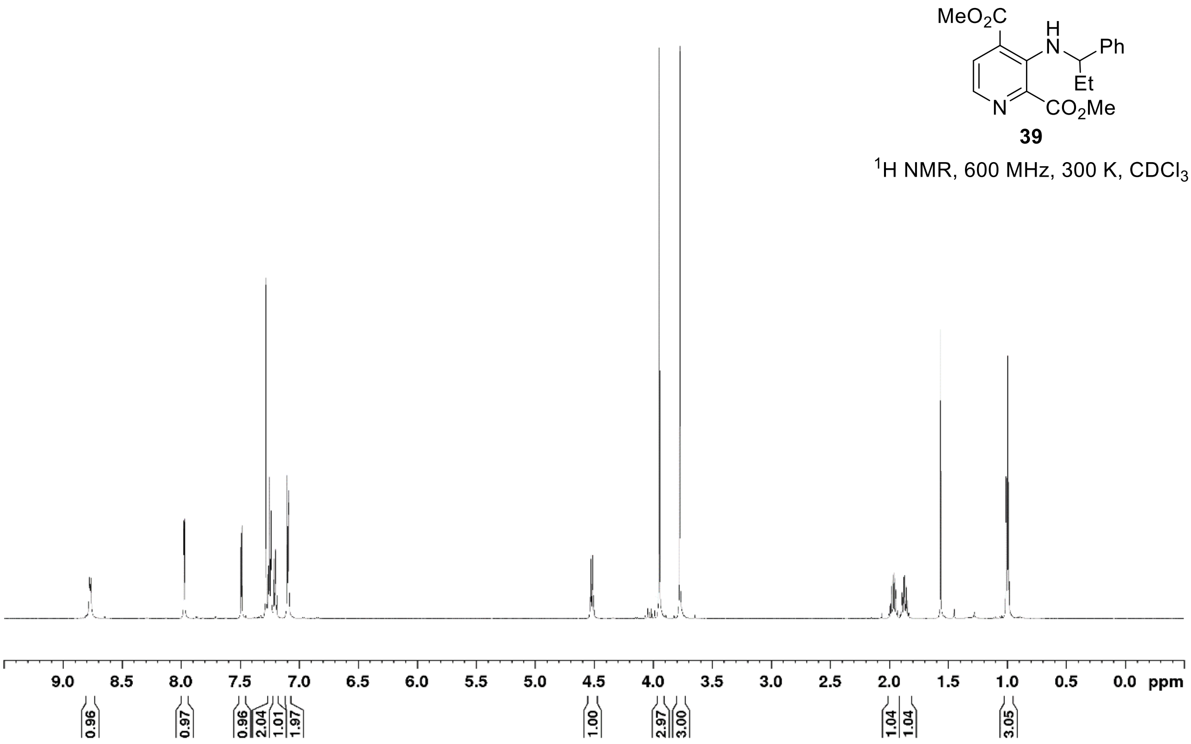

39

^{1}H NMR, 600 MHz, 300 K, $CDCl_3$

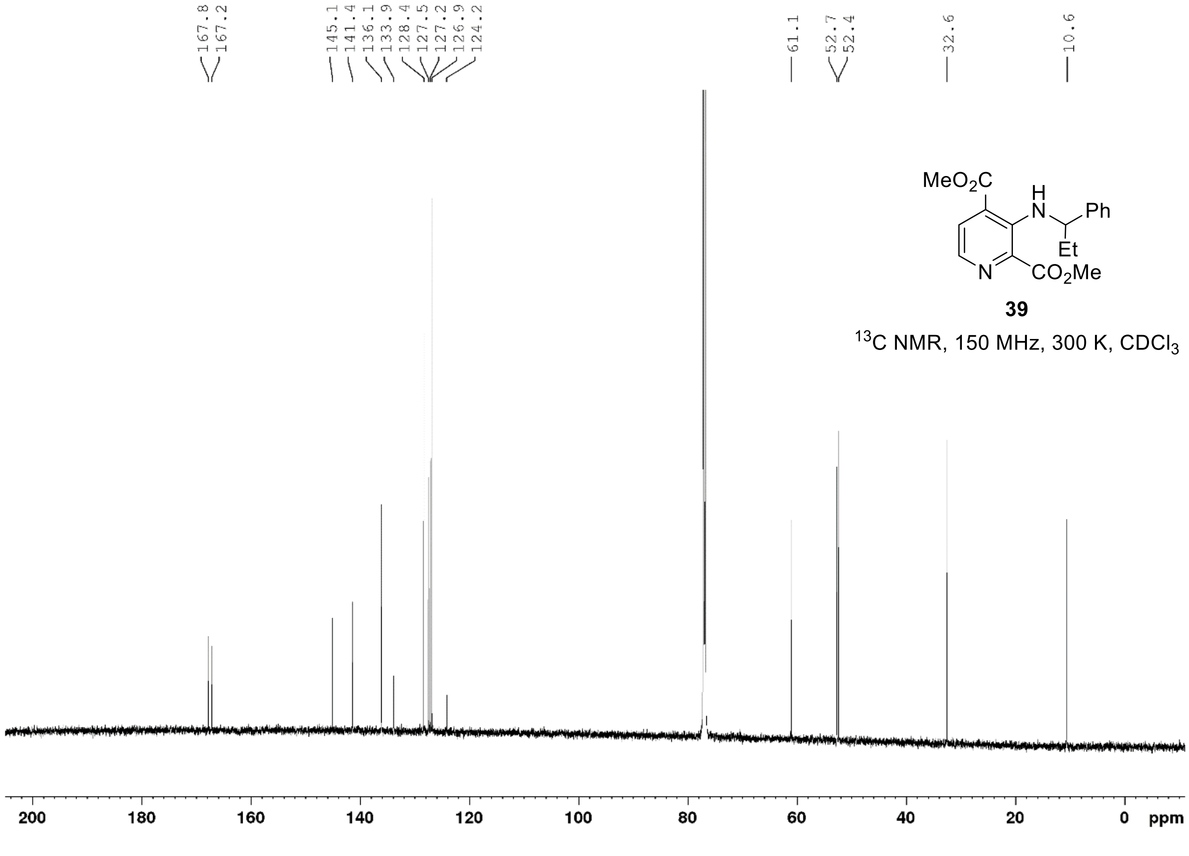

39

^{13}C NMR, 150 MHz, 300 K, $CDCl_3$

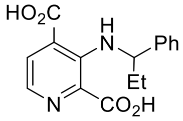

30

^{1}H NMR, 600 MHz, 300 K, D_2O

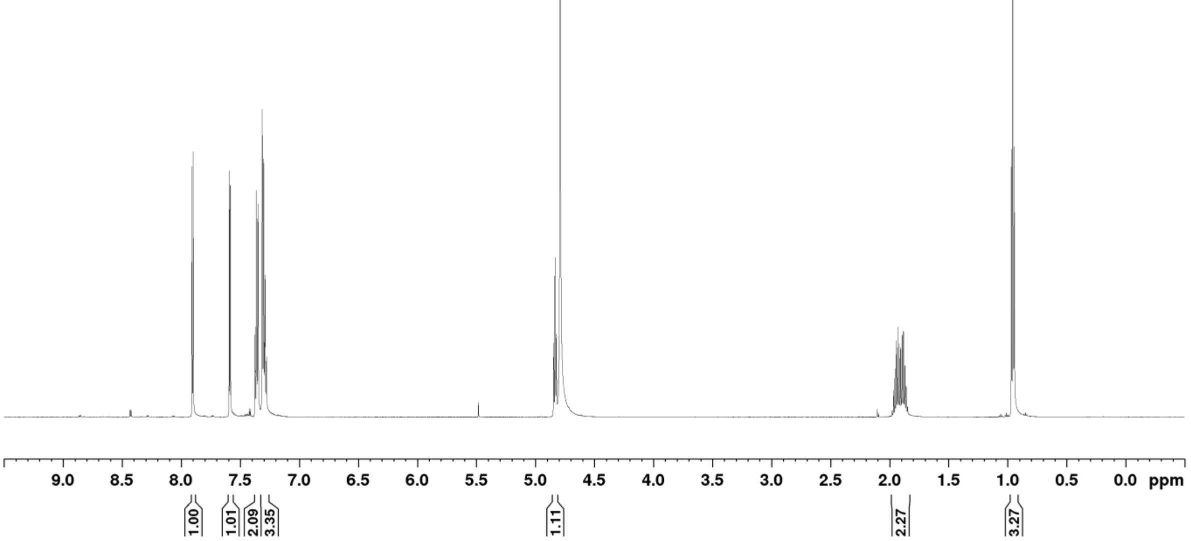

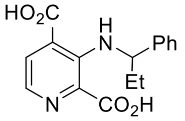

30

^{13}C NMR, 150 MHz, 300 K, D_2O

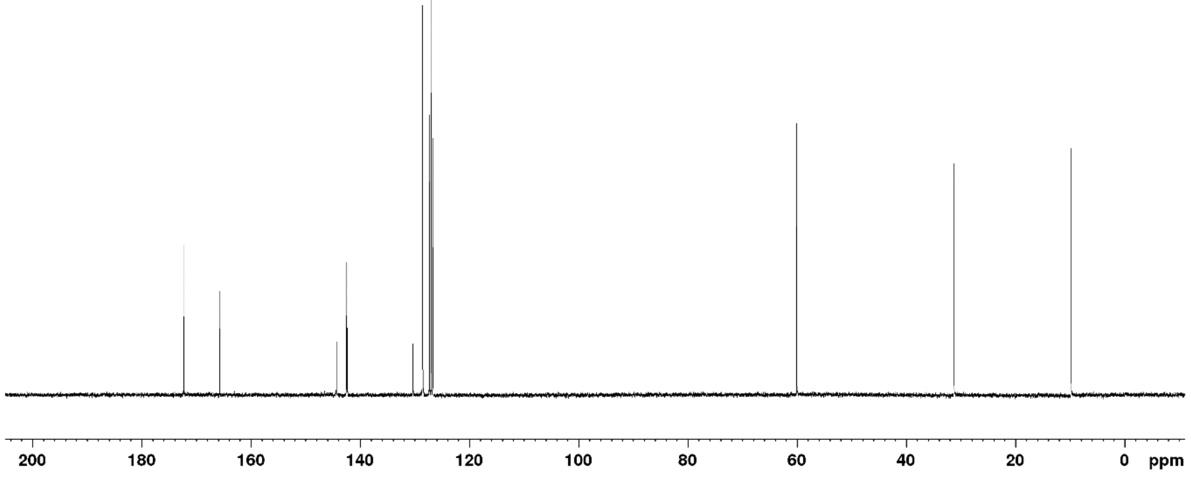

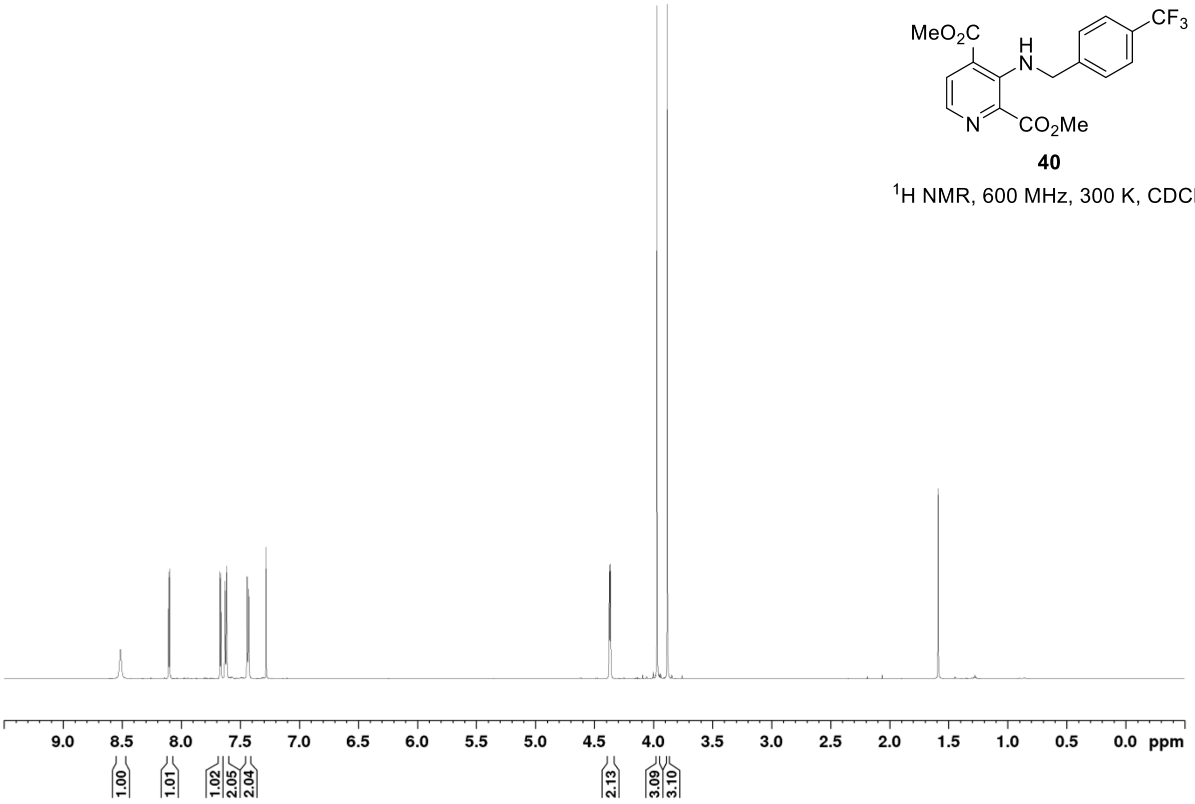

40

^{1}H NMR, 600 MHz, 300 K, $CDCl_3$

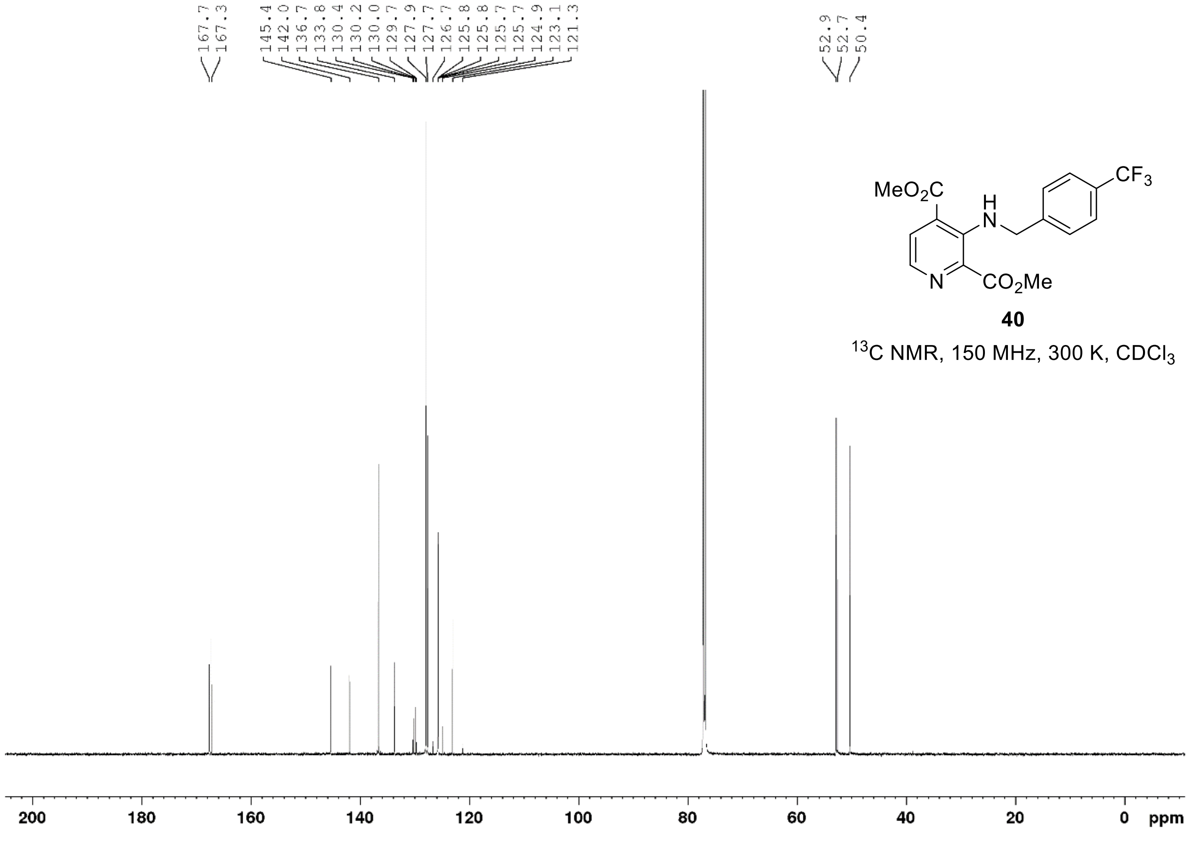

40

^{13}C NMR, 150 MHz, 300 K, $CDCl_3$

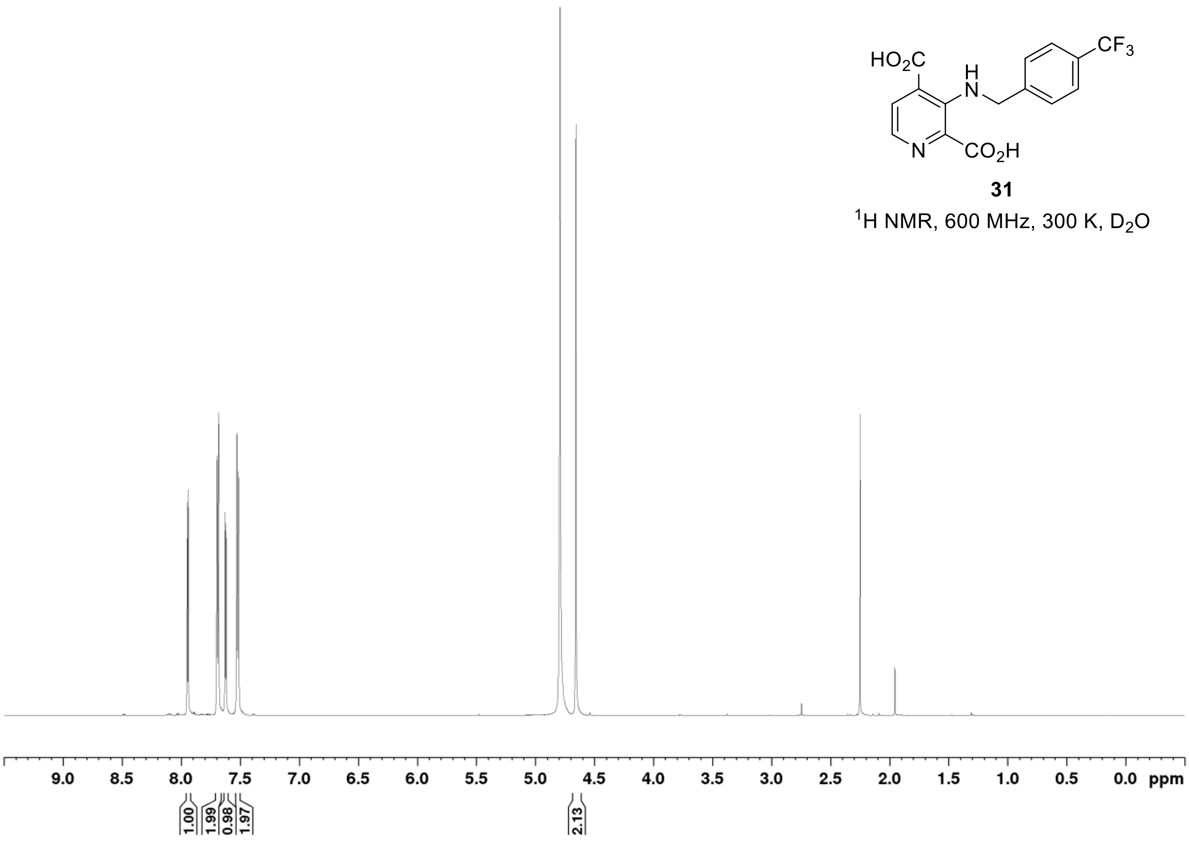

31

^{1}H NMR, 600 MHz, 300 K, D_2O

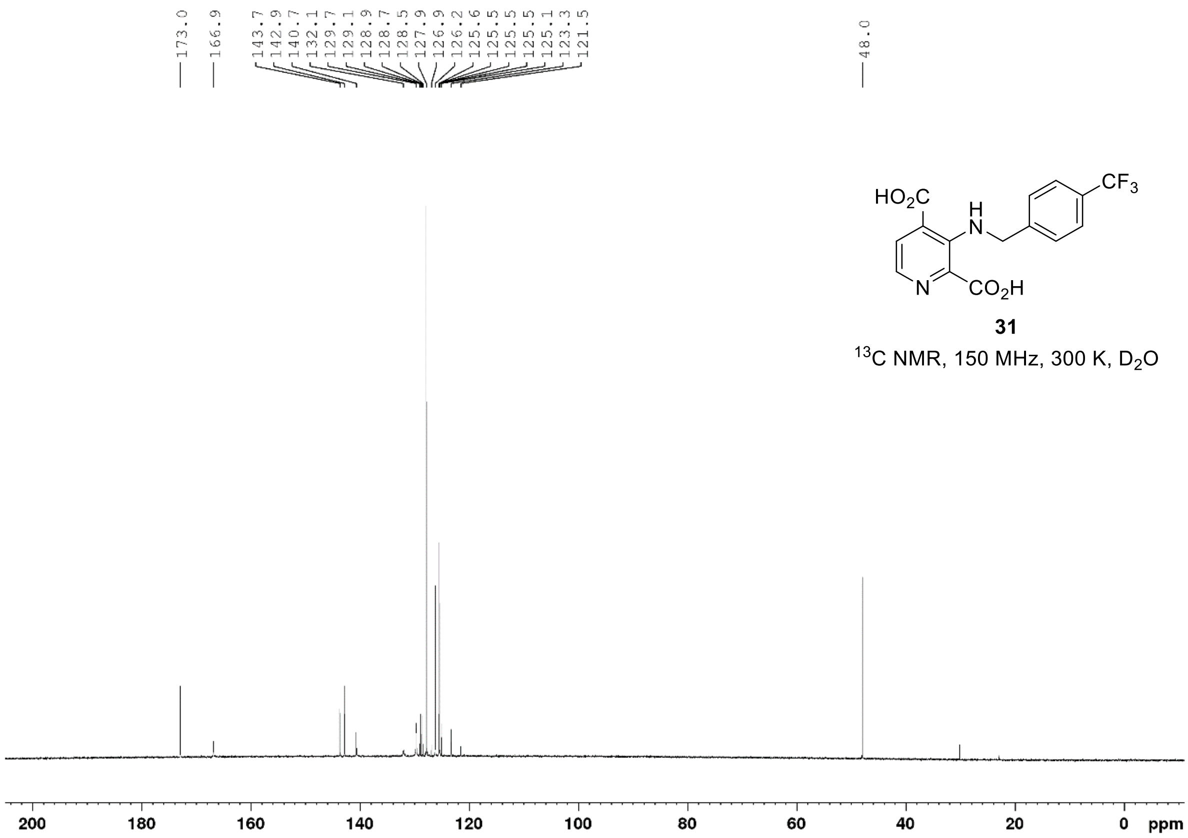

31

^{13}C NMR, 150 MHz, 300 K, D_2O

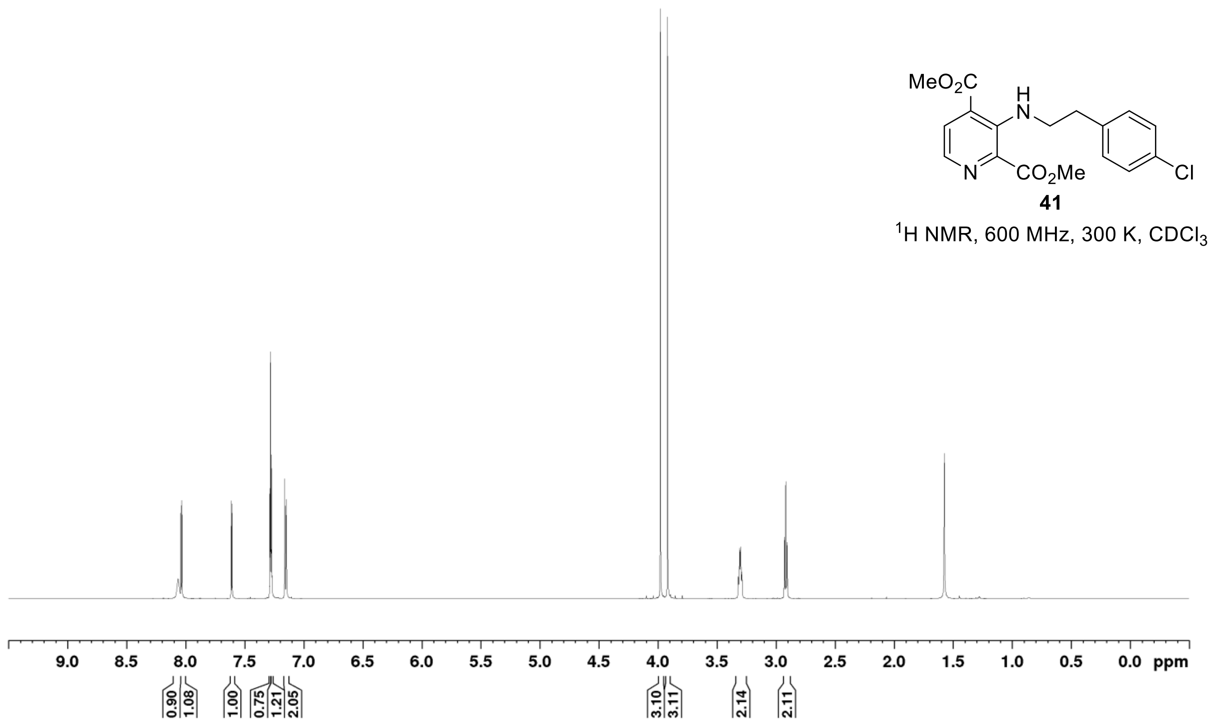
MeO2C
H
N
N
CO2Me
Cl
41
1H NMR, 600 MHz, 300 K, CDCl3
9.0 8.5 8.0 7.5 7.0 6.5 6.0 5.5 5.0 4.5 4.0 3.5 3.0 2.5 2.0 1.5 1.0 0.5 0.0 ppm
0.90 1.08 1.00 0.75 1.21 2.05 3.10 3.11 2.14 2.11

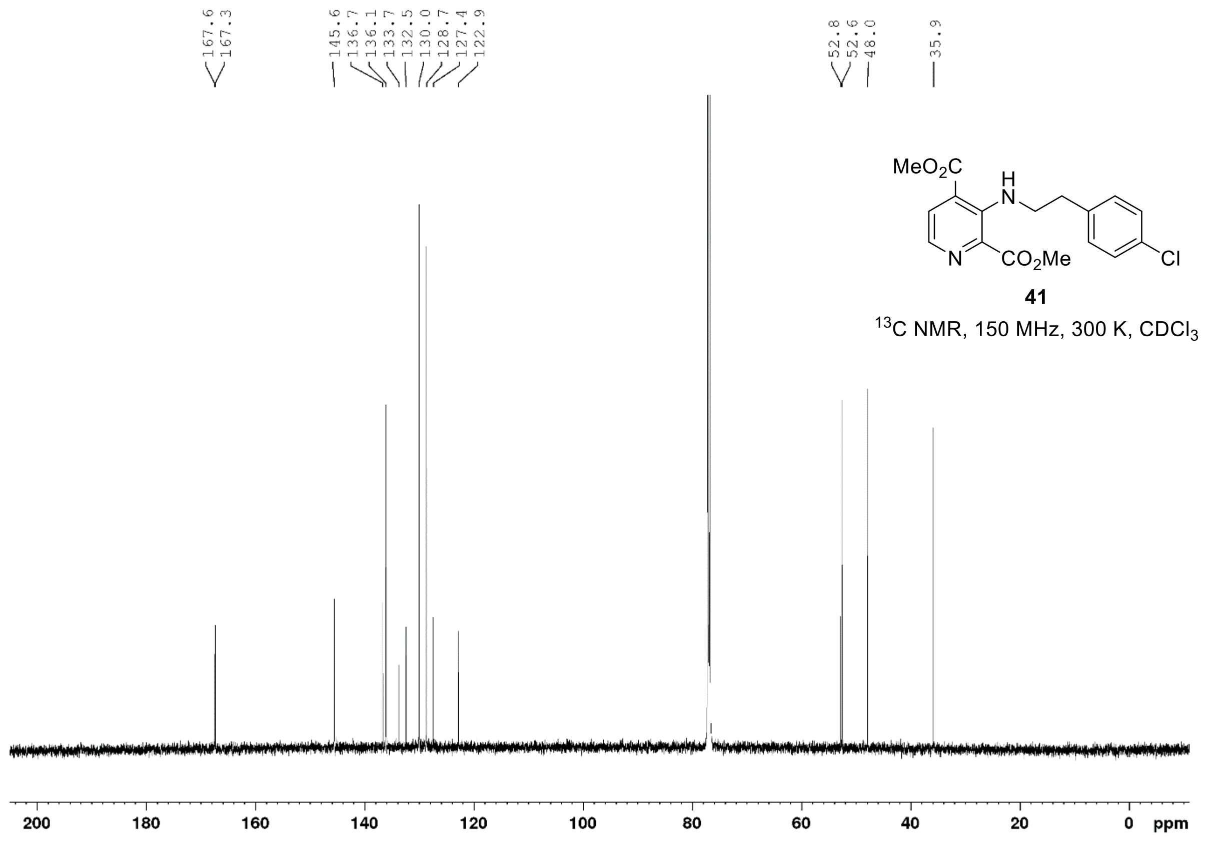
167.6 167.3 145.6 136.7 136.1 133.7 132.5 130.0 128.7 127.4 122.9 52.8 52.6 48.0 35.9
MeO2C
H
N
N
CO2Me
Cl
41
13C NMR, 150 MHz, 300 K, CDCl3
200 180 160 140 120 100 80 60 40 20 0 ppm

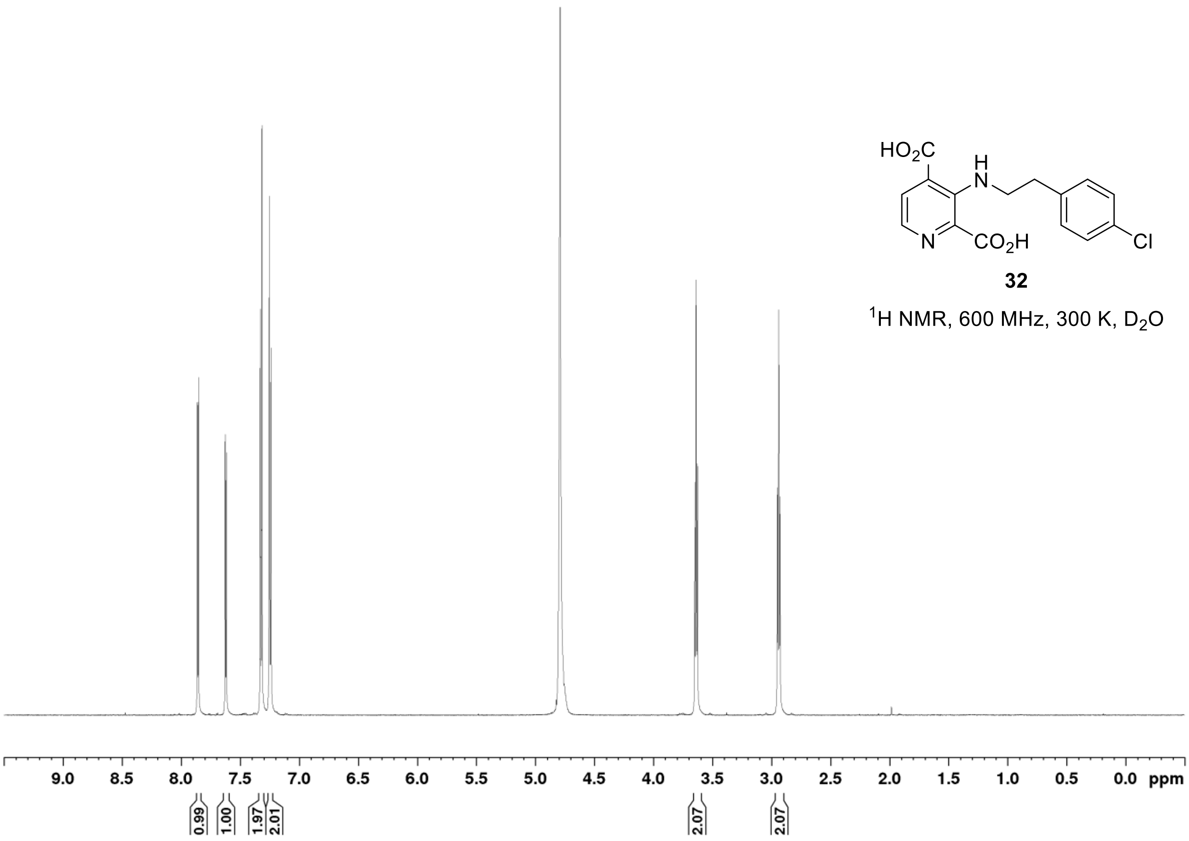

32

^{1}H NMR, 600 MHz, 300 K, D_2O

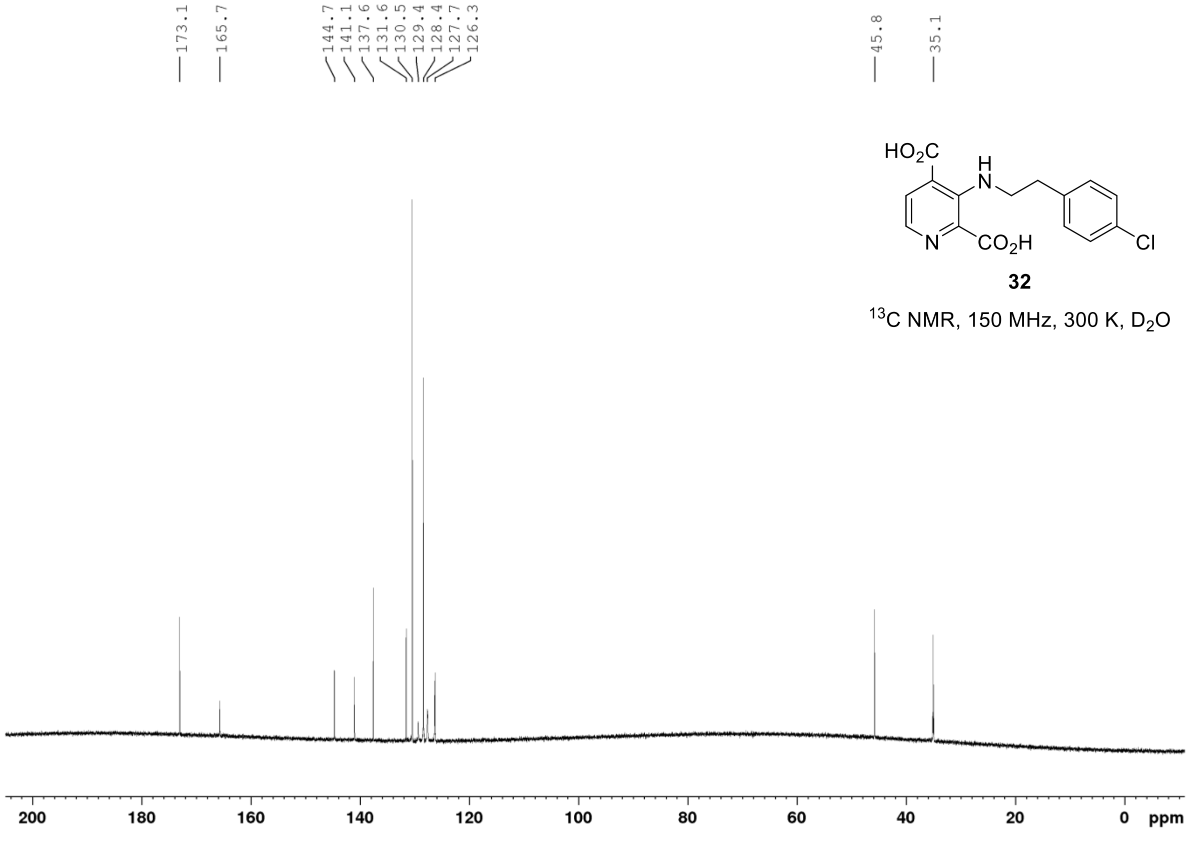

32

^{13}C NMR, 150 MHz, 300 K, D_2O

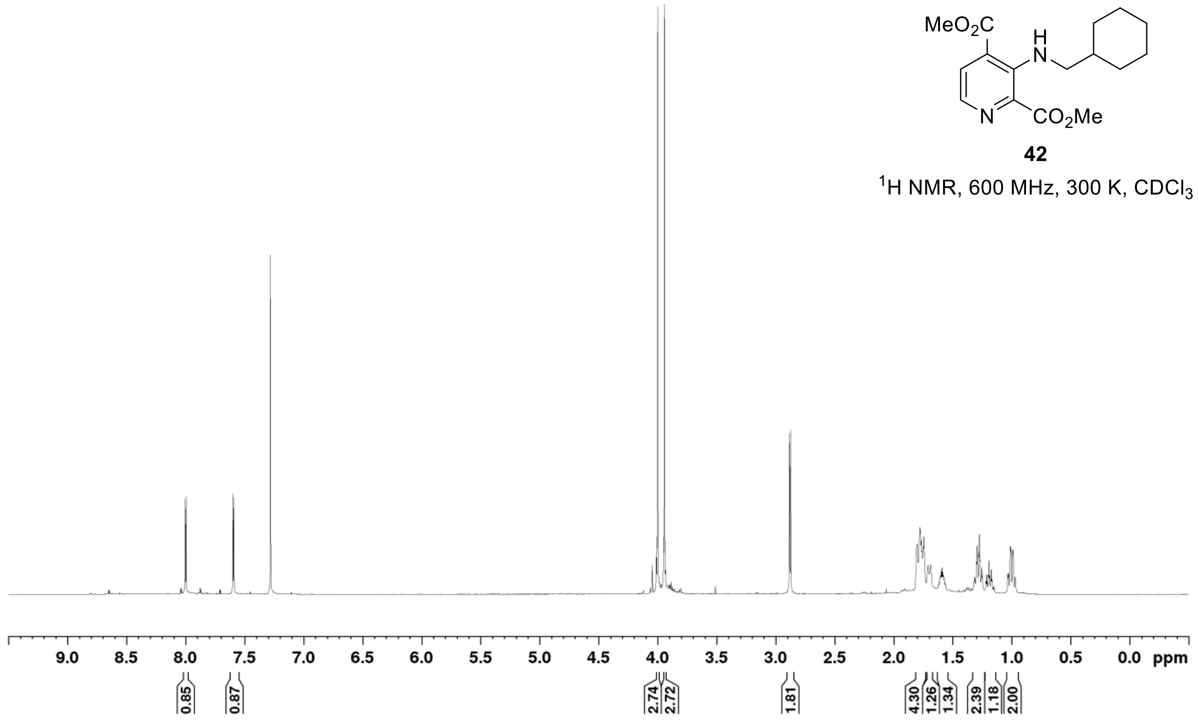

42

^{1}H NMR, 600 MHz, 300 K, $CDCl_3$

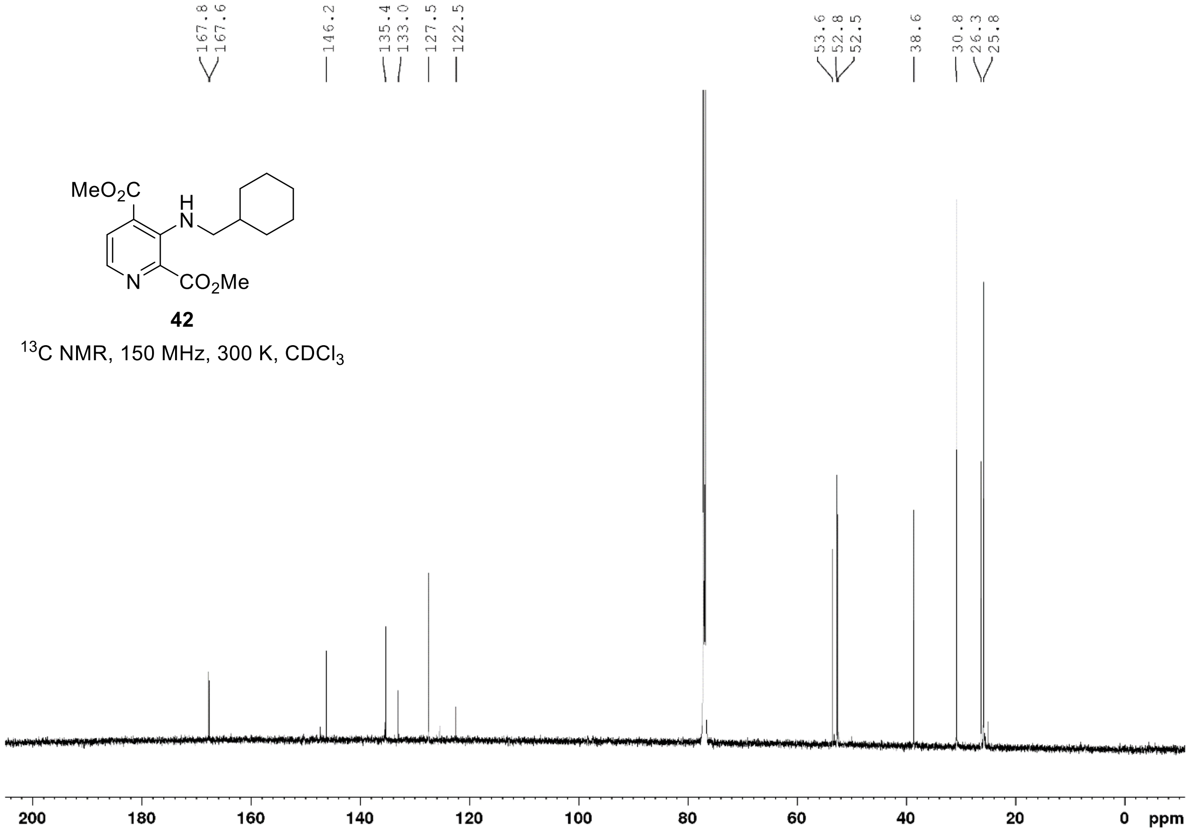

42

^{13}C NMR, 150 MHz, 300 K, $CDCl_3$

33

^{1}H NMR, 600 MHz, 300 K, D_2O

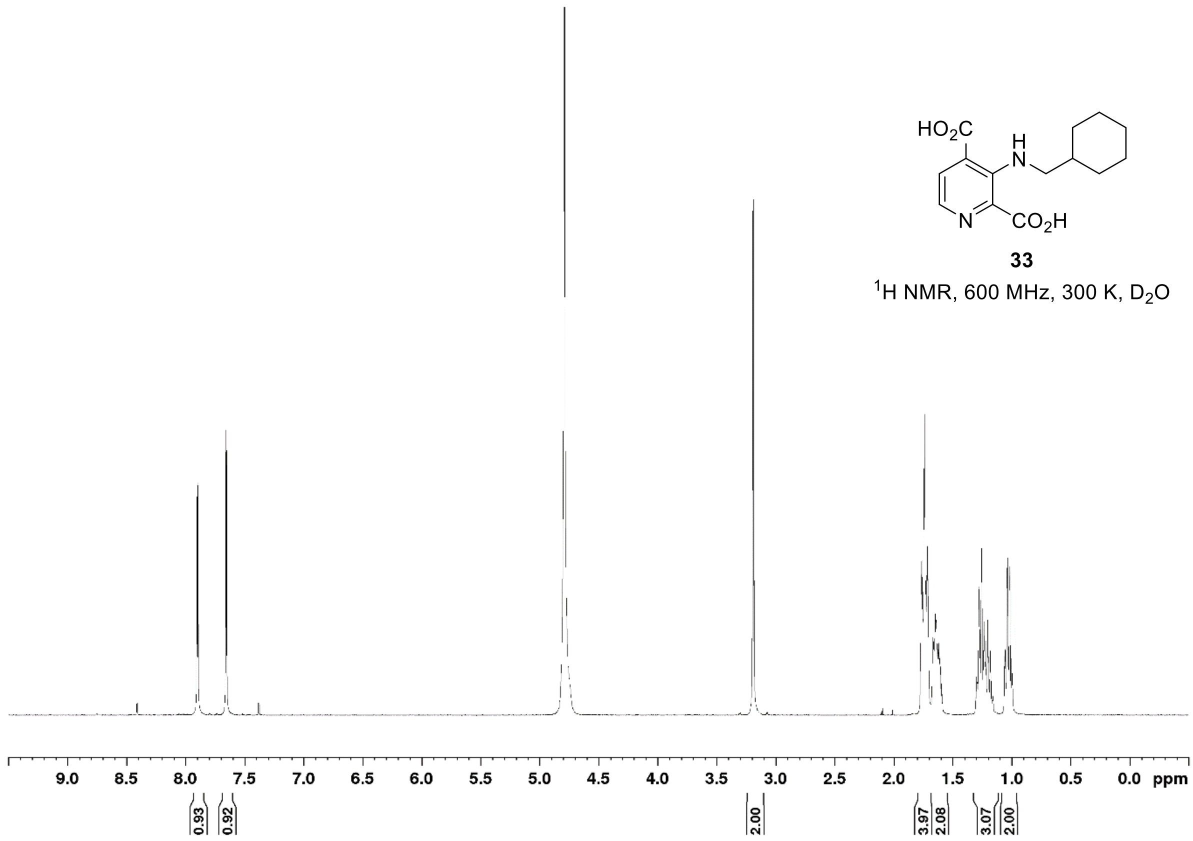

33

^{13}C NMR, 150 MHz, 300 K, D_2O

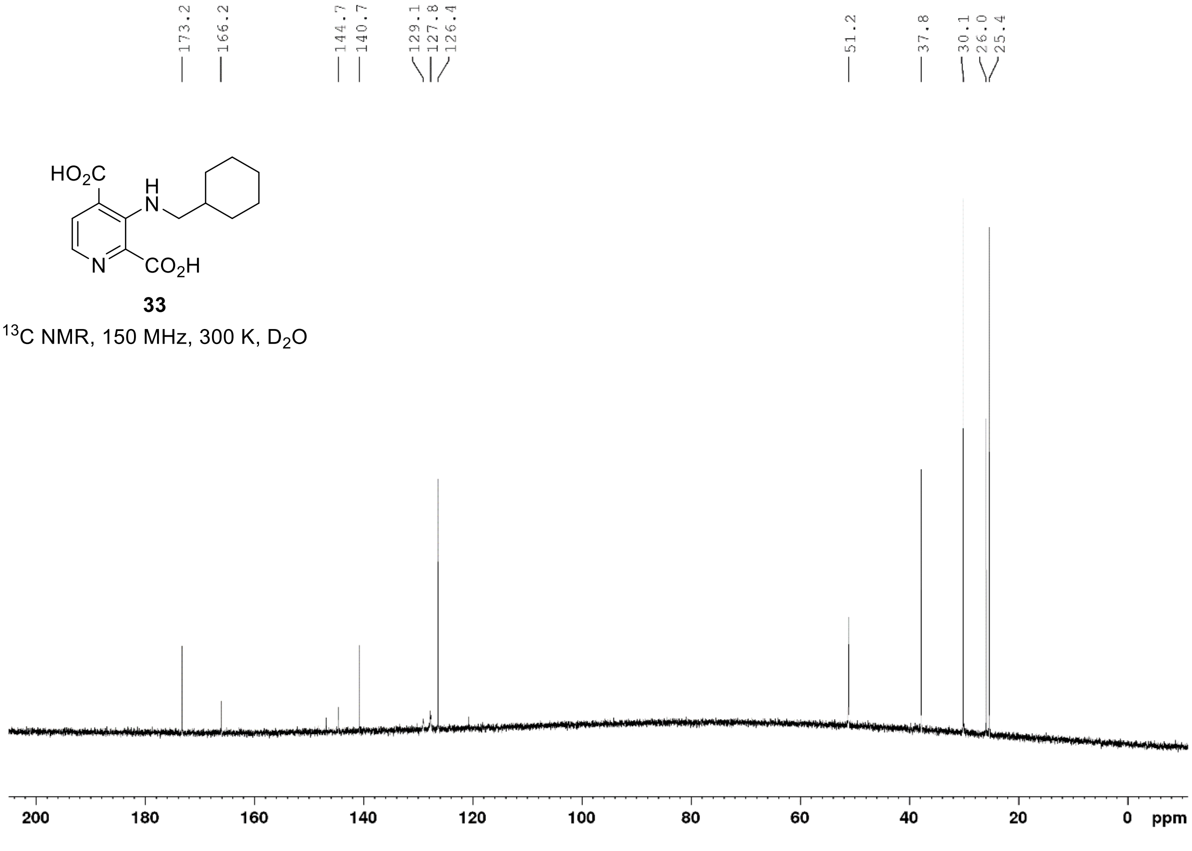

Supplement: Supplementary file 1 — Supplementary [file CMDC-15-1139-s001.pdf]
